# Rational Design of Azastatin as a Potential ADC Payload with Reduced Bystander Killing

**DOI:** 10.1002/cmdc.202000497

**Published:** 2020-10-16

**Authors:** Rafael W. Hartmann, Raphael Fahrner, Denys Shevshenko, Mårten Fyrknäs, Rolf Larsson, Fredrik Lehmann, Luke R. Odell

**Affiliations:** ^1^ Department of Medicinal Chemistry Uppsala University Box 574 75123 Uppsala Sweden; ^2^ Synthesis Division Recipharm OT Chemistry Virdings allé 32b 75450 Uppsala Sweden; ^3^ Department of Medical Sciences Cancer Pharmacology and Computational Medicine Uppsala University University Hospital 75185 Uppsala Sweden

**Keywords:** Total synthesis, Medicinal chemistry, Cytotoxicity, Diastereoselectivity, Antibodies

## Abstract

Auristatins are a class of ultrapotent microtubule inhibitors, whose growing clinical popularity in oncology is based upon their use as payloads in antibody‐drug conjugates (ADCs). The most widely utilized auristatin, MMAE, has however been shown to cause apoptosis in non‐pathological cells proximal to the tumour (“bystander killing”). Herein, we introduce azastatins, a new class of auristatin derivatives encompassing a side chain amine for antibody conjugation. The synthesis of Cbz‐azastatin methyl ester, which included the C2‐elongation and diastereoselective reduction of two proteinogenic amino acids as key transformations, was accomplished in 22 steps and 0.76 % overall yield. While Cbz‐protected azastatin methyl ester (0.13–3.0 nM) inhibited proliferation more potently than MMAE (0.47–6.5 nM), removal of the Cbz‐group yielded dramatically increased IC_50_‐values (9.8–170 nM). We attribute the reduced *apparent* cytotoxicity of the deprotected azastatin methyl esters to a lack of membrane permeability. These results clearly establish the azastatins as a novel class of cytotoxic payloads ideally suited for use in next‐generation ADC development.

## Graphical abstract

**Another golden bullet**: Antibody–drug conjugates (ADCs) are becoming increasingly common in clinical chemotherapy. Unfortunately, some of the cytotoxic drugs used to arm antibodies can leak into surrounding tissues after being delivered to the tumour and cause “bystander killing”. Herein, we report the design, synthesis, and evaluation of azastatin, a novel, more “bystander‐friendly” microtubule inhibitor than those preceding it.

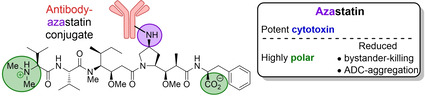

## Introduction

**Figure 1:**
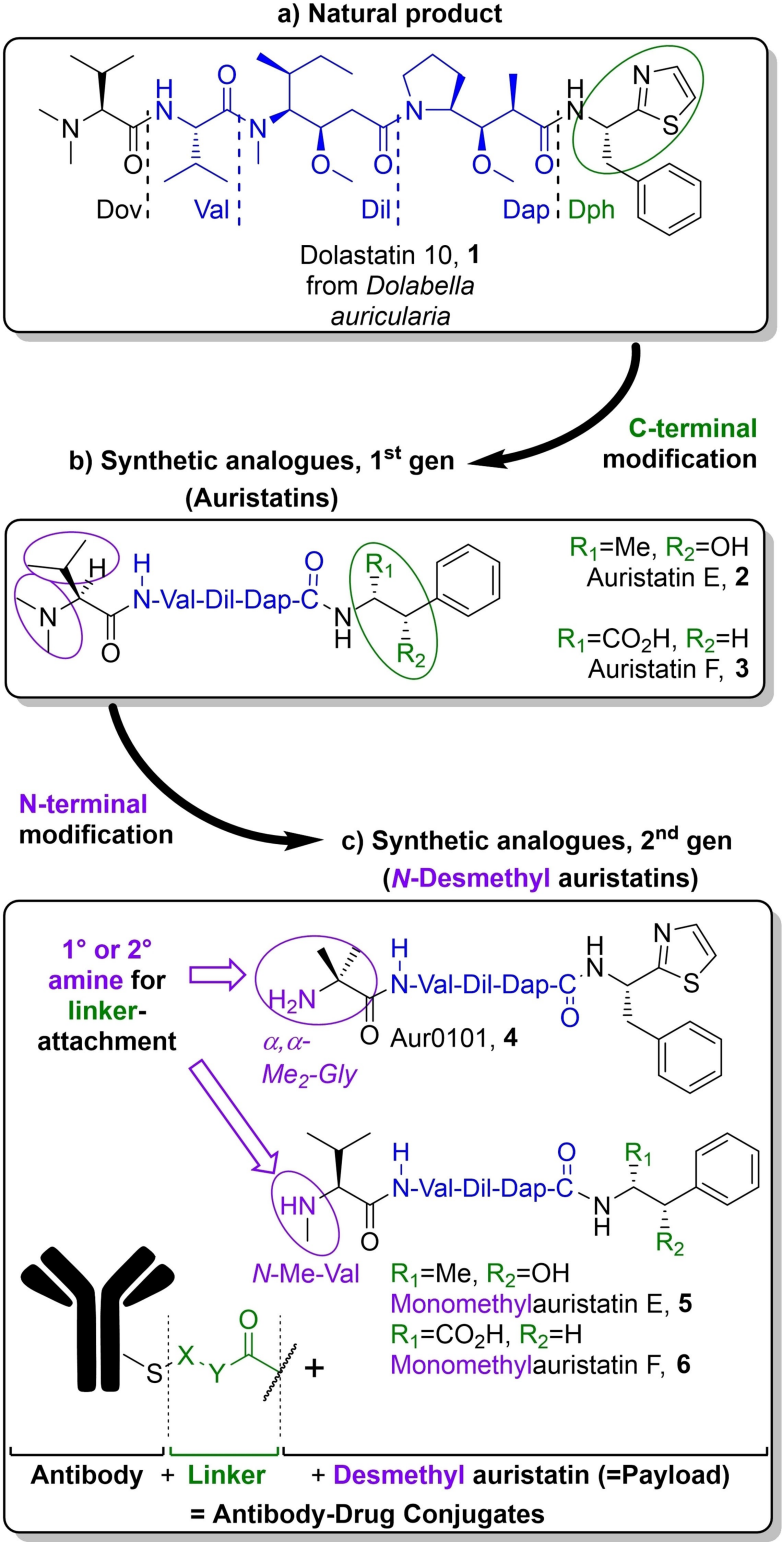
The structural evolution of auristatins. While dolastatin 10, their natural predecessor, has failed to gain clinical relevance, three antibody–drug conjugates armed with a second generation auristatin have received marketing authorization and are used in cancer treatment.

Dolastatin 10 is a natural product first isolated by Pettit et al from the marine mollusc *Dolabella auricularia* in 1987 (see Figure 1). Despite the discovery of even more potent inhibitors of cell division since,[^1^, ^2^] the pentapeptide remains among the most potent antineoplastic agents known to man,^[3]^ owing to its inhibition of tubulin polymerisation and GTP hydrolysis.^[4]^ Unfortunately, clinical development soon revealed that the compound could cause granulocytopenia,[^5^, ^6^] which prohibited its administration at doses sufficient to treat breast,^[7]^ lung,^[8]^ and prostate cancers.^[9]^

Structural modification by the Pettit^[10]^ and Miyazaki^[11]^ groups yielded the first generation of synthetic analogues termed auristatins. The second generation of analogues is characterized by the replacement of the N‐terminal dimethylamino group with a nucleophilic secondary[^11^, ^12^, ^13^] or primary amine.^[14]^ These synthetic handles have been widely exploited to conjugate the cytotoxins to tumour‐specific antibodies in order to mitigate toxicity and widen the therapeutic window substantially. Today, three antibody–drug conjugates (ADCs) derived from MMAE (monomethylauristatin E, **5**) have received marketing authorization.[^15^, ^16^, ^17^, ^18^] Together with ADCs encompassing MMAF (monomethylauristatin F, **6**), the two payloads are represented in at least a dozen clinical studies,[^19^, ^20^] while Pfizer's development of an ADC armed with Aur0101 (**4**) ended in Phase I due to its severely limited therapeutic window.^[21]^

Despite the success of some antibody‐auristatin conjugates, none of the payloads are without flaw. Due to its pronounced cellular permeability, MMAE (**5**) is able to diffuse out of the neoplastic target cell upon antibody detachment and spread into neighbouring tissues in a process called “bystander killing”. While this behaviour may in some instances be desirable to target antigen‐negative cancer cells and can deliver cytotoxicity to non‐vascularised sections of solid tumours, it can also cause harm to healthy tissues.[^22^, ^23^] MMAF (**6**), which is zwitterionic and therefore less permeable upon release, is harmless for bystander cells by comparison.[^22^, ^24^, ^25^, ^26^]

The differences between **5** and **6** highlight a difficulty frequently encountered when comparing non‐conjugated ADC‐payloads *in vitro*: In the absence of an antibody capable of transporting the small molecules across the lipid membrane, impermeable payloads, such as **6**, appear substantially less cytotoxic than their permeable congeners. As proven by the comparable clinical utility of ADCs derived from either **5** or **6**, this difference is largely due to differences in permeability,^[26]^ as opposed to the payloads’ abilities to inhibit their cellular target. To accommodate this discrepancy, we therefore propose the use of the terms *apparent cytotoxicity* and *inherent potency*. An inherently potent tubulin‐inhibitor such as **5** will only *appear* cytotoxic if it can reach its target; if it cannot, as in the case of **6**, its IC_50_‐value is uninformative and the concept of inherent potency is more useful to predict its utility as an ADC payload. In fact, a warhead incapable of causing bystander‐killing must be impermeable and therefore display a low degree of *apparent* cytotoxicity while retaining high *inherent* potency.

Moreover, the deficiencies associated with the use of auristatins as ADC‐payloads are not confined to bystander‐killing: Conjugation of a relatively hydrophobic auristatin to a hydrophilic antibody can cause the protein to aggregate, diminishing its biological half‐life and thereby therapeutic utility.^[27]^ When taking into account other, even more hydrophobic ADC‐payloads of other classes,^[28]^ MMAE's tendency to cause ADC‐aggregation is likely linked to its relative hydrophobicity and thus goes hand‐in‐hand with its propensity to cause bystander killing. This hypothesis also explains the striking differences between MMAE and MMAF on both counts. Lastly, MMAE has been found to be slightly less cytotoxic *in vitro* than its auristatin analogue,^[29]^ highlighting the importance of the N‐terminal dimethylamino‐moiety as found in dolastatin 10 for inherent potency.

The majority of SAR‐studies have examined the effects of variations of the N‐ and C‐terminal amino acids.^[26]^ Out of the five amino acids, the least SAR‐data is available for modifications of the Dap residue, presumably due to the considerable synthetic effort required to generate analogues thereof. At the onset of our study, there were no literature examples exploring Dap‐SAR and since, only one literature account has been published.^[30]^

**Figure 2:**
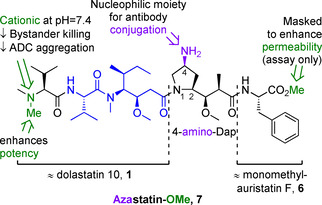
Azastatin‐OMe (**7**), the target molecule of the work outlined herein. The Val‐Dil core common to dolastatin **10** and its derivatives is highlighted in blue, two key structural elements at the N‐ and C‐termini are marked green. The additional amino moiety intended for antibody conjugation is drawn in purple.

Compound **7**, which we have named azastatin methyl ester (azastatin‐OMe, see Figure 2), was designed to ameliorate the pharmacokinetic and physicochemical issues associated with auristatins. Notably, the target structure contains a primary amine in the (4‐pyrrolidine)‐position of the Dap residue, offering an additional diversification point and a synthetic handle for bioconjugation and subsequent ADC synthesis. Furthermore, retention of the important N‐terminal Dov binding motif should not only enhance potency relative to monomethylauristatins but also substantially reduce ADC hydrophobicity and aggregation.

Lastly, to imitate the relatively low permeability associated with MMAF, its C‐terminal phenylalanine residue was accommodated in the target molecule. The C‐terminal carboxylic acid was masked as its corresponding methyl ester to enable *in vitro* cytotoxicity evaluation without the need for prior antibody conjugation. As the C‐terminal methyl ester of MMAF (**6**‐OMe) has been found to display a pronounced *apparent cytotoxicity* and is frequently used as a benchmark in auristatin‐SAR,[^14^, ^26^] we hypothesized that this analogous modification would not distort the *inherent potency* of azastatin.

In this work, we describe the synthesis of azastatin‐OMe (**7**) and two of its congeners as potential ADC‐payloads. Their apparent cytotoxicity towards three tumour cell lines was subsequently assessed and the differences were used to extrapolate the hypothetical, inherent potency of the azastatin core to gain insight into its potential utility in ADC research.

## Results and Discussion

### Retrosynthetic analysis

**Figure 3:**
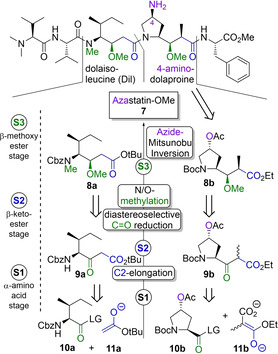
Retrosynthetic analysis of the target molecule. Both of the key γ‐amino acids dolaisoleucine and 4‐aminodolaproine were to be obtained from their respective α‐amino acid homologues (stage 1) in an analogous fashion: C2‐elongation was to yield β‐keto esters (stage 2), which were to be reduced diastereoselectively to furnish highly chiral β‐methoxy esters (stage 3).

While three of the five amino acids encompassing the target molecule were commercially available (L‐valine, L‐phenylalanine) or could be generated from a commercial source in a trivial fashion (*N*,*N*‐dimethyl‐L‐valine), the two central γ‐amino acids (dolaisoleucine, dolaproine) deserved retrosynthetic attention (see Figure 3). In keeping with their structural similarities, we envisioned a forward synthesis starting from analogous α‐amino acid esters (**10 a** and **10 b**). The stage 1 substrates derived from L‐isoleucine and hydroxyproline, respectively, were to be C2‐elongated to β‐keto‐γ‐amino acid esters (**9 a** and **9 b**) by reaction with enolates derived from either *tert*‐butyl acetate (**11 a**) or 2‐methyl malonate **11 b**. These stage 2 ketones were hypothesized to lend themselves to diastereoselective carbonyl reduction followed by N/O‐methylation to yield the desired stage 3 analogues, i. e. β‐methoxy‐γ‐amino acid esters **8 a** and **8 b**.

### Synthesis of N‐terminal tripeptide

**Scheme 1:**
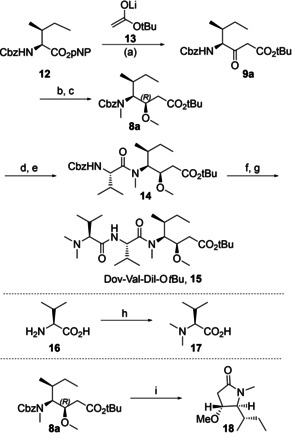
Synthesis of Dov‐Val‐Dil‐OtBu, **15**. Reagents and conditions: a) *t*BuOAc, HN*i*Pr_2_, *n*BuLi, THF, 84 %. b) NaBH_4_, SiO_2_, DCM/*i*PrOH 15 : 1, 83 % (5 : 1 mixture of diastereoisomers). c) MeOTf, LiHMDS, DMEU, THF, 60 %. d) 1. Cyclohexene, Pd/C, MeOH. 2. HCl/Dioxane, 90 %. e) Cbz‐Val‐OH, BEP, NEt*i*Pr_2_, DCM, 85 %. f) H_2_, Pd/C, MeOH. g) **17**, DECP, NEt_3_, DCM, 54 % over two steps. h) CH_2_O, H_2_, Pd/C, H_2_O/MeOH, 99 %. i) H_2_, Pd/C, EtOAc/MeOH 3 : 1, 53 %.

Reaction of commercially obtained *para*‐nitrophenyl ester **12** with *tert*‐butyl acetate lithium enolate **13** resulted in β‐keto ester **9 a** in 84 % yield (see Scheme 1). Sodium borohydride mediated reduction in DCM/iPrOH (15 : 1) then gave a mixture of alcohols in a ratio of 5 : 1 in favour of the desired (3*R*)‐diastereomer (83 % yield of diastereomer mixture). In our hands, the use of this solvent mixture was preferable to a previously published method using methanol,[^11^, ^31^] in which we found the diastereoselectivity to be negatively impacted by scale‐up. A method involving the use of lithium borohydride in THF has also been published.^[32]^

The diastereomeric mixture was subsequently dimethylated using methyl triflate to yield *N*,*O*‐dimethyl intermediate **8 a**,^[31]^ which could be separated from the (3*S*)‐diastereomer by flash chromatography (60 % yield of pure diastereomer). In order to assess the relative configuration of the newly formed methoxy‐substituted stereocenter in **8 a**, it was cyclized to give lactam **18**. The pyrrolidinone was spectroscopically identical to previous literature reports.[^11^, ^33^, ^34^] The Cbz‐group was removed by cyclohexene‐mediated transfer hydrogenation, followed by precipitation of the free amine from ethereal HCl to obtain dolaisoleucine hydrochloride in 90 % yield.^[34]^

2‐Bromo‐1‐ethylpyridinium tetrafluoroborate (BEP)^[35]^ was used to couple the secondary amine to Cbz‐L‐valine to give dipeptide **14** (85 % yield).^[36]^ Compound **14** was deprotected by means of hydrogenation while dolavaline (**17**) was furnished by reductive amination of aqueous formaldehyde with L‐valine (**17**, 99 % yield).^[37]^ Finally, the two fragments were coupled to yield Dov‐Val‐Dil‐O*t*Bu (**15**) in 54 % yield.^[34]^

### Synthesis of C‐terminal dipeptide

**Scheme 2:**
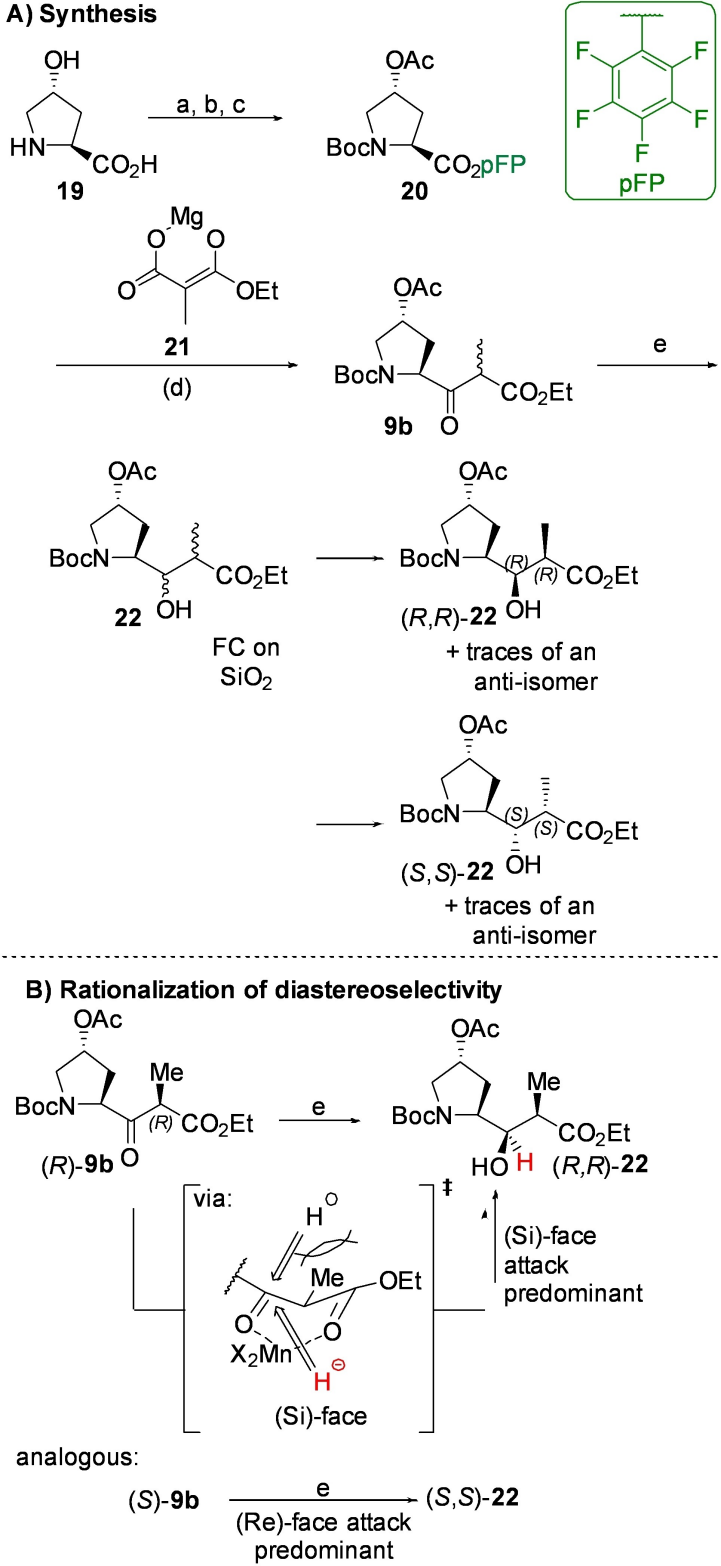
**A)** Synthesis of key intermediate **22. B)** Rationalization of the syn‐selectivity of the reduction of **9 b** by means of the Cram chelate model. Reagents and conditions: a) Boc_2_O, NaOH, H_2_O/THF. b) Ac_2_O, pyridine, DCM. c) C_6_F_5_OH, DCC, EtOAc, 60 % over three steps. d) EtO_2_C‐CH(Me)‐CO_2_H, *i*PrMgBr, THF, 72 % (15 % of unreacted **20**). e) NaBH_4_, MnCl_2_, MeOH, 95 %. 35 % of (*R*,*R*)‐**22** upon purification by flash chromatography on silica.

To enable C2‐elongation, pentafluorophenyl ester **20** (see Scheme 2A) was prepared from trans‐4‐hydroxy‐L‐proline **19** by Boc‐protection, acetylation and DDC‐mediated ester formation in 60 % yield over three steps. Electrophile **20** was subsequently treated with magnesium complex **21** to yield **9 b** as a 1 : 1 mixture of epimers (72 % yield, 15 % of unreacted **20**). The synthetic sequence toward ketone **9 b** is based on Genet and coworkers’ use of carbonyldiimidazol to activate Boc‐*N*‐proline *in situ*, followed by an analogous quench with reagent **21**, which yielded desacetoxy‐**9 b** in 82 % yield.^[38]^

In the presence of manganese dichloride, the subsequent sodium borohydride mediated reduction of the β‐oxoester **9 b** yielded a mixture of diastereoisomers of **22** in an approximate ratio of 9 : 1 : 9 : 1. Pleasingly, the major diastereomers could be separated by flash chromatography.

**Scheme 3:**
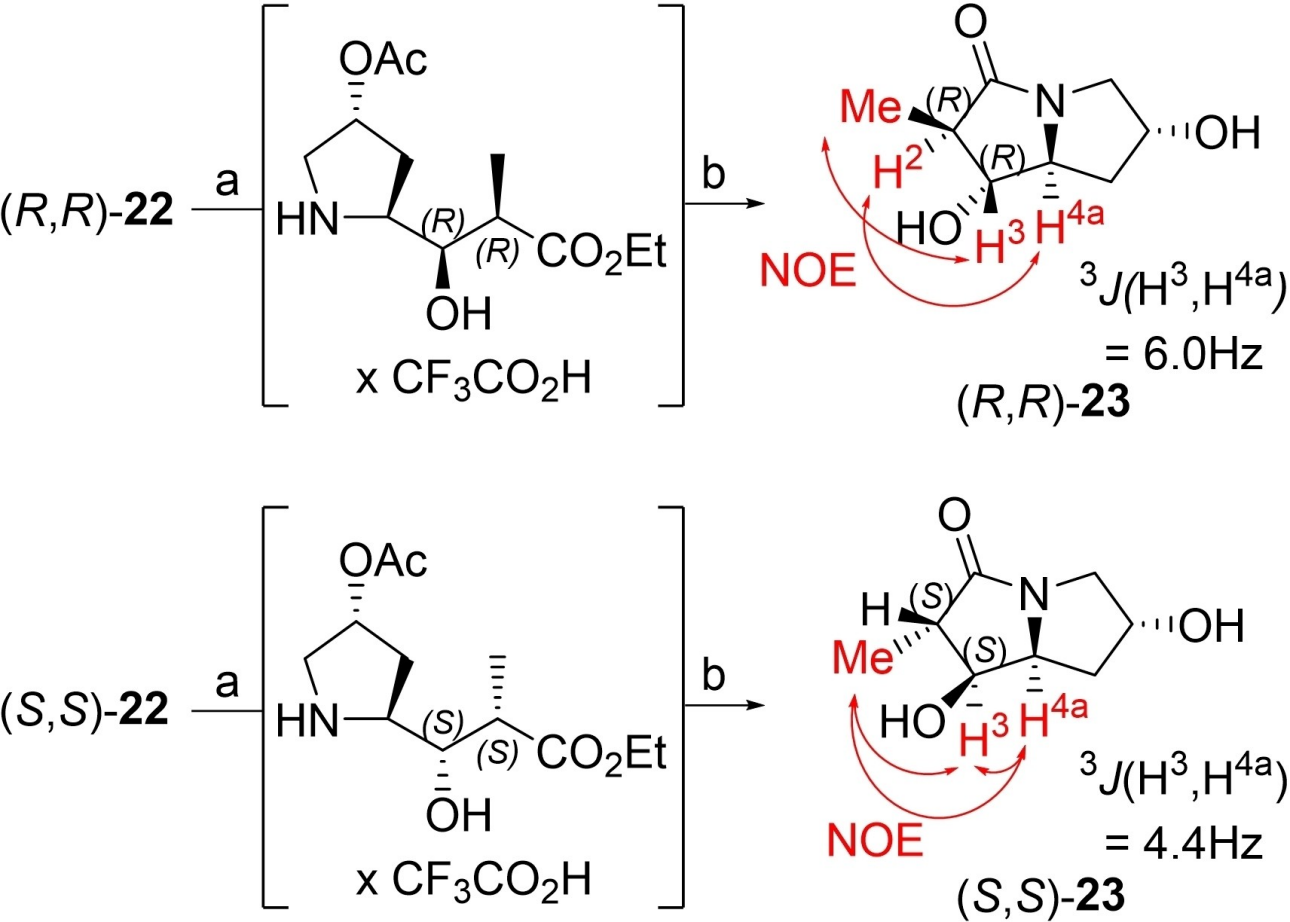
Stereochemical characterization of both major diastereomers of **22** by means of ^1^H‐NMR and ^1^H‐^1^H nuclear Overhauser effect (NOE) spectroscopy. Selected spacial interactions are symbolized by red arrows. Reagents and conditions: a) TFA/DCM 1 : 1. b) Cs_2_CO_3_, MeOH.

Numerous authors have demonstrated the reliability of cyclisation of desacetoxy‐**22** when faced with the task to assign the relative conformation of the stereocenters α and β to the ester carbonyl group.[^38^, ^39^, ^40^] Reflective of this approach, each diastereomerically enriched fraction of **22** was treated with trifluoroacetic acid to cause Boc‐cleavage and upon basification, the corresponding dihydroxy‐γ‐lactams (**23**) were obtained (see Scheme 3). Nuclear Overhauser effect (NOE) spectroscopy of one of the lactams revealed the spacial proximity of the α‐carbonyl hydrogen atom (H^2^) with that bound to the bridgehead (H^4a^), indicating that the α‐carbon was in the (*R*)‐configuration. The methyl group bound at the same carbon showed an NOE‐interaction with the proton vicinal to the OH‐group in the pyrrolidine‐ring (H^3^), suggesting that this stereocenter was also in the (*R*)‐configuration. Based on this data, the parent γ‐amino acid was assigned to be the desired (*R*,*R*)‐diastereomer of **22**.

An analogous transformation followed by NOE spectroscopic analysis of the other major reduction product revealed the spacial proximity of the methyl group, H^3^, and H^4a^, suggesting that the lactam was (*S*,*S*)‐configured. On the basis of these findings, the parent γ‐amino acid was concluded to be (*S*,*S*)‐**22**.

The marked syn‐selectivity of the reduction (i. e. preference for the formation of (*R*,*R*)‐ and (*S*,*S*)‐**22**) has been described in literature,^[41]^ and can be rationalized by applying a modification of the Cram chelate model (see Scheme 2B).^[42]^ Based on this reasoning, it appears likely that the predominant factor determining the stereochemical outcome of the reduction is the configuration of the methyl‐substituted α‐carbon, while the two stereocenters on the pyrrolidine ring do not exert a substantial degree of stereochemical induction. This behaviour markedly distinguishes ketones **9 a** and **9 b** (see Figure 3), which can be considered functionally equivalent in light of their analogous roles as prochiral stage 2 intermediates.

**Scheme 4:**
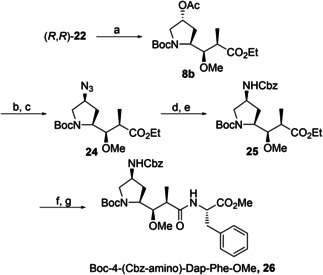
Synthesis of C‐terminal dipeptide 26. Reagents and conditions: a) NaH, Me_2_SO_4_, THF/DMF 3 : 1, 80 %. b) K_2_CO_3_, MeOH/H_2_O 40 : 1, 97 %. c) DIAD, DPPA, PPh_3_, THF, 85 %. d) H_2_, Pd/C, EtOAc. e) CbzCl, NEt_3_, DCM. f) LiOH, MeOH/H_2_O, 1 : 1. g) H_2_N‐Phe‐OMe x HCl, HATU, NEt*i*Pr_2_, DCM, 58 % over four steps.

The target diastereomer (*R*,*R*)‐**22** was methylated using dimethyl sulfate and sodium hydride to yield **8 b** in 80 % yield (see Scheme 4). The acetyl protecting group was selectively removed by basic hydrolysis in aqueous methanol in 97 % yield, followed by azide‐Mitsunobu inversion to yield **24** (85 % yield). Reduction of the newly introduced azide was accomplished by hydrogenation, followed by Cbz‐protection of the resulting amine to yield intermediate **25**. Cleavage of the ethyl ester without epimerization proved difficult but was achieved by hydrolysis in a mixture of excess lithium hydroxide in aqueous methanol at 0 °C for two days. Finally, HATU‐mediated amide coupling with commercially obtained L‐phenylalanine methyl ester furnished Boc‐4‐(Cbz‐amino)‐Dap‐Phe‐OMe (**26**) in 58 % yield over four steps.

### Endgame peptide assembly

**Scheme 5:**
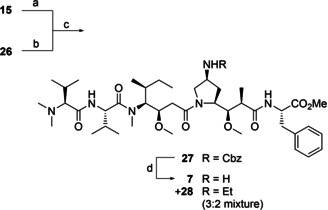
Ligation of the N‐terminal tripeptide **15** and C‐terminal dipeptide **26** to yield Cbz‐Azastatin‐OMe, **27**. The final deprotection yielded both Azastatin‐OMe, **7** and its *N*‐ethyl analogue, **28**. Reactions and conditions: a) DCM/TFA 1 : 1. b) DCM/TFA 8 : 3. c) DECP, NEt_3_, DME, 75 % over three steps. d) H_2_, Pd/C, EtOH, 98 % (mixture of **7** and **28**).

*tert*‐Butyl deprotection of **15** and Boc‐deprotection of **26** were accomplished by treatment with trifluoroacetic acid in dichloromethane (see Scheme 5). DEPC‐mediated amide coupling^[25]^ yielded Cbz‐protected pentapeptide **27**. While hydrogenation in ethyl acetate failed to remove the Cbz‐group, the same reaction in methanol only yielded mono‐ and dimethylated products as opposed to the desired azastatin‐OMe (**7**). On a test scale, hydrogenation in ethanol yielded **7** seemingly quantitatively, but when the reaction was scaled up, a 3 : 2 mixture of **7** and its *N*‐ethyl analogue **28** was obtained in a combined yield of 98 %. Pleasingly, separation of **7** and **28** was accomplished by flash chromatography.

### Biological evaluation

Three fundamentally different cell lines were chosen for the assessment of cytotoxicity: HepG2 cells, which derive from a human hepatoblastoma;^[43]^ HCT116 cells, a common model for the study of metastatic colorectal carcinoma;[^44^, ^45^] and RPMI 8226 cells, which originate from multiple myeloma.^[46]^

The cells, upon overnight cultivation, were treated with MMAE (**5**), MMAF (**6**), azastatin‐OMe (**7**), *N*‐ethyl azastatin‐OMe (**28**), *N*‐Cbz azastatin‐OMe (**27**) or doxorubicin[^47^, ^48^] at concentrations ranging from 100 μM to 10 pM and after 72 hours of incubation, the survival index relative to control was determined by The Fluorometric Microculture Cytotoxicity Assay (FMCA).^[49]^ Based upon the concentration dependent proportion of surviving cells, the half maximal inhibitory concentration (IC_50_) of each compound was calculated (see Table 1).

**Table 1:** Structures and IC_50_‐values of three new auristatins (**7, 28, 27**) compared to well‐established monomethyl‐auristatins and doxorubicin in three cancer cell lines. Image: Predominant charge states of all five auristatins at physiological pH.

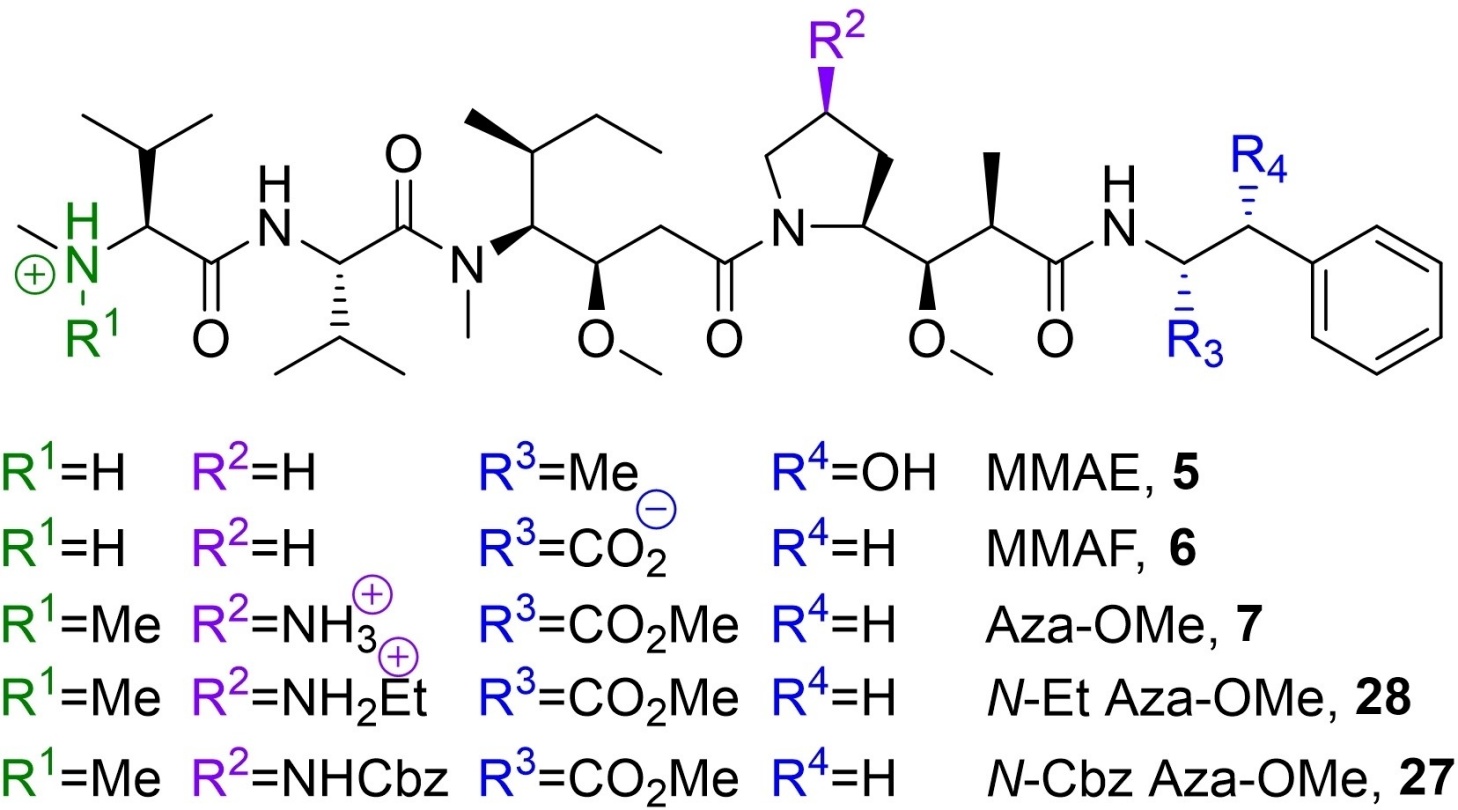			
	IC_50_^[a]^		
Compound	HepG2	HCT116	RPMI 8226
MMAE, **5**	0.52 (0.42‐0.65)	6.5 (5.4–7.8)	0.47 (0.43–0.52)
MMAF, **6**	130 (110–150)	2700 (2100–3300)	200 (170–240)
Aza‐OMe, **7**	20 (16–25)	170 (140–200)	9.8 (8.0–12)
*N*‐Et Aza‐OMe, **28**	11 (8–13)	100 (84–120)	4.8 (4.2‐5.5)
Cbz‐Aza‐OMe, **27**	0.13 (0.11–0.16)	3.0 (2.1‐4.3)	0.15 (0.13–0.17)
Doxorubicin	400 (320–520)	440 (410–470)	49 (40–59)

[a]Mean IC_50_‐values and 95 % confidence intervals [nM] based on three independent experiments (see SI).

MMAE (**5**) was found to be active in all three cell lines but proved to be an order of magnitude less potent in HCT116 cells (IC_50_=6.5 nM) than in HepG2 (IC_50_=0.52 nM) and RPMI 8226 cells (IC_50_=0.47 nM). The same trend was observed for MMAF (**6**), which was more than 100‐fold less potent in each of the cell lines (IC_50,HCT116_=2.7 μM, IC_50,HepG2_=130 nM, IC_50,RPMI‐8226_=200 nM). These findings are in line with literature reports and suggest that the decreased potency of MMAF (**6**) can be attributed to its decreased cellular permeability as opposed to a lower inherent potency, i. e. a diminished ability to inhibit the function of its target protein.[^22^, ^24^, ^25^, ^26^] Strikingly, in all but one cell line, MMAF (**6**) appeared less cytotoxic than even doxorubicin (IC_50,HCT116_=440 nM, IC_50,RPMI‐8226_=32 nM), which is generally considered insufficiently potent to be an ADC‐payload.[^50^, ^51^] The only cell line in which MMAF (**6**) was observed to be marginally more active than doxorubicin (IC_50_=400 nM) was HepG2.

Like MMAE (**5**) and MMAF (**6**), *N*‐Cbz azastatin‐OMe (**27**) more potently inhibited cell division in HepG2 (IC_50_=0.13 nM) and RPMI 8226 (IC_50_=0.15 nM) than in HCT116 cells (IC_50_=3.0 nM), but exceeded even MMAE (**5**) in cytotoxicity by a factor of more than two in all cell lines. In agreement with the biological data published for other auristatins, these findings suggest that the N‐terminal dimethylamino group increases potency,[^26^, ^29^] and that nitrogen‐substitution at the 4‐position of dolaproine does not diminish it.^[30]^ The increased potency of *N*‐Cbz azastatin‐OMe (**27**) relative to MMAE (**5**) and MMAF (**6**) is in stark contrast to azastatin‐OMe (**7**, IC_50,HepG2_=20 nM, IC_50,HCT116_=170 nM, IC_50,RPMI‐8226_=9.8 nM) and *N*‐ethyl azastatin‐OMe (**28**, IC_50,HepG2_=11 nM, IC_50,HCT116_=100 nM, IC_50,RPMI‐8226_=4.8 nM), which both appeared substantially less cytotoxic in all cell lines than MMAE (**5**). Both compounds are expected to be predominantly dicationic at physiological pH, which likely diminishes their cellular permeability. It may therefore be hypothesized that, like the zwitterionic MMAF (**6**), both azastatin‐OMe (**7**) and *N*‐ethyl azastatin‐OMe (**28**) are characterized by a high degree of *inherent* potency, which differs from their *apparent* cytotoxicity only due to their limited permeability. This interpretation of the data is in agreement with the observation that the structurally similar, monocationic *N*‐Cbz azastatin‐OMe (**27**), to which the cellular membrane poses much less of an obstacle on its way to the target protein, displays exquisite cytotoxicity. Lastly, it appears noteworthy to point out that the slightly less polar *N*‐ethyl azastatin‐OMe (**28**) displays a marginally superior cytotoxicity compared to azastatin‐OMe (**7**). Assuming that the decreased hydrophilicity of *N*‐ethyl azastatin‐OMe (**28**) accounts for a somewhat increased propensity to penetrate the cell membrane relative to azastatin‐OMe, this lends additional credibility to the hypothesis of permeability‐limited potency.

## Conclusion

Three novel auristatins were synthesized with the goal of capturing the inherent potency of dolastatin 10 (**1**) and decreased efflux permeability of monomethyl auristatin F (**6**). Based on this rationale, the target molecules were designed to encompass dolastatin's N‐terminal dolavaline, an additional amino‐moiety at the pyrrolidine core, and MMAF's C‐terminal phenylalanine residue masked with a methyl group. The synthesis of the two central γ‐amino acids of the pentapeptides was achieved by a chiral‐pool approach starting from isoleucine and hydroxyproline, respectively.

In addition to the novel payloads, the well‐characterized, structurally similar cytotoxins MMAE (**5**) and MMAF (**6**) were tested for their tumoricidal effects. By confirming the stark differences in the IC_50_‐values of the permeable MMAE (0.47–6.5 nM) and impermeable MMAF (130 nM−2.7 μM) also reported elsewhere,^[26]^ this work serves as additional evidence in favour of the hypothesis of permeability‐controlled apparent cytotoxicity of auristatins. In the context of antibody‐mediated drug delivery, intrinsic membrane permeability of the payload is however not required for it to reach antigen‐positive target cells.^[23]^ As we have argued, one may in fact want to suppress intrinsic permeability so as to mitigate bystander‐killing, the process by which a cytotoxin diffuses out of the target cell to which it was delivered and harms neighbouring tissues indiscriminately. We have therefore chosen to differentiate between an analyte's apparent cytotoxicity (i. e. the antineoplastic activity of payload as measured *in vitro)* and inherent potency (i. e. the effective, not directly measurable ability of a payload to cause apoptosis once released inside the cancer cell).

Cbz‐protected azastatin methyl ester (**27**), whose cellular influx was not stalled by the presence of a charged ammonium residue, displayed exquisite potency in all tested cell lines (0.13–3.0 nM). The model peptide's cytostatic prowess is illustrative of the activity enhancement associated with dolavaline incorporation and indicative that 4‐pyrrolidine substitution does not diminish inherent potency. On the other hand, azastatin methyl ester (**7**) and its *N*‐ethyl analogue (**28**), both of which are less membrane‐permeable than either MMAE (**5**) or their Cbz‐protected congener, exhibited a substantially reduced apparent cytotoxicity in all cell lines (9.8–170 nM and 4.8–100 nM, respectively).

Most forms of cancer remain incurable. While antibody–drug conjugates represent a cutting‐edge approach to extending patients’ lifespans, they have yet to find their way into first‐line treatment. The biological data portrayed herein suggest that azastatins, which represent a novel class of payload for antibody conjugation, may be ideally suited for the job. The example of Cbz‐azastatin methyl ester (**27**) serves to highlight their inherent potency while the substantially decreased cytotoxicity of non‐protected azastatins (**7** and **28**, respectively) can be extrapolated to indicate minimal bystander killing in the ADC‐context. We hope that the altered physicochemical properties of azastatins may yield ADCs with a wider therapeutic window and ultimately constitute a steppingstone towards propelling targeted therapeutics into first‐line cancer treatment.

## Experimental Section

### Synthesis and purification: General

All solvents used in the synthesis and purification of the compounds described herein were purchased from Chemtronica (Sweden) and used without further purification. Solvents used in non‐aqueous reactions were stored over molecular sieves (3 Å or 4 Å, Sigma‐Aldrich/Merck, Germany) prior to use. Cbz‐L–Isoleucine *para*‐nitrophenyl ester was obtained from abcr (Germany). All other starting materials and reagents were purchased from Sigma‐Aldrich/Merck (Germany). Non‐aqueous reactions were carried out in an atmosphere of nitrogen, which was pre‐dried using a Drierite gas drying unit (W.A. Hammond Drierite Company, US). Unless otherwise noted, reactions were monitored using an Agilent (US) 1100 series LC/MS (single quadrupole) system equipped with an electrospray interface, a UV diode array detector and an ACE3 C8 (3.0×50 mm) column (ACE, UK) with a gradient of acetonitrile (10→97 %) in 0.1 % aqueous trifluoracetic acid over 3 min and a flow of 1 mL/min or an XBridge C18 (3.0×50 mm) column (Waters, US) with a gradient of acetonitrile (5→97 %) in 10 mM aqueous ammonium bicarbonate. When analytes were too polar to allow for facile reaction monitoring by reverse phase chromatography, TLC on aluminium supported silica plates (Merck, Germany) was used to monitor reactions, instead. Flash chromatography was performed either manually using 40–63 μm silica (Carlo Erba, Italy) or automatically using a CombiFlash Rf^+^ Lumen flash machine (Teledyne Isco, US) equipped with a wide‐range UV and evaporative light scattering (ELS) detector and prepacked silica columns (SiliCycle, Canada).

### Analytical characterisation: General

NMR spectra were recorded using an Ascend 400 spectrometer (Bruker, US) at 298 K. ^1^H‐spectra were recorded at 400 MHz, ^19^F‐spectra at 376 MHz and ^13^C‐spectra at 101 MHz. All NMR‐experiments were performed using commercially obtained, deuterated solvents with no further purification (Sigma‐Aldrich/Merck, Germany). DMSO‐d_6_ was stored over molecular sieves (4 Å, Sigma‐Aldrich/Merck, Germany). NMR spectra were processed and interpreted using MestreNova (Mestrelab, Spain). High‐resolution mass spectra were acquired using a Premiere Q‐TOF mass spectrometer (Waters, US) operating in ES+ mode. The analytes were introduced into the mass spectrometer after chromatography on an Acquity UPLC system equipped with an Acquity UPLC BEH C18 (2.1×100 mm) column (both Waters, US), running a gradient of 0.1 % formic acid in acetonitrile (5→95 %) in 0.1 % aqueous formic acid over 6 min. Mass spectra and chromatograms were processed using MassLynx and UNIFI computer software (Waters, US). Optical rotation measurements were performed using an Autopol II S2 polarimeter equipped with 2.0 mL cuvette (both Rudolph Research Analytical, US) with pathlength l=1 dm.

### *N*Cbz‐β‐keto‐γ‐amino acid *tert*‐butyl ester 9a

A three‐necked, 250 mL round‐bottom flask equipped with a nitrogen inlet, thermometer and stirring bar was charged with di*iso*propylamine (9.0 mL, 64.2 mmol, 3.1 eq.) and THF (35 mL) and the resulting solution was cooled to −78 °C. Dropwise, over the period of 15 minutes, n‐butyllithium (2.5 M solution in hexanes, 25 mL, 62.5 mmol, 3.0 eq.) was added while the temperature inside the reaction mixture was kept below −45 °C. After 30 minutes of stirring, *tert*‐butyl acetate (8.4 mL, 62.6 mmol, 3.0 eq.) was added dropwise over the period of 10 minutes while the temperature inside the flask was kept below −55 °C. The reaction mixture was stirred for one hour on dry ice. Over the period of one hour, a solution of **12** (8.0 g, 20.7 mmol, 1.0 eq.) in THF (34 mL) was added dropwise, while the temperature inside the flask was kept below −55 °C. After two hours, the reaction mixture was allowed to warm to 0 °C and the remaining enolate was quenched by the addition of saturated aqueous NH_4_Cl (10 mL, temperature below 5 °C). The mixture was allowed to attain room temperature and was diluted with water (100 mL) and EtOAc (100 mL). The phases were separated, and the aqueous phase was extracted with EtOAc (3×100 mL). The combined organic layers were washed with half‐saturated brine (100 mL), dried over MgSO_4_, filtered and the solvent was removed under reduced to yield a yellow syrup. The latter was taken up in DCM (200 mL), silica (50 g) was added and the solvent was gently removed to yield a dry, yellow powder. The latter was washed with EtOAc/heptane (1 : 1, 400 mL) and the solvent was removed *in vacuo* to yield a yellow oil, which was purified by flash chromatography on silica (petroleum ether/EtOAc 9 : 1→3 : 1) to yield **9 a** as a pale yellow oil (6.3 g, 17.4 mmol, 84 % yield). Another method of preparation makes use of CDI and Cbz‐L‐isoleucine.^[31]^ **MS** (ESI) m/z (%): 386 (18) [*M*+Na]^+^, 308 (39) [*M*‐*t*Bu+H]^+^, 264 (100) [*M‐*CO_2_ *t*Bu+H]^+^. ^**1**^ **H NMR** (400 MHz, DMSO‐d_6_), mixture of rotamers: δ=7.72 and 7.67 (d, *J*=8.4 Hz and d, *J*=9.0 Hz, 1H), 7.41–7.28 (m, 6H), 5.07 and 5.05 (s, 2H), 4.01 and 3.87 (dd, *J*=8.6, 6.6 Hz and d, *J*=8.5 Hz, 6.1 Hz, 1H), 3.69–3.59 and 3.51 (m and d, *J*=16.2 Hz, 1H), 3.45 (d, *J*=16.2 Hz, 1H), 1.91–1.82 (m, 1H), 1.45 and 1.38 (s and s, 9H), 1.33–1.25 (m, 1H), 1.17–1.08 (m, 1H), 0.84 (d, *J*=6.8 Hz, 3H), 0.80 (t, *J*=7.4 Hz, 3H). ^**13**^ **C NMR** (101 MHz, DMSO‐d_6_) δ 203.3, 166.2, 156.4, 136.9, 128.4, 127.9, 127.8, 80.8, 65.7, 64.7, 47.7, 39.5, 34.7, 27.6, 24.1, 15.5, 11.1.

### *N*Cbz‐Dolaisoleucine *tert*‐butyl ester 8 a

A 500 mL round‐bottom flask equipped with a stir bar was charged with **9 a** (19.0 g, 52.3 mmol, 1.0 eq.), which was dissolved in a mixture of isopropanol and DCM (1 : 15, 240 mL). Silica was added (37.7 g, 627.5 mmol, 12.0 eq.) and the resulting suspension was cooled on ice. Sodium borohydride (6.0 g, 158.6 mmol, 3.0 eq.) was added incrementally over the period of one hour. The suspension was stirred on ice for four hours. The remaining borohydride was quenched by the addition of glacial acetic acid (20 mL) and the mixture was allowed to attain room temperature. Once gas evolution had seized, the reaction mixture was filtered, and the solids were washed with EtOAc (300 mL). The solvent was removed under reduced pressure and the resulting oil was co‐evaporated with diethyl ether several times and was subsequently purified by flash chromatography on silica (petroleum ether/EtOAc 4 : 1) to yield a mixture of the two diastereoisomers of the Cbz*N*‐β‐hydroxy‐γ‐amino acid *tert*‐butyl esters (15.8 g, 43.2 mmol, 83 % yield) in a ratio of roughly 5 : 1 in favour of the desired (3*R*)‐isomer. The mixture was used directly in the next step without further purification. Another method of preparation makes use of methanol as solvent.^[31]^ **MS** (ESI) m/z (%): 310 (100) [*M*‐*t*Bu+H]^+^.

A three‐necked, 1 L round‐bottom flask equipped with a thermometer, septum, dropping funnel with nitrogen inlet and stirring bar was charged with lithium bis(trimethylsilyl)amide (1 M in THF, 112 mL, 112 mmol, 2.6 eq.). The base was diluted with THF (70 mL), *N*,*N*’‐dimethylethyleneurea (18.7 mL, 173 mmol, 4.0 eq.) was added via syringe and the resulting solution was cooled to −78 °C. Dropwise, over the period of two hours, a solution of a mixture of the 3*R*‐ and 3*S*‐diastereomers of Cbz*N*‐β‐hydroxy‐γ‐amino acid *tert*‐butyl esters (5 : 1 ratio in favour of the 3*R*‐isomer, 15.8 g, 43.2 mmol, 1.0 eq.) in THF (141 mL) was added to the reaction mixture via the dropping funnel. The temperature inside the flask was kept at below −60 °C over the course of the addition. The mixture was stirred at −78 °C for another hour. Dropwise, over the period of 15 minutes, methyl trifluoromethanesulfonate (29.4 mL, 260 mmol, 6.0 eq.) was added to the reaction mixture, which was kept at below −55 °C. The resulting solution was warmed to −47 °C (acetonitrile/dry ice) and after another 30 minutes, to −20 °C (acetone/ice). After stirring for two hours at that temperature, the mixture was cooled on ice and the reaction was quenched by the addition of saturated aqueous NH_4_Cl (50 mL). The two‐phasic mixture was diluted with water (300 mL) and the phases were separated. The aqueous phase was extracted with EtOAc (3×200 mL), the combined organic layers were washed with half‐saturated brine (200 mL) and subsequently dried over MgSO_4_. Upon filtration and evaporation of the solvent, a yellow oil with solid components was obtained. To the latter were added silica (80 g) and DCM (500 mL), the solvent was removed *in vacuo*, and the resulting dispersion was washed with EtOAc/heptane (1 : 1, 500 mL) to yield a yellow oil which still contained solid components. The oil was taken up in EtOAc/heptane (1 : 1, 600 mL) and the solution was washed with water (2×300 mL). The organic layer was then dried over MgSO_4_, filtered and the solvent was removed *in vacuo* to yield a homogenous, yellow oil, which was purified by flash chromatography on silica (petroleum ether/ EtOAc 9 : 1) to yield **8 a** (10.1 g, 25.6 mmol, 60 % yield, 72 % yield with regard to diastereomeric purity of starting material) as a colourless oil. The procedure is based upon a previously published method.^[31]^ HRMS (ESI) m/z [*M*+H]^+^ calcd for C_22_H_36_NO_5_: 394.2588, found: 394.2525. ^**1**^ **H NMR** (400 MHz, CDCl_3_), miture of two rotamers: δ=7.36–7.27 (m, 5H), 5.15 and 5.10 (dd, *J*=12.4, 6.1 Hz and *J*=12.4, 3.0 Hz, 2H), 4.33‐3.94 (m, 1H), 3.90 and 3.84 (ddd, *J*=9.3, 6.2, 3.4 Hz and td, *J*=7.4, 4.0 Hz, 1H), 3.39 and 3.28 (s, 3H), 2.79 and 2.78 (s, 3H), 2.48‐2.29 (m, 2H), 1.80–1.68 (m, 1H), 1.54–1.46 (m, 1H), 1.45 and 1.44 (s, 9H), 1.13–1.00 (m, 1H), 0.97 and 0.92 (d, J=6.7 Hz, 3H), 0.88 and 0.85 (t, J=5.9 Hz, 3H); ^**13**^ **C NMR** (100 MHz, CDCl_3_), mixture of two rotamers: δ=171.5 and 171.3, 157.3 and 157.2, 137.1 and 136.8, 128.5, 128.2 and 128.1, 127.9 and 127.7, 80.9 and 80.8, 78.6 and 78.3, 67.5 and 67.2, 58.1 and 58.0, 38.9 and 38.6, 34.7 and 34.1, 28.2, 26.1 and 26.0, 16.3 and 16.2, 11.4 and 11.3.

### Monocylic lactam 18

A 10 mL round‐bottom flask was charged with **8 a** (40 mg, 102 μmol), Pd/C (10 % w/w, 5 mg) and a stir bar were added, and the flask was sealed with a septum. The atmosphere was evacuated and refilled with nitrogen three times. EtOAc/MeOH (3 : 1, 3 mL) was injected *via* syringe and a hydrogen atmosphere was applied. The mixture was stirred at room temperature for two days. The suspension was passed through a syringe filter, the liquid layer was concentrated *in vacuo*, and the residue was purified by flash chromatography on silica (hexane/acetone 4 : 1) to yield **18** (10 mg, 54 μmol, 53 % yield) as a colourless oil. The procedure is an exact reproduction of the method originally published by Pettit and coworkers.[^11^, ^33^, ^34^] **MS** (ESI) m/z (%): 186 (100) [*M*+H]^+^. ^**1**^ **H NMR** (400 MHz, CDCl_3_) δ=3.67 (ddd, *J*=7.0, 6.8 Hz, 1.7 Hz, 1H), 3.45 (dd, *J*=3.6, 1.7 Hz, 1H), 3.28 (s, 3H), 2.80 (s, 3H), 2.54 (ddd, *J*=17.8, 7.0, 0.9 Hz, 1H), 2.38 (dd, *J*=17.8, 1.1 Hz, 1H), 1.82–1.71 (m, 1H), 1.51–1.40 (m, 1H), 1.35–1.26 (m, 1H), 0.99 (t, *J*=7.4 Hz, 3H), 0.70 (d, *J*=6.9 Hz, 3H). The unusually low trans‐coupling constant between the protons geminal to the methoxy‐ and isobutyl‐groups (3.67 and 3.45 ppm, respectively, ^3^J=1.7 Hz) does not escape us, but corresponds exactly to the value reported by Pettit,^[34]^ whose structural assignment is supported by NOESY.^[33]^

### Dolaisoleucine *tert*‐butyl ester hydrochloride (Dil‐O*t*Bu x HCl)

A two‐necked, 50 mL round‐bottom flask was charged with **8 a** (1.63 g, 4.14 mmol), Pd/C (10 % w/w, 1.6 g) and were methanol (16 mL) were added and cyclohexene (8 mL) was added by syringe. The flask was placed in an oil bath preheated to 80 °C and the suspension was refluxed for exactly 7 minutes. The flask was immersed into a dry ice bath to terminate the reaction. The mixture was quickly filtered through celite and the solids were washed with methanol (35 mL). The solvent was sporadically evaporated *in vacuo* and the residue was taken up in diethyl ether (80 mL). The resulting solution was cooled to −78 °C and 4 M HCl in dioxane (1.2 mL) was added dropwise while stirring, leading to the formation of a white precipitate. After stirring for an additional 15 minutes, the solids were filtered off, washed with diethyl ether (30 mL) and dried *in vacuo* to yield Dil‐O*t*BuxHCl (1.10 g, 3.72 mmol, 90 % yield) as a white solid. The compound was spectroscopically identical to a commercially obtained sample (Aurum Pharmatech, US) and in good agreement with literature reports.[^33^, ^34^] **MS** (ESI) m/z (%): 260 (100) [*M*+H]^+^.

### Cbz*N*‐Val‐Dil‐O*t*Bu 14

A 250 mL round‐bottom flask was charged with Dil‐O*t*BuxHCl (2.77 g, 10.7 mmol, 1.0 eq.) and Cbz‐Val (4.56 g, 18.1 mmol, 1.7 eq.), the solids were dissolved in DCM (50 mL) and the flask was sealed with a septum. The mixture was cooled to −20 °C (acetone/dry ice) and 2‐bromo‐1‐ethylpyridinium tetrafluoroborate (5.86 g, 21.4 mmol, 2.0 eq.) was added, followed by *N,N*‐di*iso*propylethylamine (7.44 mL, 42.7 mmol, 4.0 eq.). The mixture was kept at −20 °C for one hour and was subsequently stirred at room temperature for 20 hours. The reaction mixture was then diluted with DCM (50 mL), washed with brine (2×50 mL), dried over MgSO_4_ and filtered. The yellow oil obtained after evaporation of the solvent was taken up in DCM (100 mL), silica (50 g) was added and the solvent was removed *in vacuo* to yield a dry, faintly yellow powder. The latter was washed with EtOAc/heptane (2 : 3, 300 mL) and the solvent was removed *in vacuo* to yield a yellow oil, which was further purified by flash chromatography on silica (petroleum ether/EtOAc 7 : 3) to yield **14** (4.48 g, 9.08 mmol, 85 % yield) as a transparent oil, which crystallized upon prolonged standing. The procedure is based upon a previously published method.^[36]^ **MS** (ESI) m/z (%): 493 (100) [*M*+H]^+^, 437 (34) [*M*‐*t*Bu+H]^+^. ^**1**^ **H NMR** (400 MHz, CDCl_3_), mixture of two rotamers: δ=7.33–7.27 (m, 5H), 5.63 and 5.50 (d, *J*=8.9 Hz and *J*=9.2 Hz, 1H), 5.07 (s and s, 2H), 4.78–4.56 (m, 1H), 4.49 (dd, *J*=9.2, 5.7 Hz, 1H), 3.98–3.92 and 3.91–3.81 (m and m, 1H), 3.32 3.31 (s and s, 3H), 2.93 and 2.74 (s and s, 3H), 2.42 (dd, *J*=15.6, 2.8 Hz, 1H), 2.28 (dd, *J*=15.6, 9.2 Hz, 1H), 2.03–1.92 (m, 1H), 1.43 and 1.42 (br s and br s, 9H), 0.98 (d, *J*=6.8 Hz, 3H), 0.94 (d, *J*=6.7 Hz, 3H), 0.89 (d, *J*=6.8 Hz, 3H), 0.81 (t, *J*=7.4 Hz, 3H). ^**13**^ **C NMR** (101 MHz, CDCl_3_), mixture of rotamers: δ=173.7 and 173.2, 171.2 and 171.1, 156.5 and 156.2, 136.5, 128.5, 128.4, 128.0, 127.9, 81.1 and 80.9, 78.2, and 77.2, 66.8 and 66.7, 64.0 and 60.4, 58.2 and 57.9, 56.0 and 55.6, 38.5 and 37.8, 35.1 and 33.3, 31.5 and 31.1, 30.5, 28.1 and 28.0, 26.7 and 25.9, 21.1 and 19.7, 17.0, 16.2 and 15.8, 14.2, 12.2 and 11.0.

### *N*,*N*‐Dimethylvaline 17

A 500 mL round‐bottom flask was charged with *L*‐valine (501 mg, 4.28 mmol, 1.0 eq.), Pd/C (10 % w/w, 0.10 g) was added, the flask was sealed with a septum, and the atmosphere was evacuated and backfilled with nitrogen three times. MeOH (100 mL) was injected by syringe, followed by aqueous formaldehyde (37 % w/w, 0.71 mL, 9.39 mmol, 2.2 eq.) and a stream of hydrogen gas was bubbled through the suspension for four hours. The mixture was then stirred in a hydrogen atmosphere for 18 hours. The suspension was filtered through celite, the plug was washed with MeOH (30 mL) and the solvent was removed *in vacuo* to yield a colourless oil which crystallized readily upon standing. **17** was obtained as a crystalline, off‐white solid (614 mg, 4.26 mmol, 99 % yield). The procedure is an exact reproduction of a published method and the products’ spectra were in good agreement with literature.^[37]^ TLC (*n*BuOH/AcOH/H_2_O 4 : 1 : 1, KMnO_4_) R_f_ = 0.31.

### Dov‐Val‐Dil‐O*t*Bu 15

A 25 mL round‐bottom flask was charged with **14** (177 mg, 359 μmol, 1.0 eq.) and a stir bar was added. MeOH (4 mL) and Pd/C (10 % w/w, 76 mg) were added. The flask was sealed with a septum, the atmosphere was evacuated and backfilled with nitrogen three times. A stream of hydrogen gas was bubbled through the suspension for two hours. The solids were filtered off over celite and the filter plug was washed with water (5 mL) and MeOH (50 mL). The solvents were removed *in vacuo* to yield crude Val‐Dil‐OtBu (128 mg), which was used directly in the next step without further purification.

A 10 mL vial was charged with *N*,*N*‐dimethylvaline (104 mg, 716 μmol, 2.0 eq.), DCM (2.4 mL) and triethylamine (98 μL, 717 μmol) were added, and the resulting suspension was stirred on ice. Diethyl cyanophosphonate (108 μL, 712 μmol, 2.0 eq.) was added and the reaction mixture was stirred on ice for five minutes before being added to another 10 mL vial containing Val‐Dil‐O*t*Bu (128 mg, 357 μmol, 1.0 eq.). The reaction mixture was stirred on ice for 20 minutes and then at room temperature for 40 minutes. The solvent was removed *in vacuo*, and the residue was purified by flash chromatography on silica (petroleum ether/ acetone 9 : 1) to yield **17** (95 mg, 196 μmol, 55 % yield over two steps) as a crystalline, off‐white solid. The products’ spectra were in good agreement with literature.^[52]^ **HRMS** (ESI) m/z [*M*+H]^+^ calcd for C_26_H_52_N_3_O_5_: 486.3901, found: 486.3891. ^**1**^ **H NMR** (400 MHz, CDCl_3_) δ 6.87 (d, *J*=9.0 Hz, 1H), 4.86–4.62 (m, 2H), 3.92–3.83 (m, 1H), 3.34 (s, 3H), 2.99 (s, 3H), 2.49–2.41 (m, 2H), 2.33–2.29 (m, 1H), 2.24 (s, 6H), 2.10–1.99 (m, 2H), 1.71–1.61 (m, 1H), 1.45 (s, 9H), 1.36–1.25 (m, 2H), 1.02 (d, *J*=6.8 Hz, 3H), 0.99 (d, *J*=6.8 Hz, 3H), 0.96 (d, *J*=5.5 Hz, 3H), 0.94 (d, *J*=5.4 Hz, 3H), 0.91 (d, *J*=6.7 Hz, 3H), 0.80 (t, *J*=7.4 Hz, 3H). ^**13**^ **C NMR** (101 MHz, CDCl_3_) δ 173.5, 171.9, 171.3, 81.0, 78.3, 76.7, 63.9, 58.1, 53.9, 43.1, 38.7, 33.2, 31.1, 28.3, 27.9, 25.9, 20.4, 19.9, 18.3, 18.0, 15.9, 10.9.

### *N*Boc‐*O*Ac‐L‐hydroxyproline pentafluorophenyl ester 20

A 500 mL round‐bottom flask was charged with trans‐4‐hydroxy‐L‐proline (**19**, 15.0 g, 114 mmol, 1.0 eq.) and a solution of sodium hydroxide (5.5 g, 138 mmol, 1.2 eq.) in water (75 mL) was added. The solids dissolved upon stirring. Over the course of 30 minutes, a solution of di‐(*tert*‐butyl)‐dicarbonate (29 mL, 126 mmol, 1.1 eq.) in THF (150 mL) was added. The resulting two‐phasic mixture was stirred vigorously for 48 hours. Water was added (75 mL) to dissolve the solids, the phases were separated, and the aqueous phase was washed with diethyl ether (3×50 mL). It was then acidified to pH 2 with aqueous 1 M NaHSO_4_ and extracted with EtOAc (4×150 mL). The combined organic layers were dried over MgSO_4_, the drying agent was filtered off and the solvent was removed *in vacuo* to yield crude (2*S*,4*R*)‐1‐(*tert*‐butoxycarbonyl)‐4‐hydroxypyrrolidine‐2‐carboxylic acid (16.7 g) as a thick syrup, which was used in the next step without further purification. TLC (*n*BuOH/AcOH/H_2_O 4 : 1 : 1, ninhydrin+Δ) R_f_ =0.6.

To a 250 mL round‐bottom flask containing crude (2*S*,4*R*)‐1‐(*tert*‐butoxycarbonyl)‐4‐hydroxypyrrolidine‐2‐carboxylic acid (16.7 g, 72.2 mmol, 1.0 eq.) were added DCM (35.2 mL) and pyridine (35.2 mL, 435 mmol, 6.0 eq.) were added and the resulting two‐phasic mixture was ultrasonicated for ten minutes. Then, while stirring on ice, acetic anhydride (34.3 mL, 363 mmol, 5.0 eq.) was added via syringe, and the resulting mixture was stirred on ice for one hour and then at room temperature for five hours. The solvent was removed *in vacuo* and the residue was co‐evaporated with pyridine several times. Water (50 mL) was added, the mixture was ultrasonicated until nearly homogenous, and was then freeze‐dried to yield crude (2*S*,4*R*)‐4‐acetoxy‐1‐(*tert*‐butoxycarbonyl)pyrrolidine‐2‐carboxylic acid (20.9 g) as a thick, yellow syrup, which was used in the next step without further purification despite containing solvent residues.

To a 500 mL round‐bottom flask containing crude (2*S*,4*R*)‐4‐acetoxy‐1‐(*tert*‐butoxycarbonyl)pyrrolidine‐2‐carboxylic acid (20.9 g, 76.5 mmol, 1.0 eq.) was added pentafluorophenol (18.7 g, 102 mmol, 1.3 eq.), followed by EtOAc (150 mL). The mixture was stirred until a solution had been obtained. Dicyclohexylcarbodiimide (21.1 g, 102.3 mmol, 1.3 eq.) was added and the mixture was stirred at room temperature for 16 hours. The resulting suspension was filtered, and the solids were washed with EtOAc (2×100 mL). The filtrate was concentrated in vacuo and purified by flash chromatography on silica (petroleum ether/EtOAc 1 : 1) to give **20** (30.1 g, 68.6 mmol, 60 % yield over three steps) as a colourless oil. **HRMS** (ESI) m/z [M+Na]^+^ calcd for C_18_H_18_F_5_NNaO_6_: 462.0946, found: 462.0910. ^**1**^ **H NMR** (400 MHz, CDCl_3_), mixture of two rotamers: δ=5.38–5.31 (m, 1H), 4.75–4.66 and 3.82–3.70 (m and m, 2H), 3.64–3.56 (m, 1H), 2.67–2.52 (m, 1H), 2.46–2.33 (m, 1H), 2.09 and 2.08 (s and s, 3H), 1.48 and 1.46 (s and s, 9H). ^**13**^ **C NMR** (101 MHz, CDCl_3_), mixture of two rotamers: δ=170.5 and 170.4, 168.9 and 168.4, 154.2 and 153.5, 142.4 and 141.0, 140.0 and 139.3, 138.6 and 136.8, 124.9, 81.7 and 81.3, 72.7 and 71.7, 57.54 and 57.52, 52.4 and 52.2, 37.1 and 35.7, 28.4 and 28.2, 21.1. ^**19**^ **F NMR** (376 MHz, CDCl_3_) δ=‐152.2 and −152.9 (m and m, 2F), −157.1 and −157.7 (t, *J*=21.6 Hz and t, *J*=21.7 Hz, 1F), −161.7 and −162.2 (m and m, 2F).

### *N*Boc‐*O*Ac‐β‐keto‐γ‐amino acid ethyl ester 9 b

A 250 mL round‐bottom flask was charged with a stir bar and 3‐ethoxy‐2‐methyl‐3‐oxopropanoic acid (5.1 g, 34.9 mmol, 1.5 eq.). THF (15 mL) was added and the resulting mixture was cooled on ice. Dropwise, under a nitrogen atmosphere, a solution of *iso*‐propylmagnesium bromide in 2‐Me‐THF (24 mL, 2.9 M, 69.6 mmol, 3.0 eq.) was added via syringe. The mixture was allowed to attain room temperature, where it was stirred for one hour. It was then cooled back on ice and a solution of **20** (10.2 g, 23.2 mmol, 1.0 eq.) in THF (30 mL) was added over the period of five minutes. The reaction was stirred at room temperature for two days, whereupon it was quenched by pouring the reaction mixture into 25 mL of ice‐cold methanol while stirring. The solvent was evaporated *in vacuo*, the residues were suspended in ethyl acetate (200 mL) and the suspension was transferred to a separatory funnel. To the separatory funnel were added more ethyl acetate (150 mL), hexanes (50 mL), and half‐saturated brine (100 mL) and the phases were separated. The aqueous phase was diluted with more water (100 mL) and extracted with ethyl acetate (2×250 mL). The pooled organic layers were dried over MgSO_4_, the solids were removed by filtration and the solvent was removed *in vacuo*. The residue was purified by flash chromatography on silica (petroleum ether/EtOAc 17 : 3→4 : 1) to yield **9 b** (6.4 g, 16.7 mmol, 72 % yield) as a colourless oil. **HRMS** (ESI) m/z [*M*+H]^+^ calcd for C_17_H_28_NO_7_: 358.1866, found: 358.1851. ^**1**^ **H NMR** (400 MHz, CD_3_CN), mixture of two rotamers of each of two diastereoisomers: δ=5.23–5.12 (m, 1H), 4.73–4.45 (m, 1H), 4.13 and 4.12 (q, *J*=7.1 Hz, and q, *J*=7.1 Hz, 2H), 3.86 and 3.83–3.72 (q, *J*=7.1 Hz, and m, 1H), 3.67–3.42 (m, 1H), 2.46–2.25 (m, 1H), 2.22–2.06 (m, 1H), 2.00 and 2.00 and 1.99 (s, 3H), 1.43 and 1.40 and 1.39 and 1.37 (s, 9H), 1.31–1.18 (m, 6H). ^**13**^ **C NMR** (101 MHz, CD_3_CN), mixture of two rotamers of each of two diastereomers: δ 207.2 and 207.0 and 206.6 and 206.6, 171.6 and 171.6, 171.6 and 171.5 and 171.4 and 171.2, 156.1 and 155.5 and 155.2 and 154.8, 81.7 and 81.5 and 81.5 and 81.3, 74.4 and 74.1 and 73.4 and 73.2, 64.7 and 64.1 and 63.77, 62.59 and 62.53, 53.9 and 53.8 and 53.5 and 53.5, 52.36 and 51.60 and 50.62 and 50.39, 37.1 and 37.0 and 36.5 and 35.8, 28.9 and 28.8, 21.6, 14.8 and 14.8, 13.9 and 13.9 and 13.7 and 13.5.

### *N*Boc‐*O*Ac‐β‐hydroxy‐γ‐amino acid ethyl ester (*R*,*R*)‐22

A 50 mL round‐bottom flask was charged with **9 b** (845 mg, 2.36 mmol, 1.0 eq.), manganese(II)‐chloride (596 mg, 4.74 mmol, 2.0 eq.) and a stir bar. MeOH (11.8 mL) was added, and the mixture was cooled on ice. While stirring, sodium borohydride (180 mg, 4.76 mmol, 2.0 eq.) was added in increments over the period of ten minutes. The mixture was stirred on ice for one hour, after which the solvent was removed *in vacuo*. The residues were taken up in EtOAc/MeOH 9 : 1 (50 mL), the resulting suspension was filtered through a silica plug. Upon removal of the solvent, a mixture of four diastereoisomers of **22** was obtained (807 mg, 2.25 mmol, 95 % yield). The isomers were separated by flash chromatography on silica (petroleum ether/EtOAc 3 : 1→7 : 3). **(*R***,***R*** **)‐22** eluted *after* the (*S*,*S*)‐isomer and was obtained in 35 % yield (297 mg, 0.83 mmol) as a colourless oil. **(*R***,***R*** **)‐22. HRMS** (ESI) m/z [M+H]^+^ calcd for C_17_H_30_NO_7_: 360.2017, found: 360.2009. **[α]_D_** ^**20**^ −42.4° (c 0.03, MeOH). ^**1**^ **H NMR** (400 MHz, CD_3_CN), mixture of two rotamers: δ=5.18–5.08 (m, 1H), 4.19–4.09 (m, 1H), 4.10–3.99 (m, 2H), 3.94–3.79 (m, 1H), 3.65–3.52 (m, 1H), 3.46–3.22 (m, 2H), 2.40–2.29 (m, 1H), 2.29–2.16 (m, 1H), 2.00–1.95 (m, 4H), 1.44 (br s, 9H), 1.22 (t, *J*=7.1 Hz, 3H), 1.16 (d, *J*=6.8 Hz, 3H). ^**13**^ **C NMR**, mixture of two rotamers: (101 MHz, CD_3_CN) δ 175.5 and 175.2, 171.3, 155.6 and 155.2, 80.2, 74.2 and 73.9, 73.2 and 72.5, 61.1 and 61.0, 60.0 and 58.8, 53.7 and 53.0, 44.6 and 44.2, 31.9 and 31.7, 28.6, 21.2, 14.5 and 14.4, 14.47 and 14.45.

### Bicyclic lactam (*R*,*R*)‐23

A 4 mL vial was charged with **(*R***,***R*** **)‐22** (24 mg, 67 μmol 1.0 eq.), a stirring bar was added, followed by DCM (334 μL) and TFA (334 μL). The resulting solution was stirred for 90 minutes, whereupon the solvent was removed *in vacuo*. Cesium carbonate (49 mg, 150 μmol, 2.2 eq.) was added, followed by non‐dry MeOH (334 μL). The mixture was stirred for one hour, during which it became a homogenous solution. The solvent was removed *in vacuo* and the oily residue was dried under fine vacuum for one day before NMR‐spectroscopic analyses were performed. **HRMS** (ESI) m/z [*M*+H]^+^ calcd for C_8_H_14_NO_3_: 172.0968, found: 172.0966. ^**1**^ **H NMR** (400 MHz, DMSO‐*d*_6_) δ=5.50 (dt, *J*=5.5, 1.3 Hz, 1H), 5.06–5.03 (m, 1H), 4.40–4.34 (m, 1H), 3.71 (ddd, *J*=9.3, 6.4, 6.0 Hz, 1H), 3.53–3.46 (m, 2H), 2.74 (d, *J*=11.9 Hz, 1H), 2.56–2.46 (m obscured by solvent peak, 1H), 1.96 (ddd, *J*=12.6, 6.0, 1.6 Hz, 1H), 1.51 (ddd, *J*=12.8, 9.4, 5.1 Hz, 1H), 1.00 (d, *J*=7.0 Hz, 2H). ^**13**^ **C NMR** (101 MHz, DMSO‐*d*_6_) δ=173.6, 81.0, 71.9, 64.6, 50.7, 47.8, 39.3, 12.7.

### Bicyclic lactam (*S*,*S*)‐23

A 4 mL vial was charged with **(*S***,***S*** **)‐22** (24 mg, 67 μmol 1.0 eq.), a stirring bar was added, followed by DCM (334 μL) and TFA (334 μL). The resulting solution was stirred for 90 minutes, whereupon the solvent was removed *in vacuo*. Cesium carbonate (49 mg, 150 μmol, 2.2 eq.) was added, followed by non‐dry MeOH (334 μL). The mixture was stirred for one hour, during which it became a homogenous solution. The solvent was removed *in vacuo* and the oily residue was dried under fine vacuum for one day before NMR‐spectroscopic analyses were performed. **HRMS** (ESI) m/z [*M*+H]^+^ calcd for C_8_H_14_NO_3_: 172.0968, found: 172.0960. ^**1**^ **H NMR** (400 MHz, DMSO‐*d*_6_) δ=5.11 (d, *J*=4.3 Hz, 1H), 5.05 (d, *J*=3.8 Hz, 1H), 4.42–4.34 (m, 1H), 4.18 (ddd, *J*=9.5, 6.5, 4.4 Hz, 2H), 3.87 (dd, *J*=4.4, 4.3 Hz, 1H), 3.47 (dd, *J*=11.7, 5.2 Hz, 1H), 2.70 (dd, *J*=11.8, 2.4 Hz, 1H), 2.19 (q, *J*=7.7 Hz, 1H), 1.94 (ddd, *J*=12.8, 9.4, 5.4 Hz, 1H), 1.53 (ddd, *J*=12.8, 6.7, 2.4 Hz, 1H), 1.12 (d, *J*=7.7 Hz, 3H). ^**13**^ **C NMR** (101 MHz, DMSO‐d_6_) δ=176.5, 72.4, 72.1, 62.3, 51.0, 50.4, 32.6, 13.7.

### *N*Boc‐*O*Ac‐β‐methoxy‐γ‐amino acid ethyl ester 8b

A 10 mL round‐bottom flask wash charged with **(*R***,***R*** **)‐22** (123 mg, 342 μmol, 1.0 eq.), THF (2.5 mL) and DMF (855 μL). Dimethylsulfate (81 μL, 856 μmol, 2.5 eq.) was added, the flask was sealed with a septum, a nitrogen atmosphere was applied, and the reaction mixture was cooled to −15 °C (NaCl/ice). Sodium hydride (16 mg, 60 % dispersion over mineral oil, 400 μmol, 1.2 eq.) was added and the mixture was stirred at −15 °C for 30 minutes and at 0 °C for five hours. EtOAc (5 mL) was added, followed by half‐saturated aqueous NH_4_Cl (2 mL). The resulting two‐phasic mixture was stirred at room temperature for 30 minutes, was the transferred to a separatory funnel, diluted with water (10 mL), and the phases were separated. The aqueous layer was extracted with DCM (4×10 mL), the organic layers were pooled and dried over MgSO_4_. Upon filtration and evaporation of the solvent *in vacuo*, a crude oil was obtained, which was purified by flash chromatography on silica (petroleum ether/EtOAc 9 : 1→3 : 1) to give **8 b** (102 mg, 273 μmol, 80 % yield) as a colourless oil. **HRMS** (ESI) m/z [*M*+Na]^+^ calcd for C_18_H_31_NO_7_Na: 396.1993, found: 396.1964. ^**1**^ **H NMR** (400 MHz, CDCl_3_), mixture of two rotamers: δ 5.28–5.16 (m, 1H), 4.24–4.06 (m, 2H), 4.06–3.93 (m, 1H), 3.93–3.85 and 3.62–3.52 (m and m, 1H), 3.84–3.70 (m, 1H), 3.48–3.36 (m, 1H), 3.42 (s, 3H), 2.46–2.33 (m, 1H), 2.33–2.18 (m, 1H), 2.05–1.95 (m, 1H), 2.01 (s, 3H), 1.51 and 1.45 (br s and br s, 9H), 1.25 (t, *J*=7.1 Hz, 3H), 1.28–1.20 (m, 6H). ^**13**^ **C NMR (**101 MHz, CDCl_3_), mixture of two rotamers: δ 174.3, 170.8 and 170.7, 154.5 and 154.4, 82.9 and 81.6, 80.3 and 79.8, 73.5 and 73.0, 61.5 and 61.1, 60.6, 58.8 and 58.5, 52.9 and 52.4, 43.3, 32.0 and 31.3, 29.8 and 28.6, 21.3, 14.3, 14.1.

### *N*Boc‐hydroxy‐β‐methoxy‐γ‐amino acid ethyl ester

A 100 mL round‐bottom flask was charged with **8 b** (500 mg, 1.34 mmol, 1.0 eq.), MeOH (48 mL) was added, followed by a solution of potassium carbonate (185 mg, 1.34 mmol, 1.0 eq.) in water (1.2 mL). The resulting solution was stirred on ice for four hours. While continuing to stir on ice, HCl in EtOH (2.14 mL, 1.25 M, 2.68 mmol, 2.0 eq.) was added and the solvent was removed *in vacuo*. The resulting semi‐solid was co‐evaporated with EtOAc twice, taken up in EtOAc/MeOH 10 : 1 (27.5 mL), and filtered through a silica plug, which was subsequently washed with hexanes/EtOAc 1 : 1 (50 mL), and the solvent was evaporated *in vacuo. tert*‐butyl (2*S*,4*R*)‐2‐((1*R*,2*R*)‐3‐ethoxy‐1‐methoxy‐2‐methyl‐3‐oxopropyl)‐4‐hydroxypyrrolidine‐1‐carboxylate (*N*Boc‐hydroxy‐β‐methoxy‐γ‐amino acid ethyl ester, 430 mg, 1.30 mmol, 97 % yield) was obtained as a colourless oil. **HRMS** (ESI) m/z [*M*+Na]^+^ calcd for C_16_H_29_NO_6_: 354.1918, found: 354.1893. ^**1**^ **H NMR** (400 MHz, CDCl_3_), mixture of two rotamers: δ 4.47–4.36 (m, 1H), 4.21–4.06 (m, 2H), 4.06–3.79 (m, 2H), 3.70–3.47 (m, 1H), 3.44 and 3.42 (s and s, 3H), 3.37–3.27 (m, 1H), 2.48–2.31 (m, 1H), 2.21–2.11 (m, 1H), 2.08–1.96 (m, 1H), 1.96–1.86 (m, 1H), 1.49 and 1.46 (br s and br s, 9H), 1.25 (t, *J*=7.1 Hz, 3H), 1.23–1.18 (m, 3H). ^**13**^ **C NMR** (101 MHz, CDCl_3_), mixture of two rotamers: δ=174.4, 154.8, 82.9 and 81.6, 80.2 and 77.4, 70.6 and 70.1, 61.4, 60.7, 58.4 and 58.3, 55.7 and 55.2, 43.4, 34.5, 28.7 and 28.5, 14.3, 14.1.

### *N*Boc‐azido‐β‐methoxy‐γ‐amino acid ethyl ester 24

A 10 mL vial, equipped with a stir bar, was charged with triphenylphosphine (79 mg, 300 μmol, 2.0 eq.), the vial was capped, and the atmosphere was evacuated and refilled with nitrogen. THF (0.75 mL) was added by syringe, the vial was cooled on ice and diisopropyl azodicarboxylate (59 μL, 300 μmol, 2.0 eq.) was added. After one hour on ice, a suspension had formed. A solution of tert‐butyl (2S,4R)‐2‐((1R,2R)‐3‐ethoxy‐1‐methoxy‐2‐methyl‐3‐oxopropyl)‐4‐hydroxypyrrolidine‐1‐carboxylate (49 mg, 148 μmol, 1.0 eq.) in THF (0.75 mL) was added, followed 15 minutes later by diphenyl phosphoryl azide (65 μL, 302 μmol, 2.0 eq.). The mixture was stirred on ice for one hour and then at room temperature for 24 hours. The reaction mixture was transferred to a round‐bottom flask, the solvent was evaporated and the residue was purified by flash chromatography on silica (petroleum ether/EtOAc, 9 : 1→17 : 3) to yield **24** (45 mg, 126 μmol, 85 % yield) as a colourless oil contaminated with traces of triphenylphosphine oxide (according to ^13^C‐NMR). **HRMS** (ESI) m/z [*M*+Na]^+^ calcd for C_16_H_28_N_4_NaO_5_: 379.1952, found: 379.1830. ^**1**^ **H NMR** (400 MHz, CD_3_CN) δ=4.19–3.98 (m, 1H), 3.93–3.83 (m, 1H), 3.85–3.76 (m, 1H), 3.40 (s, 3H), 3.00–2.85 (m, 1H), 2.42 (p, *J*=7.0 Hz, 1H), 2.30–2.20 (m, 1H), 2.00–1.95 (m, 1H), 1.44 (s, 9H), 1.23 (t, *J*=7.1 Hz, 3H), 1.17 (d, *J*=6.9 Hz, 3H). ^**13**^ **C NMR** (101 MHz, CD_3_CN) δ=175.2, 154.2, 83.4, 80.5, 61.3, 59.7, 59.0, 52.2, 51.2 43.6, 31.6, 28.6, 14.5, 13.5.

### *N*Boc‐4‐(Cbz‐amino)Dap‐Phe‐OMe 26

A 10 mL vial, equipped with a stir bar, was charged with **24** (45 mg, 126 μmol, 1.0 eq.), followed by Pd/C (10 % w/w, 5 mg) and the vial was capped. The atmosphere was evaporated and backfilled with nitrogen three times. EtOAc (2 mL) was added by syringe, a hydrogen balloon was installed, and the reaction mixture was stirred at room temperature for 18 hours. It was then filtered through a 450 nm syringe filter, which was subsequently washed with more EtOAc (2 mL), and the resulting solution was evaporated to dryness to yield crude *tert‐*butyl (*2S,4S*)‐4‐amino‐2‐((*1R,2R*)‐3‐ethoxy‐1‐methoxy‐2‐methyl‐3‐oxopropyl)pyrrolidine‐1‐carboxylate (46 mg) as a colourless oil, which was used directly in the next step without purification.

A 10 mL vial was charged with crude *tert‐*butyl (*2S,4S*)‐4‐amino‐2‐((*1R,2R*)‐3‐ethoxy‐1‐methoxy‐2‐methyl‐3‐oxopropyl)pyrrolidine‐1‐carboxylate (46 mg, 139μmol, 1.0 eq.), capped, cooled on ice, and by syringe, a solution of benzyl chloroformate (26 μL, 182 μmol, 1.3 eq.) in DCM (0.5 mL) was added, followed by a solution of triethylamine (38 μL, 278 μmol, 2.0 eq.) in DCM (0.5 mL). The resulting solution was stirred on ice for 30 minutes and at room temperature for two hours. The reaction mixture was subsequently filtered through a silica plug, which was washed with more DCM (5 mL) and the volatiles were removed *in vacuo* to yield crude *tert*‐butyl (2*S*,4*S*)‐4‐(((benzyloxy)carbonyl)amino)‐2‐((1*R*,2*R*)‐3‐ethoxy‐1‐methoxy‐2‐methyl‐3‐oxopropyl)pyrrolidine‐1‐carboxylate (42 mg) as a colourless oil, which was used directly in the next step without purification.

A 10 mL round‐bottom flask was charged with crude *tert*‐butyl (2*S*,4*S*)‐4‐(((benzyloxy)carbonyl)amino)‐2‐((1*R*,2*R*)‐3‐ethoxy‐1‐methoxy‐2‐methyl‐3‐oxopropyl)pyrrolidine‐1‐carboxylate (42 mg, 90 μmol, 1.0 eq.) and a stir bar. MeOH (2 mL) was added, the resulting solution was cooled to 0 °C (refrigerator) and a solution of lithium hydroxide (65 mg, 2.71 mmol, 30 eq.) in water (2 mL) was added dropwise. The reaction mixture was stirred at 0 °C for 62 hours, whereupon it was diluted with water (20 mL) and acidified to pH 2 using aqueous 1 M NaHSO_4_. The mixture was subsequently extracted with EtOAc (3×15 mL), the organic layers were dried over Na_2_SO_4_, the drying agent was removed by filtration and the solvent was removed *in vacuo* to yield crude (2*R*,3*R*)‐3‐((2*S*,4*S*)‐4‐(((benzyloxy)carbonyl)amino)‐1‐(tert‐butoxycarbonyl)pyrrolidin‐2‐yl)‐3‐methoxy‐2‐methylpropanoic acid (33 mg), which was used directly in the next step without purification. **HRMS** (ESI) m/z [M‐*t*Bu+H]^+^ calcd for C_17_H_25_N_2_O_5_: 337.1758, found: 337.1718.

A 5 mL round‐bottom flask was charged with crude (2*R*,3*R*)‐3‐((2*S*,4*S*)‐4‐(((benzyloxy)carbonyl)amino)‐1‐(tert‐butoxycarbonyl)pyrrolidin‐2‐yl)‐3‐methoxy‐2‐methylpropanoic acid (33 mg, 76 μmol, 1.0 eq.) and a stir bar was added, followed by L‐phenylalanine methyl ester hydrochloride (24 mg, 113 μmol, 1.5 eq.). The flask was sealed with a septum and a solution of diisopropylethylamine (40 μL, 227 μmol, 3.0 eq) in DCM (2.5 mL) was added, the solution was cooled on ice, and HATU (43 mg, 113 μmol, 1.5 eq.) was added in once increment. The resulting suspension was stirred vigorously on ice for 30 minutes and then at room temperature for two hours. The solvent was removed *in vacuo*, the residue was taken up in MeOH (5 mL) and was dispersed over silica (1 g). The dispersion was purified by flash chromatography on silica (petroleum ether/EtOAc 1 : 1→0 : 1) to afford **26** as a colourless oil (44 mg, 74 μmol, 58 % yield over four steps). **HRMS** (ESI) m/z [*M*+H]^+^ calcd for C_32_H_44_N_3_O_8_: 598.3123, found: 598.3113. **[α]_D_** ^**20**^ −29.9° (c 0.40, MeOH). ^**1**^ **H NMR** (400 MHz, CDCl_3_), mixture of two rotamers: δ=7.39–7.04 (m, 10H, obscured by CHCl3), 6.39 and 6.09 (br s and br s, 2H), 5.97 (s, 1H), 5.18–5.04 (m, 2H), 4.90–4.77 (m, 1H), 4.19–4.06 (m, 1H), 3.98–3.91 and 3.91–3.83 (m and m, 1H), 3.79–3.66 (m, 4H), 3.61–3.48 (m, 1H), 3.41 (s, 3H), 3.23–3.17 (m, 1H), 3.17–3.11 (m, 1H), 3.05–2.96 (m, 1H), 2.36–2.24 and 2.24–2.11 (m and m, 1H), 2.02–1.92 and 1.92–1.79 (m and m, 1H), 1.64–1.54 (m, 1H), 1.46 (s, 9H), 1.18 (d, *J*=6.9 Hz, 3H). ^**13**^ **C NMR** (101 MHz, CDCl_3_), mixture of two rotamers: δ 173.5 and 173.1, 172.4, 155.9, 155.0 and 154.4, 136.8, 136.3, 129.3, 128.8, 128.6, 128.2, 128.1, 127.3, 83.6 and 82.4, 80.4 and 80.0, 66.6, 60.8 and 58.6, 54.5 and 53.8, 53.3 and 52.8, 52.5, 49.5 and 48.6, 44.7 and 44.3, 37.8, 32.1 and 30.9, 28.6, 24.0, 14.8.

### Cbz‐azastatin methyl ester 27

A 10 mL round‐bottom flask was charged with Dov‐Val‐Dil‐O*t*Bu (**15**, 26.8 mg, 55.2 μmol), a stir bar and DCM (3 mL) were added and the resulting solution was cooled on ice. While stirring, trifluoroacetic acid (1.5 mL) was added, followed after one hour by more trifluoroacetic acid (1.5 mL). The solution was stirred at 0 °C (refrigerator) for 16 hours. The volatiles were removed *in vacuo* and the oily residue was co‐evaporated with DCM three times to yield crude Dov‐Val‐Dil‐OH x TFA (31.0 mg), which was used directly in the next step without purification.

A 10 mL round‐bottom flask was charged with *N*Boc‐4‐(Cbz‐amino)Dap‐Phe‐OMe (**26**, 32.0 mg, 53.5 μmol), a stir bar and DCM (4 mL) were added and the resulting solution was cooled on ice. While stirring, trifluoroacetic acid (1 mL) was added, followed after two hours by more trifluoroacetic acid (0.5 mL). After one more hour of stirring, the solvent was removed *in vacuo* and the oily residue was co‐evaporated with DCM three times to yield crude 4‐(Cbz‐amino)Dap‐Phe‐OMe x TFA (32.7 mg), which was used directly in the next step without purification.

A 10 mL round‐bottom flask was charged with solutions of crude 4‐(Cbz‐amino)Dap‐Phe‐OMe x TFA (32.7 mg, 53.5 μmol, 1.0 eq.) and crude Dov‐Val‐Dil‐OH x TFA (31.0 mg, 57.0 μmol, 1.1 eq.) in DCM (2 mL each) and the solvent was removed *in vacuo*. A stir bar was added, the flask was sealed with a septum, and 1,2‐dimethoxyethane (2 mL) was added by syringe. The resulting solution was stirred on ice, triethylamine (36.6 μL, 267 μmol, 5.0 eq.) and diethyl cyanophophonate (12.1 μL, 80.2 μmol, 1.5 eq.) were added, and the reaction mixture was stirred on ice for two hours. The solvent was then removed *in vacuo*, and the oily residue was purified by flash chromatography on silica (DCM/MeOH 99 : 1→97 : 3+NH_3_. *Note*: the eluent was prepared by extracting 1 mL of 28 % aqueous NH_4_OH with DCM and diluting the organic layer with the appropriate volume of MeOH) to yield **27** (36.5 mg, 40.2 μmol, 75 % yield over three steps) as a white solid. **HRMS** (ESI) m/z [M+H]^+^ calcd for C_49_H_77_N_6_O_10_: 909.5696, found: 909.5624. **[α]_D_** ^**20**^ −36.2° (c 0.19, MeOH). ^**1**^ **H NMR** (400 MHz, CD_2_Cl_2_), mixture of two rotamers: δ=7.39–7.12 (m, 1H), 6.87 and 6.81 (d and d, *J*=8.9 Hz and *J*=9.0 Hz, 1H), 6.48 and 6.44 (d and d, *J*=7.6 Hz and *J*=7.6 Hz, 1H), 6.12 and 5.88 (d and d, *J*=8.4 Hz and *J*=9.3 Hz, 1H), 5.13–5.02 (m, 2H), 4.96–4.78 (m, 1H), 4.78–4.69 (m, 2H), 4.23–4.14 (m, 1H), 4.14–4.04 (m, 2H), 4.03–3.88 (m, 1H), 3.76 and 3.70 (s and s, 3H), 3.69–3.64 (m, 1H), 3.40 and 3.38 (s and s, 3H), 3.36–3.32 (m, 1H), 3.31 and 3.29 (s, 3H), 3.27–3.10 (m, 2H), 3.03–2.95 (m, 3H), 2.42–2.25 (m, 4H), 2.25 and 2.23 (s and s, 6H), 2.10–1.95 (m, 3H), 1.76–1.59 (m, 2H), 1.43–1.26 (m, 2H), 1.22 and 1.16 (d and d, *J*=6.8 Hz and *J*=6.9 Hz, 3H), 1.00–0.89 (m, 15H), 0.82 (t, *J*=7.3 Hz, 3H). ^**13**^ **C NMR** (101 MHz, CD_2_Cl_2_) δ=173.7, 172.6, 172.4, 171.5, 171.1, 156.0, 137.4, 137.0, 129.6, 129.3, 129.1, 128.9, 128.8, 128.4, 128.3, 128.2, 127.3, 82.1, 78.6, 76.9, 66.7, 60.9, 59.4, 58.1, 55.1, 53.7, 52.6, 50.0, 44.9, 43.0, 37.93, 37.87, 33.3, 31.4, 30.7, 30.1, 28.0, 26.2, 20.3, 18.0, 14.9, 10.8.

### Azastatin methyl ester 7 and *N*‐ethyl azastatin methyl ester 28

A 10 mL round‐bottom flask was charged with a solution of **27** (28.0 mg, 30.8 μmol) in EtOH (1 mL) and the solvent was removed *in vacuo* to yield a glassy solid. A stir bar and Pd/C (10 % w/w, 5.6 mg) were added, the flask was sealed, and the atmosphere was evacuated and backfilled with nitrogen three times. EtOH (2 mL) was added by syringe and hydrogen was bubbled through the resulting solution for two hours, whereupon the reaction mixture was stirred under hydrogen for an additional 16 hours. It was then filtered through a 450 nm syringe filter, which was subsequently washed with more EtOH (2 mL), and the resulting solution was evaporated to dryness to yield 23.7 mg of an oily residue, which consisted of **7** and **28** (approximately 3 : 2 ratio, 18.1 μmol and 12.1 μmol, respectively, 30.2 μmol combined yield, 98 %) according to HPLC. A portion of each pure analyte was obtained by a series of purifications by flash chromatography on silica (first and second column: DCM/MeOH 97 : 3→93 : 7+NH_3_, third and fourth column: DCM/MeOH 9 : 1→4 : 1+NH_3_, *Note*: the eluent for all columns was prepared by extracting 1 mL of 28 % aqueous NH_4_OH with DCM and diluting the organic layer with the appropriate volume of MeOH) to yield 8.5 mg of **7**, 1.5 mg of **28** and 13.7 mg of a mixture of the two.

Azastatin‐OMe (**7**). **HRMS** (ESI) m/z [*M*+Na]^+^ calcd for C_41_H_70_N_6_NaO_8_: 797.5147, found: 797.5112. ^**1**^ **H NMR** (400 MHz, CD_3_OD), mixture of two rotamers: δ=7.38–7.14 (m, 5H), 4.82–4.72 (m obscured by solvent peak, 2H), 4.65 (d, *J*=8.7 Hz, 1H), 4.28–4.00 (m, 2H), 3.96–3.85 (m, 1H), 3.75 and 3.72 (s and s, 3H), 3.69–3.47 (m, 2H), 3.45 and 3.39 (s and s, 3H), 3.33 and 3.29 (s and s obscured by solvent peak, 3H), 3.29–3.25 (m, 1H), 3.24 and 3.15 (s and s, 3H), 3.09–2.87 (m, 2H), 2.71 and 2.69 (d and d, *J*=8.4 Hz and *J*=8.5 Hz, 1H), 2.56–2.40 (m, 2H), 2.34 and 2.32 (s and s, 6H), 2.30–2.20 (m, 1H), 2.14–2.00 (m, 2H), 1.96–1.87 (m, 1H), 1.84–1.71 (m, 2H), 1.39–1.27 (m, 4H), 1.24 and 1.18 (d, *J*=6.7 Hz and *J*=6.8 Hz, 3H), 1.11–0.94 (m, 12H), 0.90–0.83 (m, 6H). ^**13**^ **C NMR** (101 MHz, CD_3_OD), mixture of two rotamers: δ=176.3 and 176.0, 175.5 and 175.3, 173.6 and 173.5, 173.1 and 173.0, 172.2 and 171.8, 138.6, 138.5, 130.2, 130.0, 129.7, 129.6, 127.9, 127.8, 86.2, 82.7, 79.5 and 79.2, 75.8 and 75.7, 62.3 and 61.7, 60.3, 58.5 and 58.3, 57.7 and 57.6, 56.2 and 56.0, 54.5 and 54.0, 52.9 and 52.8, 45.4 and 45.1, 42.51 and 42.45, 38.1 and 37.9, 36.4, 33.5 and 33.0, 31.7, 30.7, 28.8, 27.0, 20.22 and 20.19, 20.0 and 19.5, 19.35 and 19.32, 16.2 and 15.9, 15.6, 15.2, 10.9 and 10.8. For **MS**‐fragmentation pattern please refer to SI.

*N*‐Ethyl azastatin‐OMe (**28**). **HRMS** (ESI) m/z [*M*+Na]^+^ calcd for C_43_H_74_N_6_NaO_8_: 825.5460, found: 825.5413. ^**1**^ **H NMR** (400 MHz, CD_3_OD), mixture of rotamers: δ=^**1**^ **H NMR** (400 MHz, CD_3_OD), mixture of rotamers; δ=7.36–7.16 (m, 5H), 4.80–4.75 (m obscured by solvent peak, 1H), 4.62–4.55 (m, 2H), 4.29–4.21 (m, 1H), 4.18–4.10 (m, 1H), 4.10–4.03 (m, 1H), 3.99–3.92 (m, 1H), 3.76 and 3.73 (s and s, 3H), 3.69–3.64 (m, 1H), 3.62–3.54 (m, 1H), 3.54–3.49 (m, 1H), 3.43 and 3.38 (s and s, 3H), 3.36–3.33 and 3.29–3.26 (m obscured by solvent peak, 3H), 3.25 and 3.15 (s and s, 3H), 3.07–2.99 (m, 1H), 2.99–2.86 (m, 3H), 2.86–2.72 (m, 2H), 2.58–2.47 (m, 2H), 2.40 and 2.37 (s and s, 6H), 2.23–2.11 (m, 2H), 2.11–2.00 (m, 2H), 1.92–1.82 (m, 2H), 1.41–1.37 (m, 2H), 1.28–1.22 (m, 5H), 1.20–1.15 (m, 2H), 1.14–1.08 (m, 2H), 1.05–0.97 (m, 8H), 0.92–0.85 (m, 6H). *Note*: The quantity of *N*‐ethyl azastatin obtained was insufficient for characterization by ^13^C‐NMR. The **MS**‐fragmentation pattern of the peptide (see SI) unambiguously established the regiochemistry of the ethylation observed in the final deprotection step.

### *In vitro* cytotoxicity evaluation

MMAE and MMAF were purchased from Combi‐Blocks (US). Doxorubicin hydrochloride was obtained from Sigma‐Aldrich/Merck (Germany). HepG2 cells (human hepatocellular carcinoma cells, ATCC), were grown in Eagle's Minimum Essential Medium (ATCC). RPMI 8226 (multiple myeloma cells, ATCC) were grown in complete RPMI medium (ATCC). HCT116 cells (colon carcinoma cells, Anticancer Inc.) were cultured in McCoy′s 5 A Modified Medium (Sigma‐Aldrich). All media were supplemented with 10 % foetal bovine serum, penicillin/streptomycin (100 U/100 μg/mL) and 2 mM L‐glutamine. Cells were seeded in 384‐well assay plates at a density of 1000 cells/well. The cells were incubated over night at 37 °C and test compounds were added, followed incubation for an additional 72 h at 37 °C. The fraction of surviving cells was analysed by a fluorometric microculture cytotoxicity assay, based on hydrolysis of fluorescein diacetate to fluorescein by cells with intact plasma membranes, as recently described.^[49]^ IC_50_‐values were calculated from three independent experiments (see SI, mean±SEM). All tested concentrations were assessed in multiple wells (n=2‐4) in each experiment. The IC_50_‐values were determined with nonlinear regression analysis using GraphPad Prism 8 Software Package (US).

## Conflict of interest

The authors declare no conflict of interest.

## Supporting information

As a service to our authors and readers, this journal provides supporting information supplied by the authors. Such materials are peer reviewed and may be re‐organized for online delivery, but are not copy‐edited or typeset. Technical support issues arising from supporting information (other than missing files) should be addressed to the authors.

Supplementary
