# Supplementary material for: Rational Design of Azastatin as a Potential ADC Payload with Reduced Bystander Killing
Source: ChemMedChem. 2020 Oct 16;15(24):2500–12. doi: 10.1002/cmdc.202000497 (PMC7756782; doi:10.1002/cmdc.202000497)

ChemMedChem

Supporting Information

Rational Design of Azastatin as a Potential ADC Payload with Reduced Bystander Killing

Rafael W. Hartmann, Raphael Fahrner, Denys Shevshenko, Mårten Fyrknäs, Rolf Larsson, Fredrik Lehmann, and Luke R. Odell*

Contents

I. Evaluation of Apparent Cytotoxicity *in vitro* 2

II. NMR spectra: Synthesis of N-terminal tripeptide 3

*N*Cbz-β-keto-γ-amino acid *tert*-butyl ester **9a** 3

*N*Cbz-Dolaisoleucine *tert*-butyl ester **8a** 5

Monocylic lactam **18** 7

Cbz*N*-Val-Dil-O*t*Bu **14** 8

Dov-Val-Dil-O*t*Bu **15** 10

III. NMR spectra: Synthesis of C-terminal dipeptide 12

*N*Boc-*O*Ac-L-hydroxyproline pentafluorophenyl ester **20** 12

*N*Boc-*O*Ac-β-keto-γ-amino acid ethyl ester **9b** 15

*N*Boc-*O*Ac-β-hydroxy-γ-amino acid ethyl ester **(*R*,*R*)-22** 17

Bicyclic lactam **(*R*,*R*)-23** 19

Bicyclic lactam **(*S*,*S*)-23** 25

*N*Boc-*O*Ac-β-methoxy-γ-amino acid ethyl ester **8b** 31

*N*Boc-hydroxy-β-methoxy-γ-amino acid ethyl ester 33

*N*Boc-azido-β-methoxy-γ-amino acid ethyl ester **24** 35

*N*Boc-4-(Cbz-amino)Dap-Phe-OMe **26** 37

IV. NMR and MS spectra: Endgame peptide assembly 39

Cbz-azastatin methyl ester **27** 39

Azastatin methyl ester **7** 41

N-ethyl azastatin methyl ester **28** 44

I. Evaluation of Apparent Cytotoxicity *in vitro*

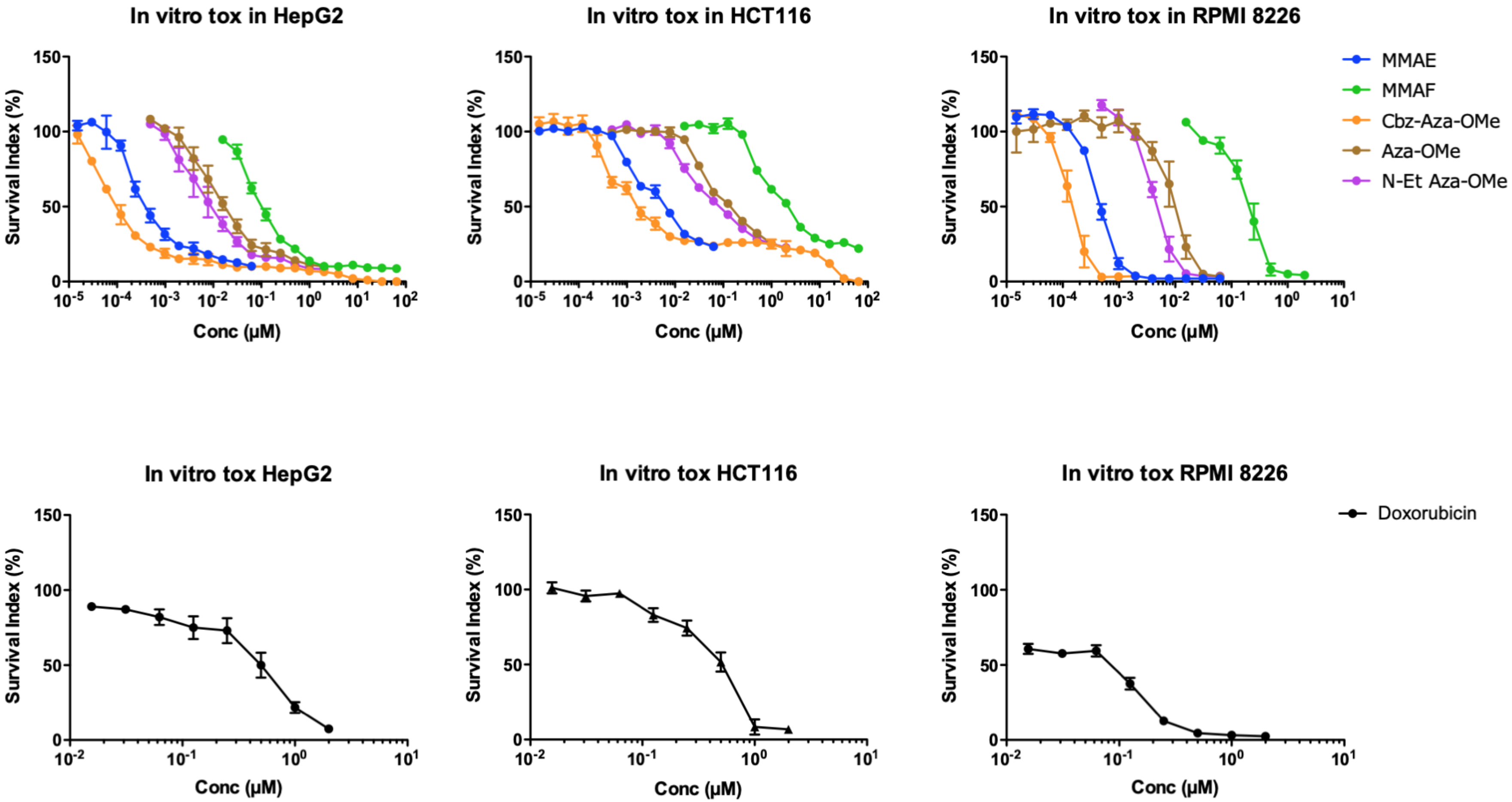

II. NMR spectra: Synthesis of N-terminal tripeptide

*N*Cbz-β-keto-γ-amino acid *tert*-butyl ester **9a**

^{1}H, DMSO-d_6

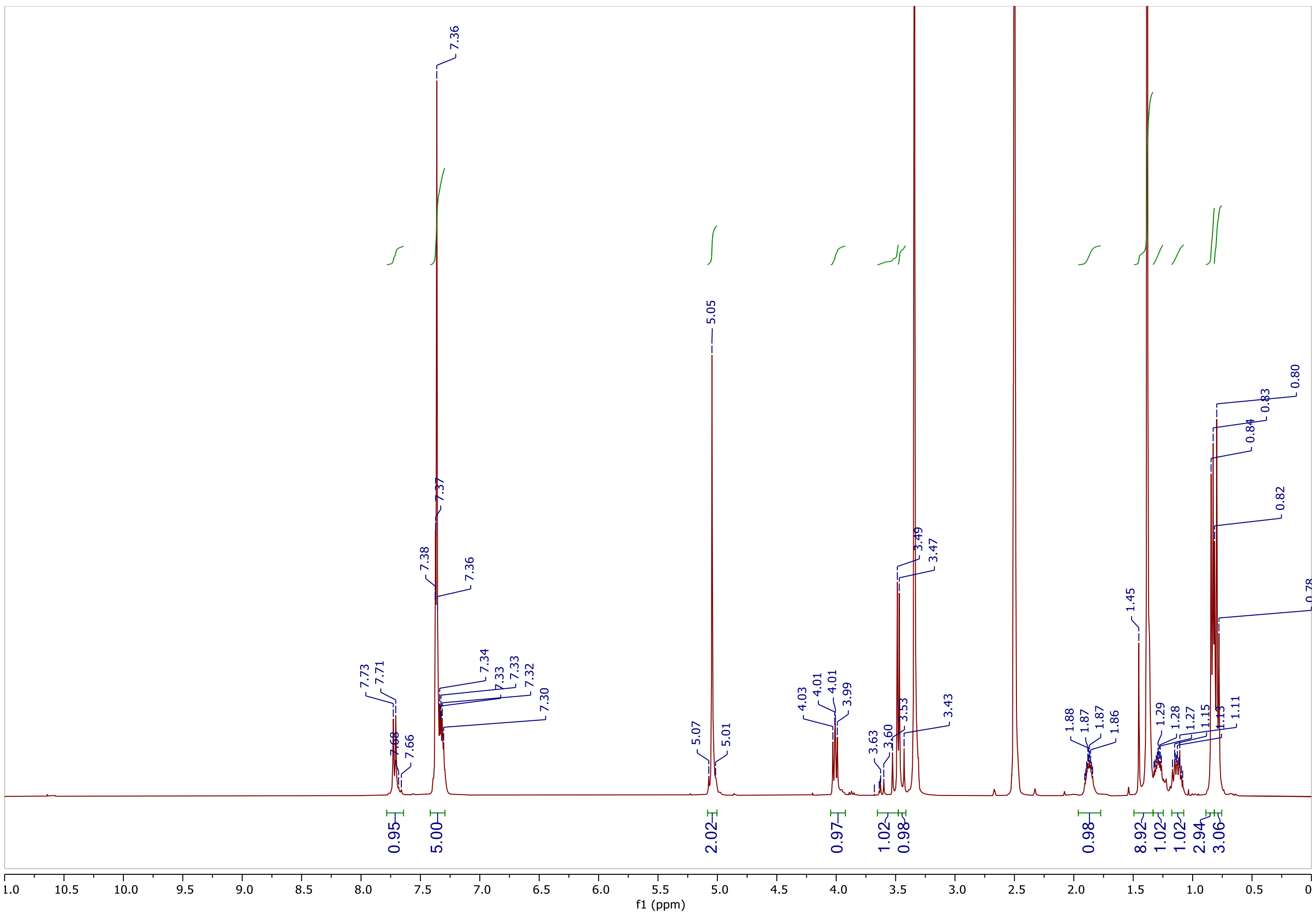

^{13}C, DMSO-d_6

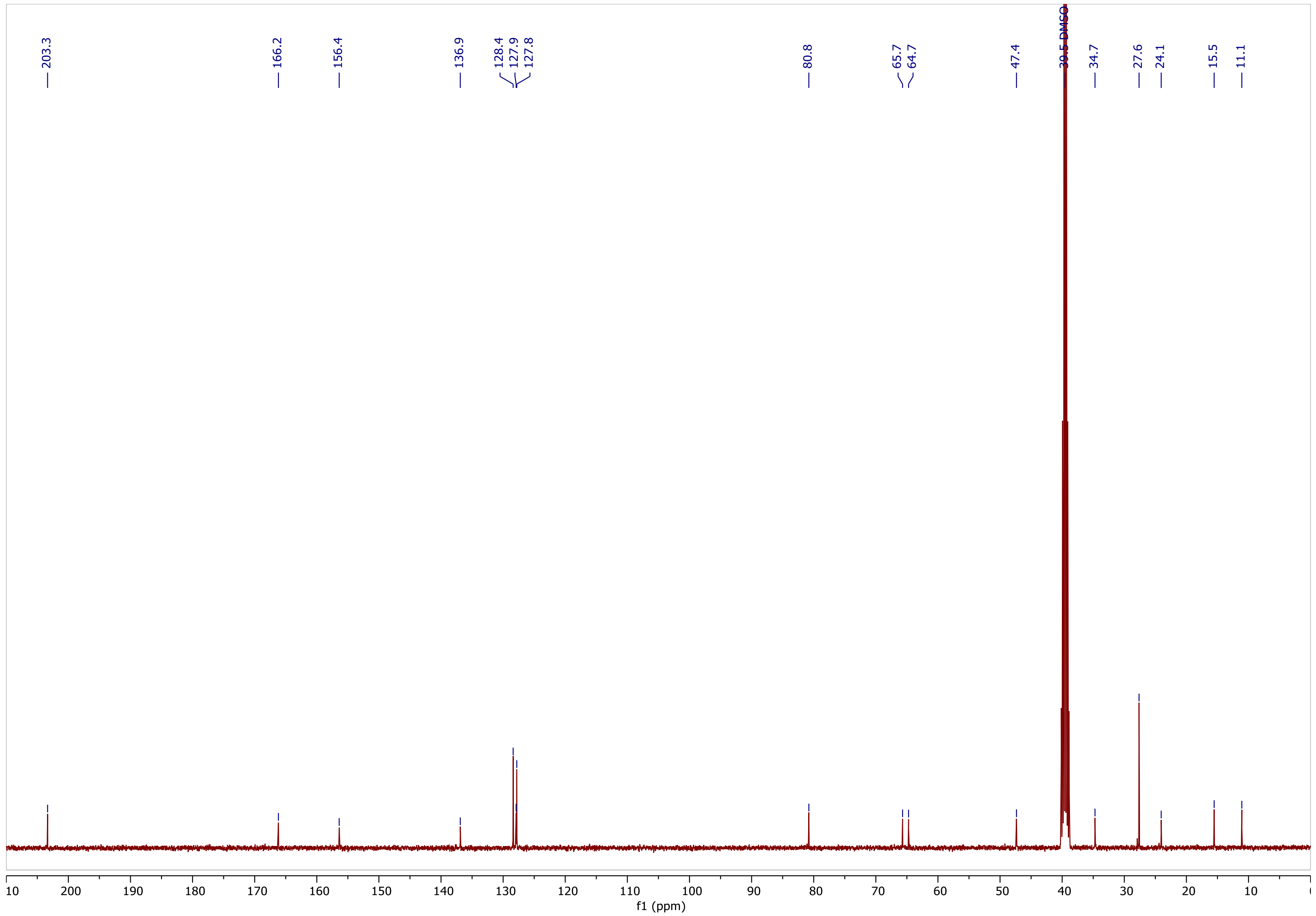
203.3
166.2
156.4
136.9
128.4
127.9
127.8
80.8
65.7
64.7
47.4
39.5 DMSO
34.7
27.6
24.1
15.5
11.1
f1 (ppm)

*N*Cbz-Dolaisoleucine *tert*-butyl ester **8a**

^{1}H, CDCl$_3$

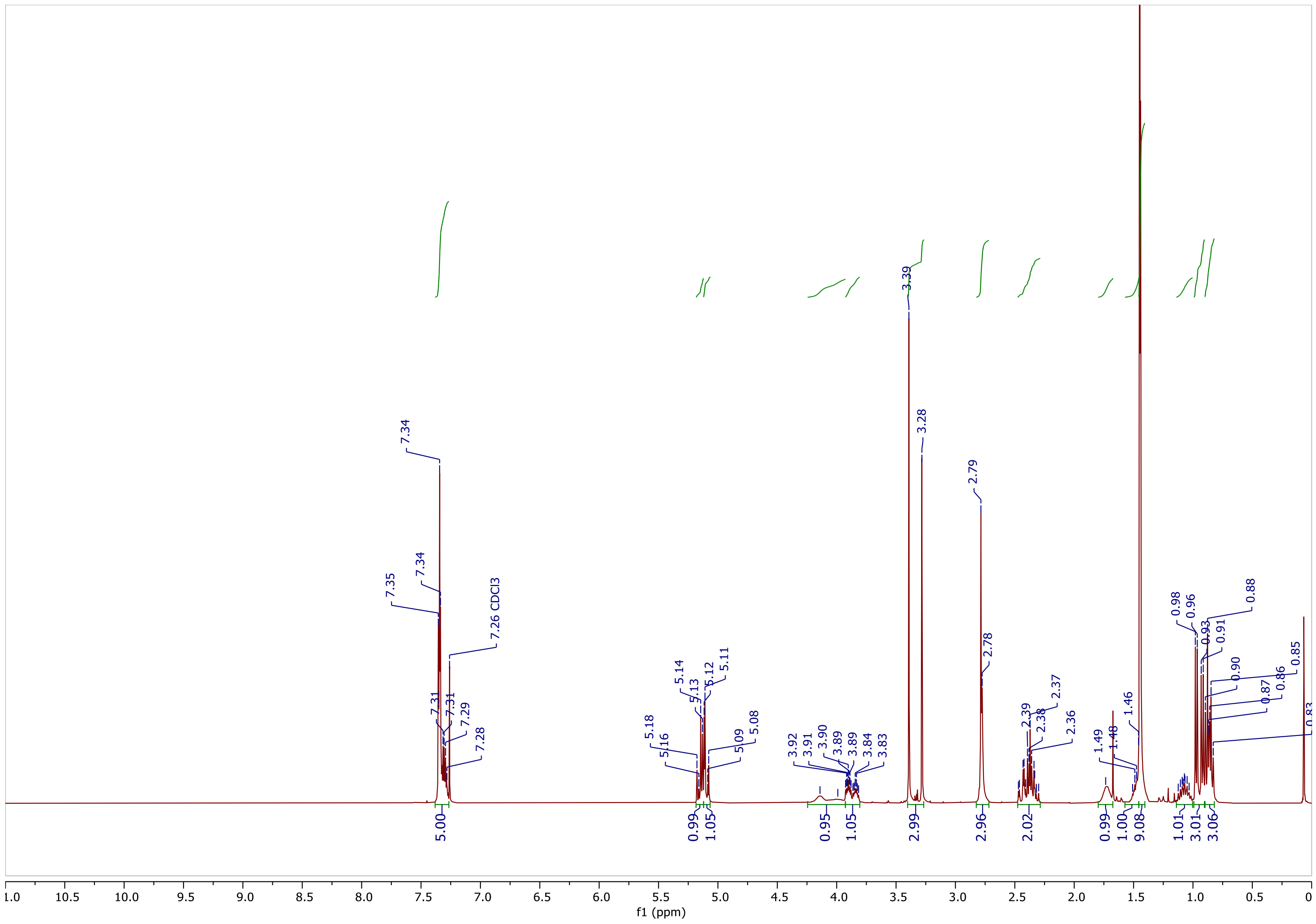

^{13}C, $CDCl_3$

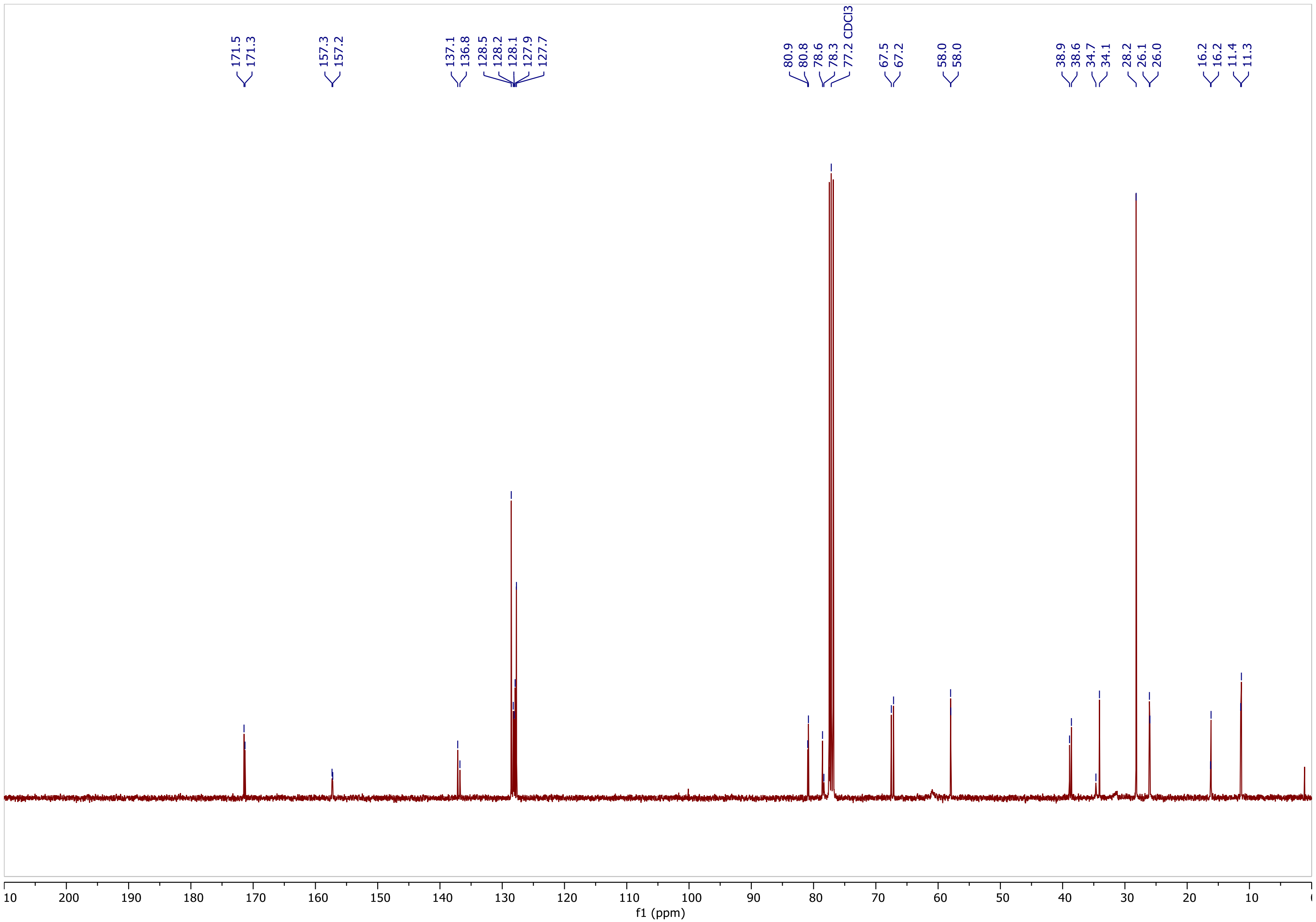

171.5
171.3
157.3
157.2
137.1
136.8
128.5
128.2
128.1
127.9
127.7
80.9
80.8
78.6
78.3
77.2 CDCl3
67.5
67.2
58.0
58.0
38.9
38.6
34.7
34.1
28.2
26.1
26.0
16.2
16.2
11.4
11.3
f1 (ppm)

Monocylic lactam **18**

^{1}H, $CDCl_3$

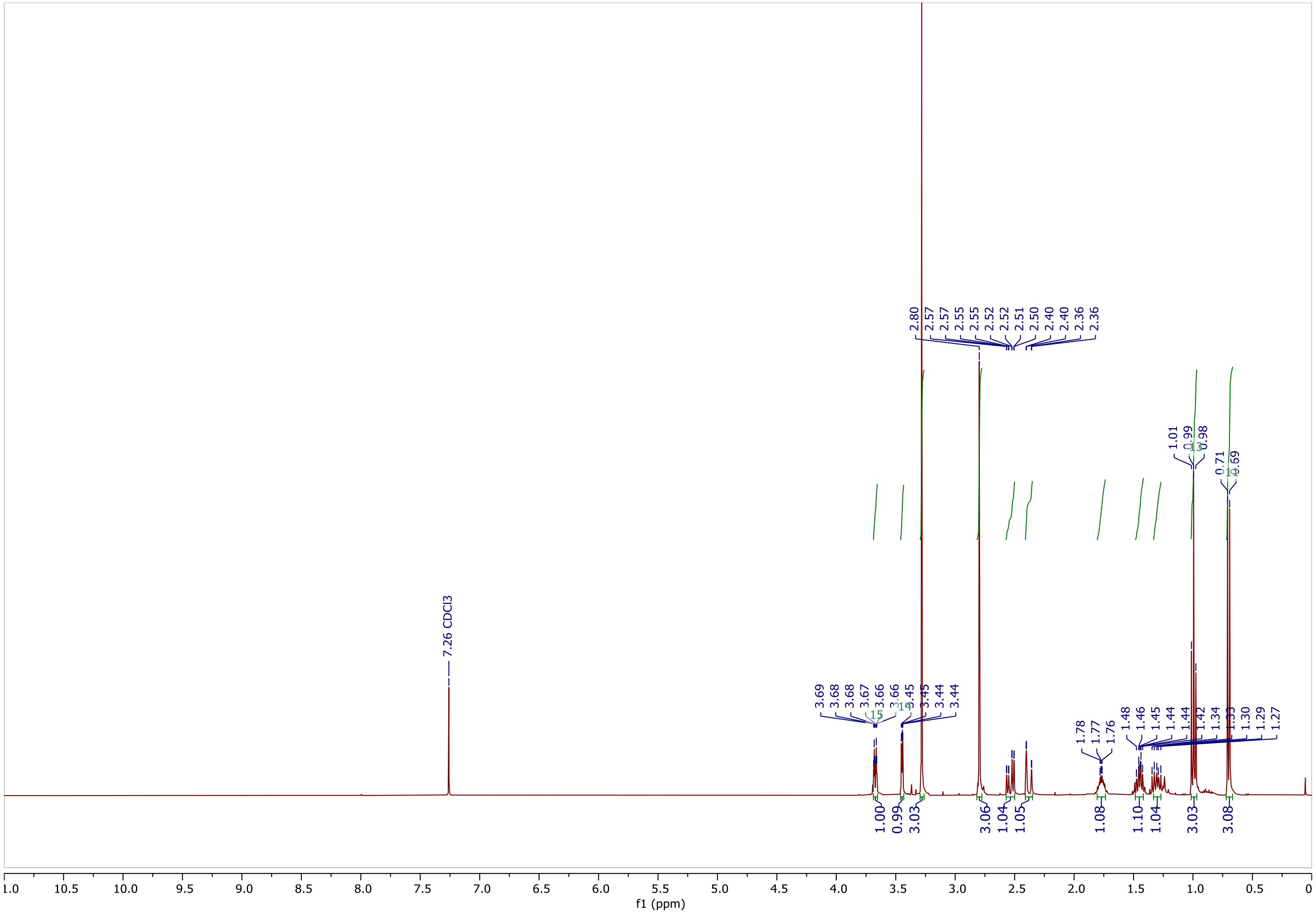

CbzN-Val-Dil-OtBu **14**

^{1}H, $CDCl_3$

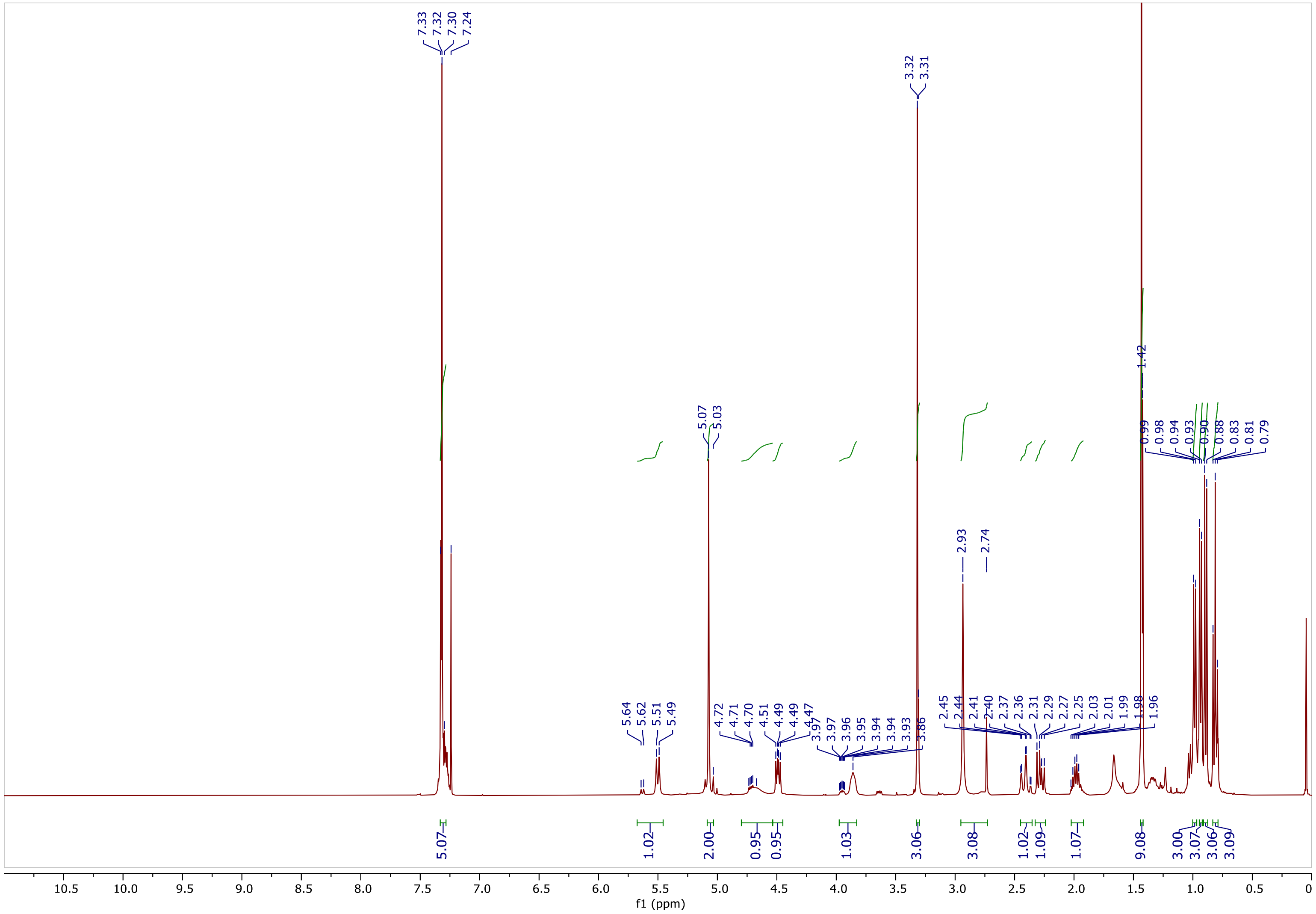

^{13}C, $CDCl_3$

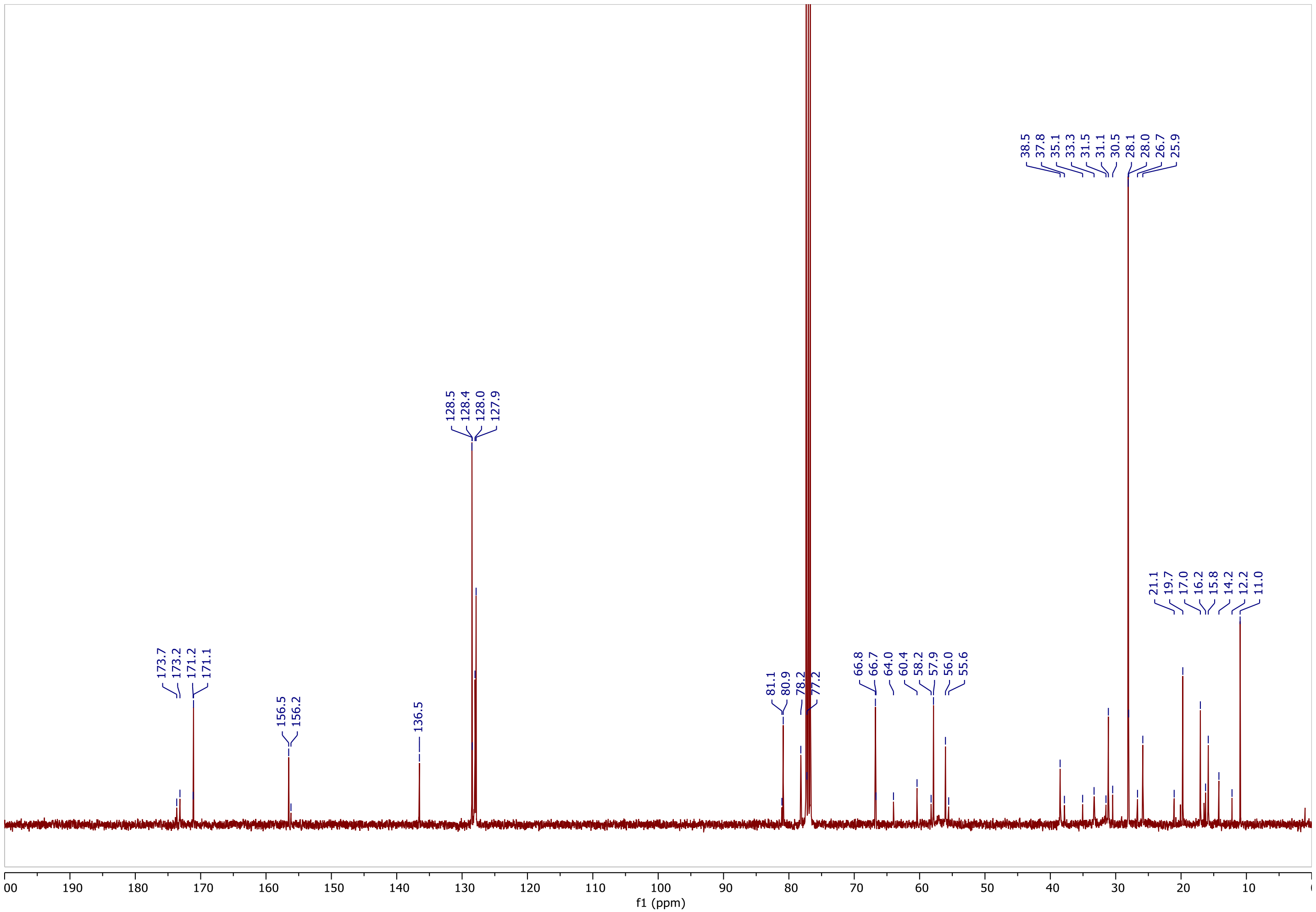
173.7
173.2
171.2
171.1
156.5
156.2
136.5
128.5
128.4
128.0
127.9
81.1
80.9
78.2
77.2
66.8
66.7
64.0
60.4
58.2
57.9
56.0
55.6
38.5
37.8
35.1
33.3
31.5
31.1
30.5
28.1
28.0
26.7
25.9
21.1
19.7
17.0
16.2
15.8
14.2
12.2
11.0
00
190
180
170
160
150
140
130
120
110
100
90
80
70
60
50
40
30
20
10
f1 (ppm)

Dov-Val-Dil-O*t*Bu **15**

^{1}H, $CDCl_3$

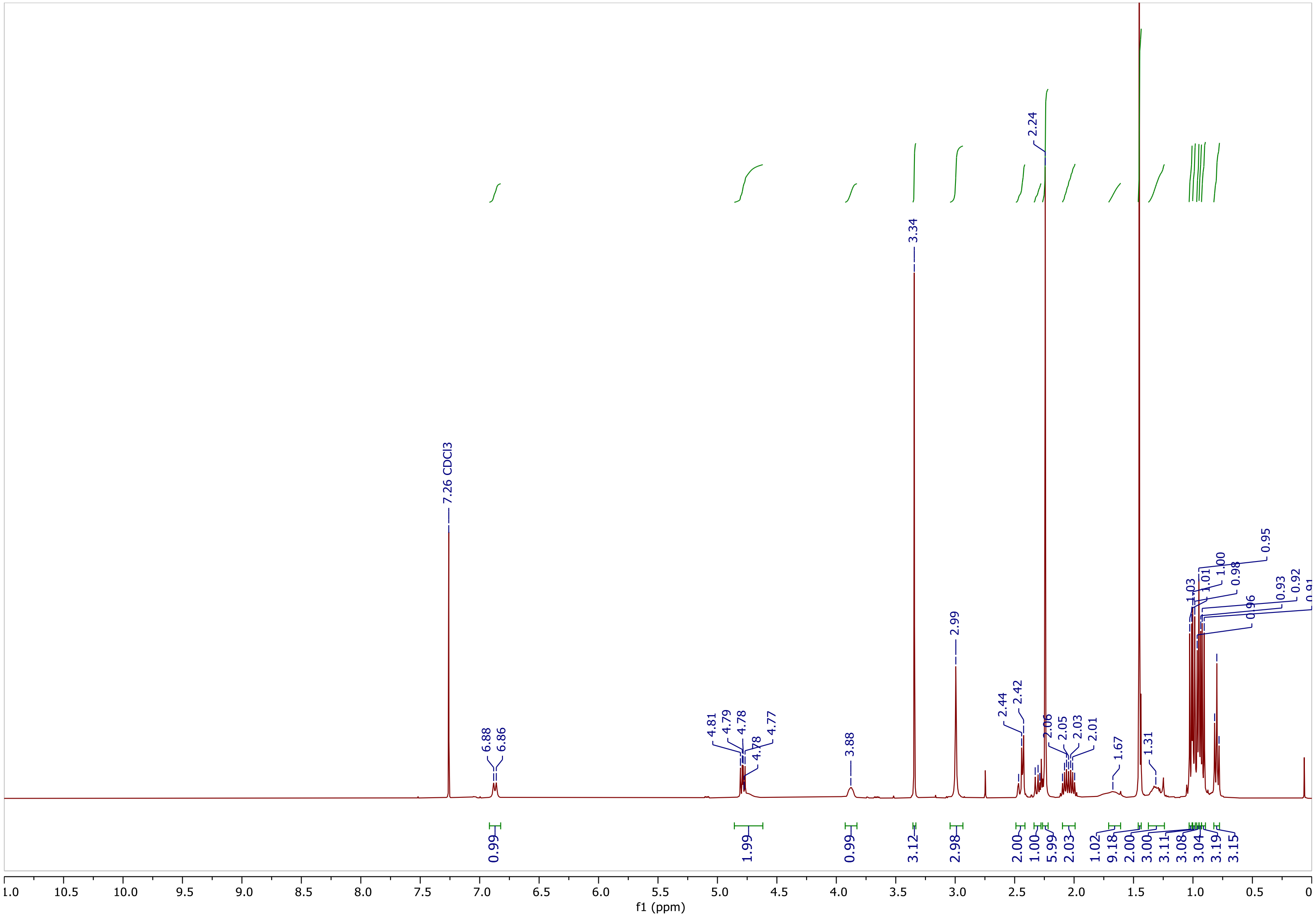

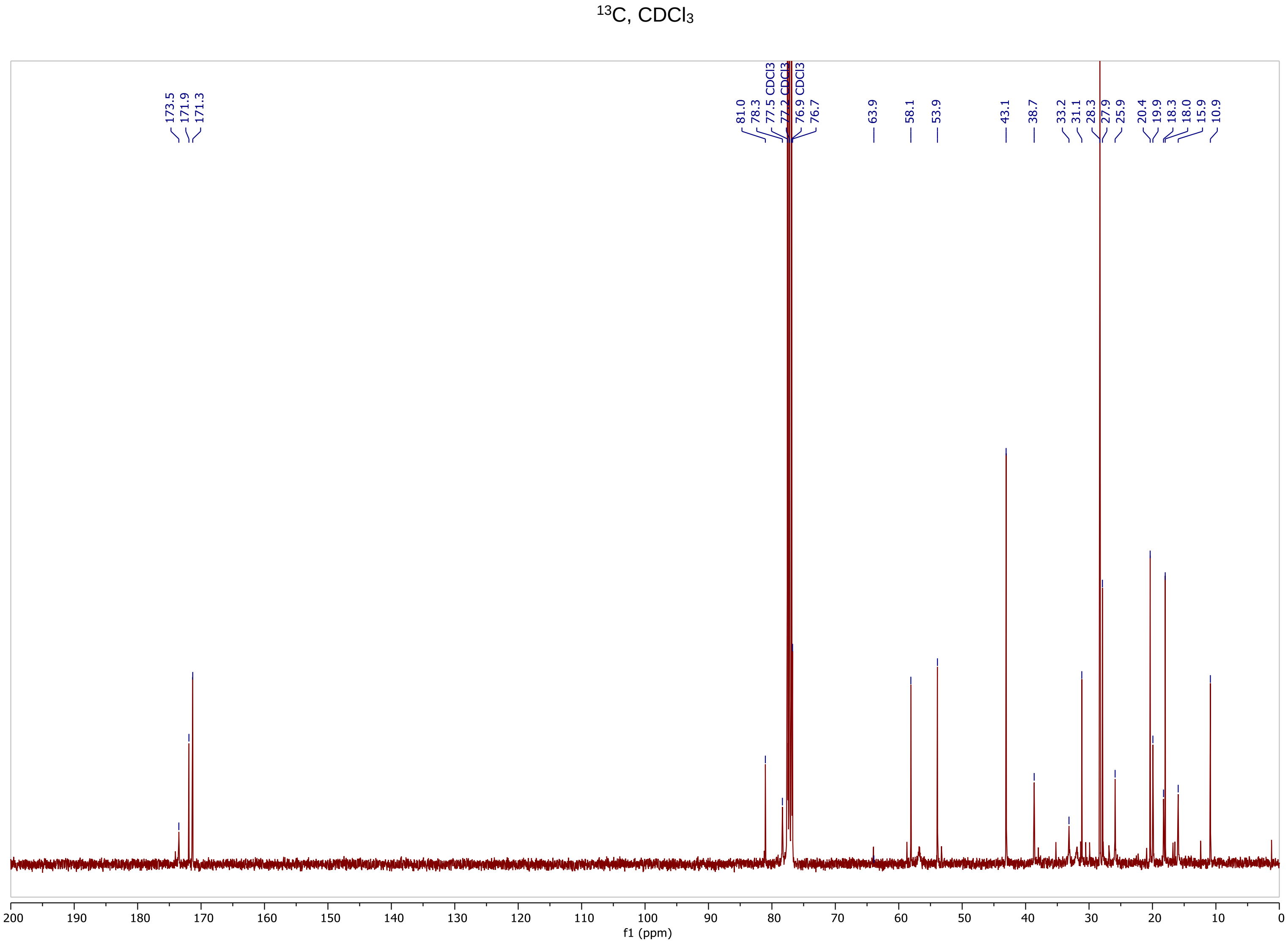

13C, CDCl3
173.5
171.9
171.3
81.0
78.3
77.5 CDCl3
77.2 CDCl3
76.9 CDCl3
76.7
63.9
58.1
53.9
43.1
38.7
33.2
31.1
28.3
27.9
25.9
20.4
19.9
18.3
18.0
15.9
10.9
f1 (ppm)

III. NMR spectra: Synthesis of C-terminal dipeptide

*N*Boc-*O*Ac-L-hydroxyproline pentafluorophenyl ester **20**

[1]H, $CDCl_3$

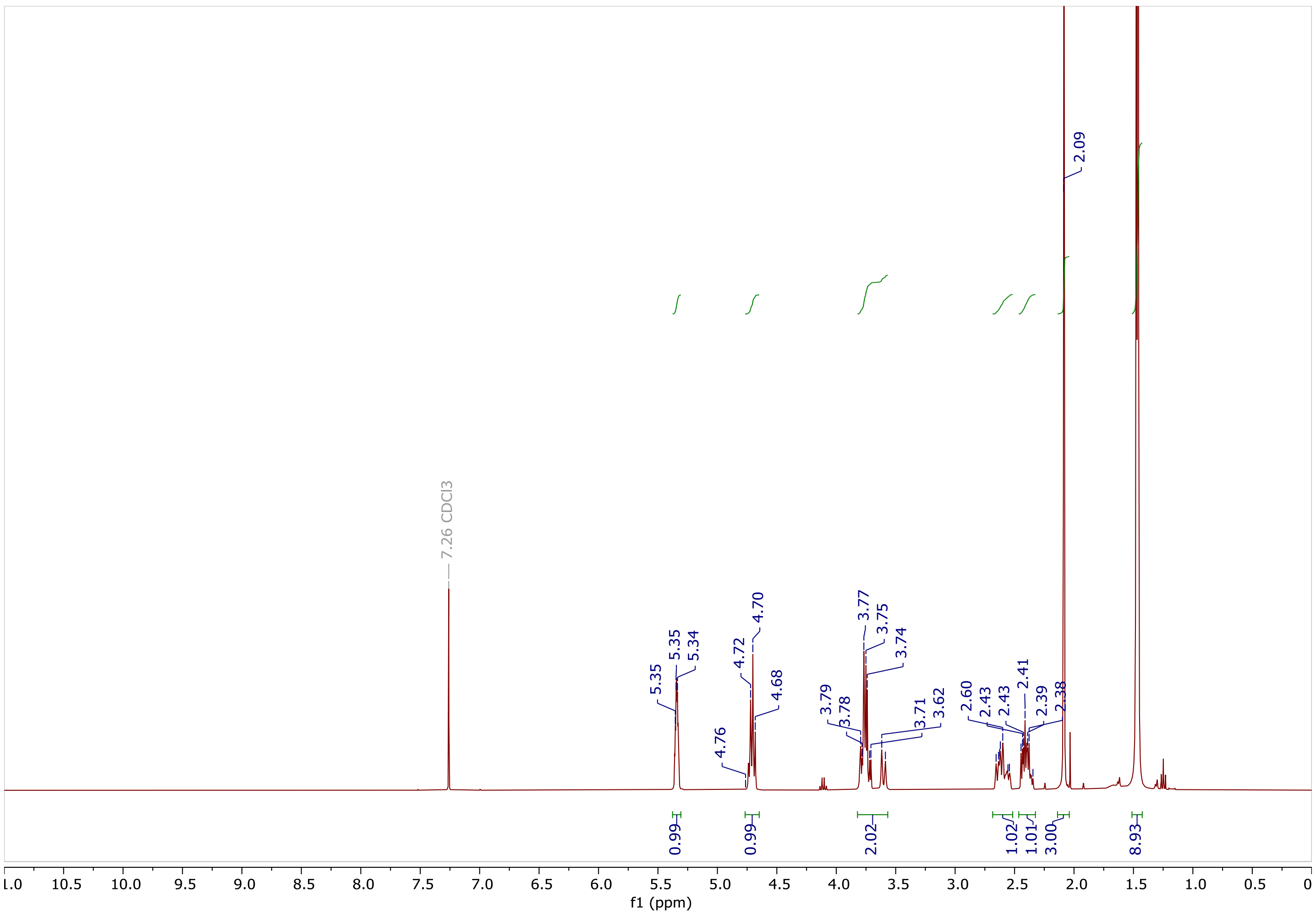

^{19}F, $CDCl_3$

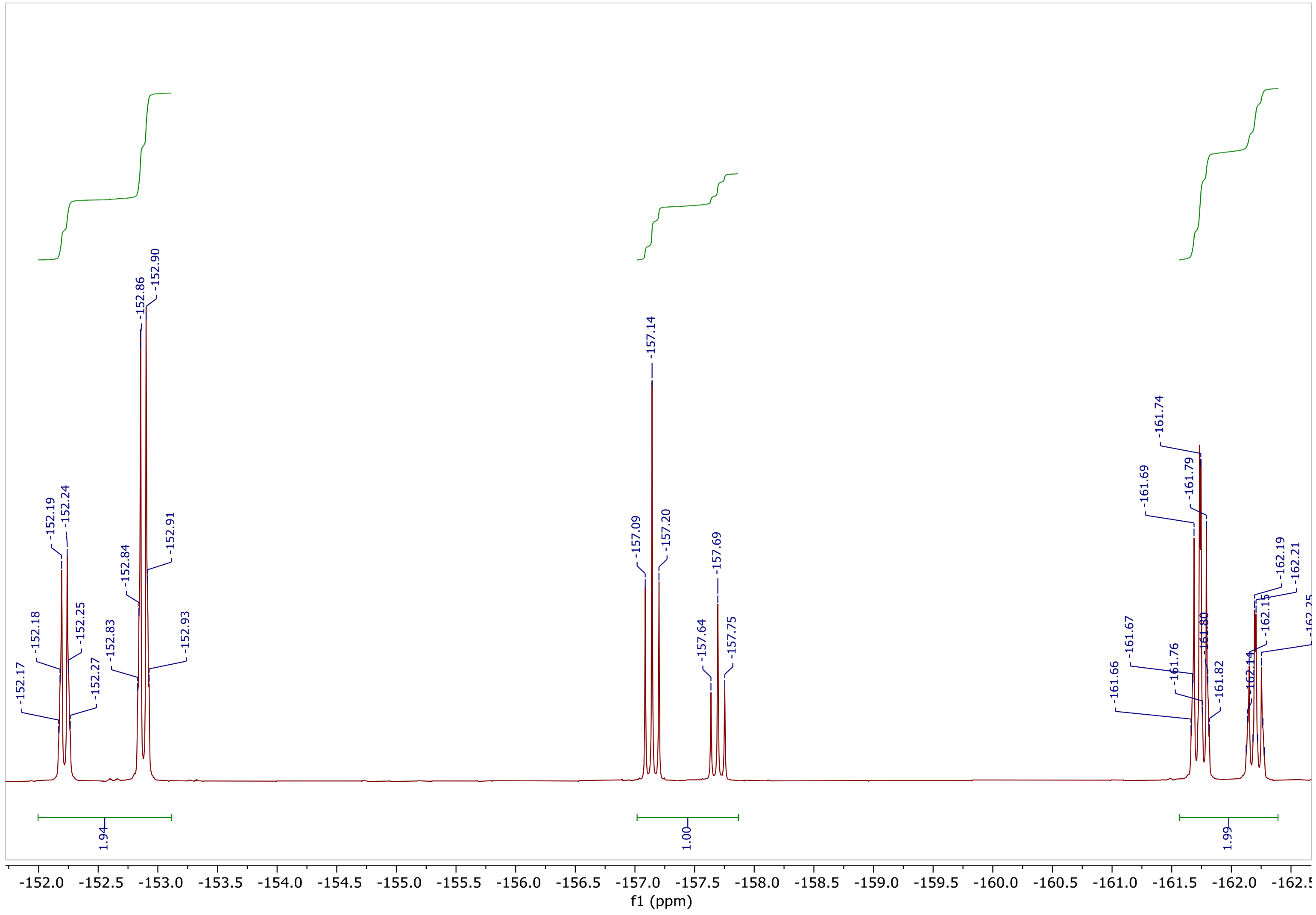

-152.17
-152.18
-152.19
-152.24
-152.25
-152.27
-152.83
-152.84
-152.86
-152.90
-152.91
-152.93
-157.09
-157.14
-157.20
-157.64
-157.69
-157.75
-161.66
-161.67
-161.69
-161.74
-161.76
-161.79
-161.80
-161.82
-162.14
-162.15
-162.19
-162.21
1.94
1.00
1.99
f1 (ppm)

^{13}C, $CDCl_3$

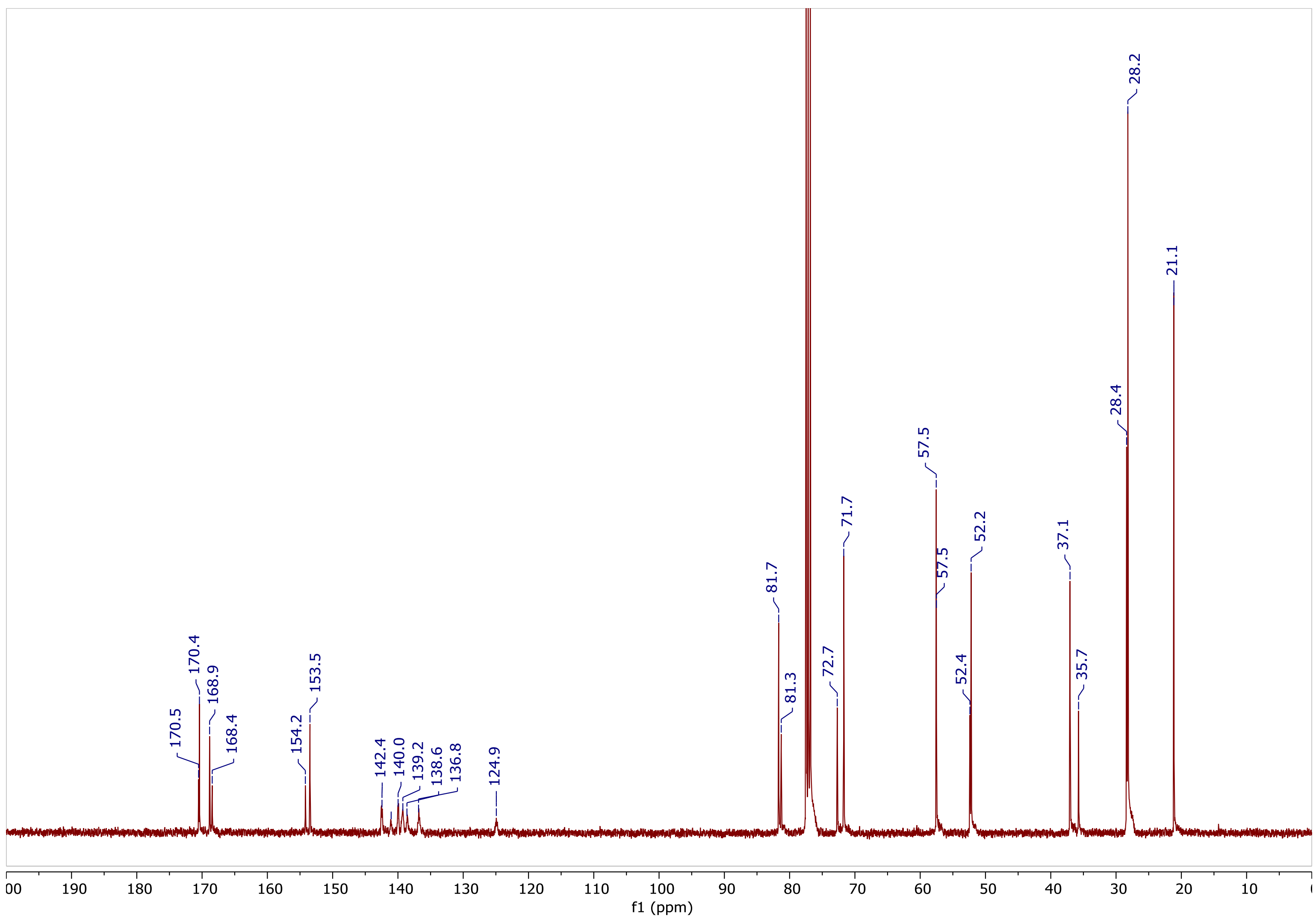

170.5
170.4
168.9
168.4
154.2
153.5
142.4
140.0
139.2
138.6
136.8
124.9
81.7
81.3
72.7
71.7
57.5
57.5
52.4
52.2
37.1
35.7
28.4
28.2
21.1
200
190
180
170
160
150
140
130
120
110
100
90
80
70
60
50
40
30
20
10
0
f1 (ppm)

*N*Boc-*O*Ac-β-keto-γ-amino acid ethyl ester **9b**

^{1}H, CD_3CN

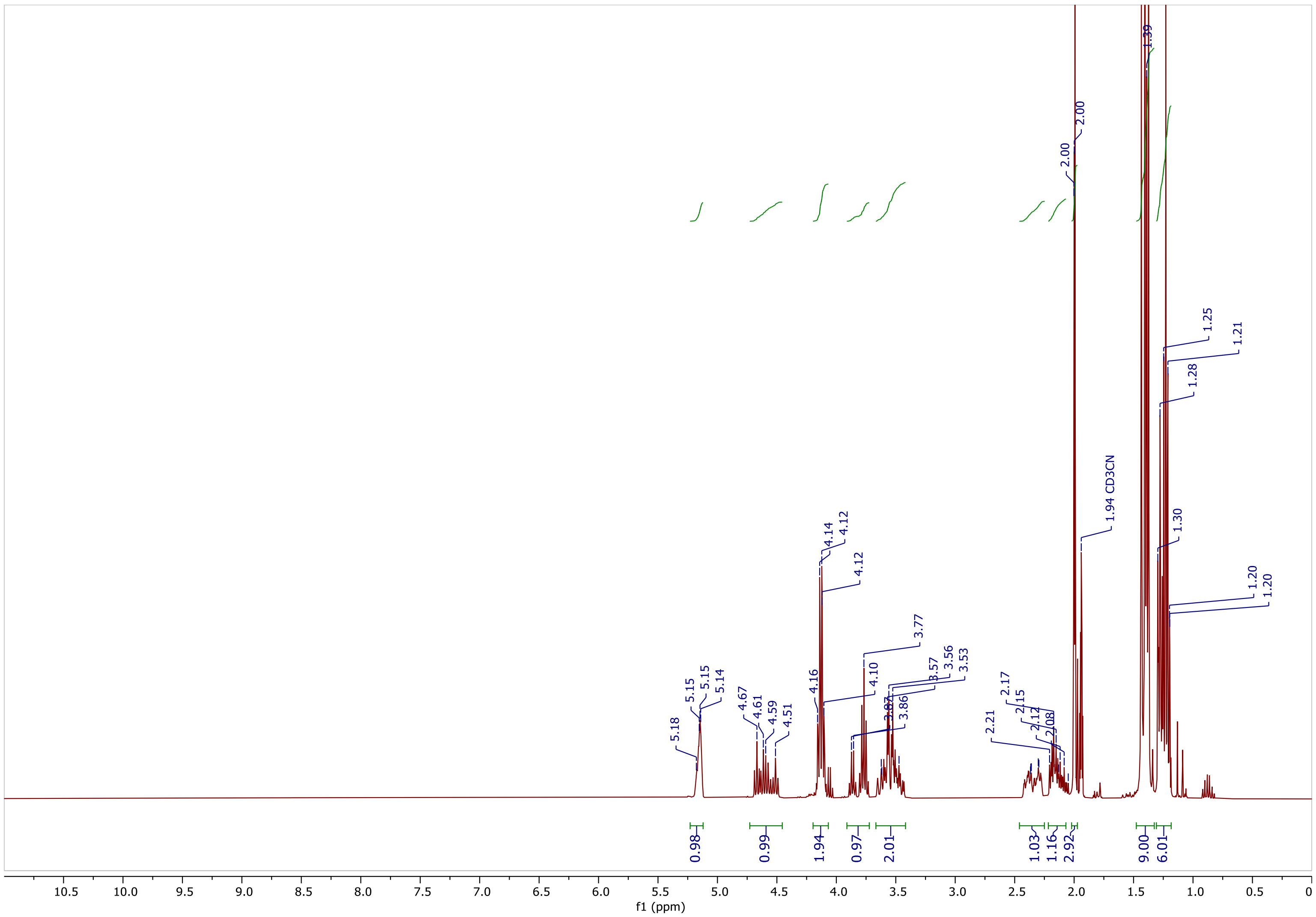

^{13}C, CD_3CN

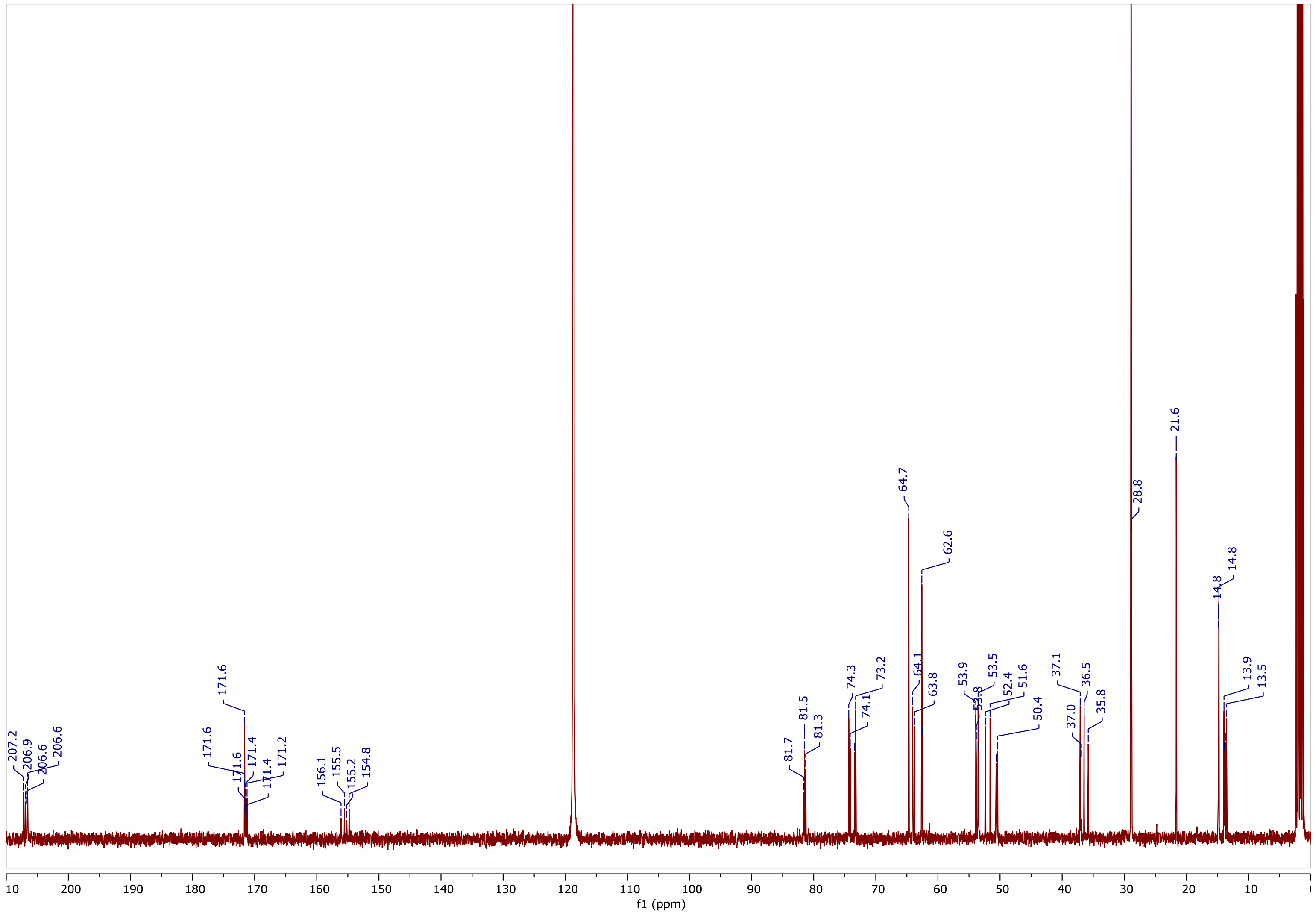
207.2
206.9
206.6
206.6
171.6
171.6
171.6
171.4
171.4
171.2
156.1
155.5
155.2
154.8
81.7
81.5
81.3
74.3
74.1
73.2
64.7
64.1
63.8
62.6
53.9
53.8
53.5
52.4
51.6
50.4
37.1
37.0
36.5
35.8
28.8
21.6
14.8
14.8
13.9
13.5
200
190
180
170
160
150
140
130
120
110
100
90
80
70
60
50
40
30
20
10
f1 (ppm)

*N*Boc-*O*Ac-β-hydroxy-γ-amino acid ethyl ester **(*R*,*R*)-22**

^{1}H, CD_3CN

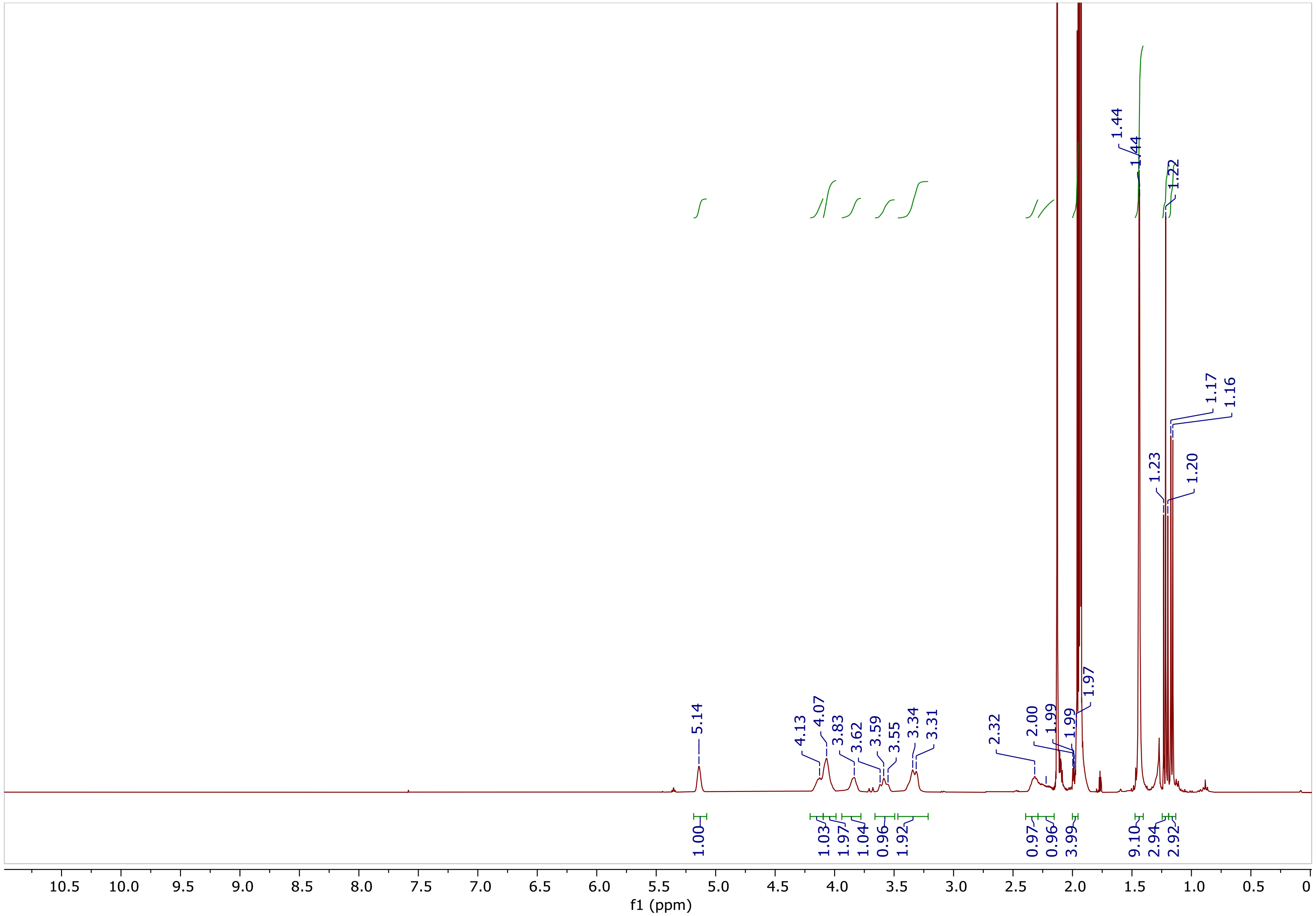

^{13}C, CD_3CN

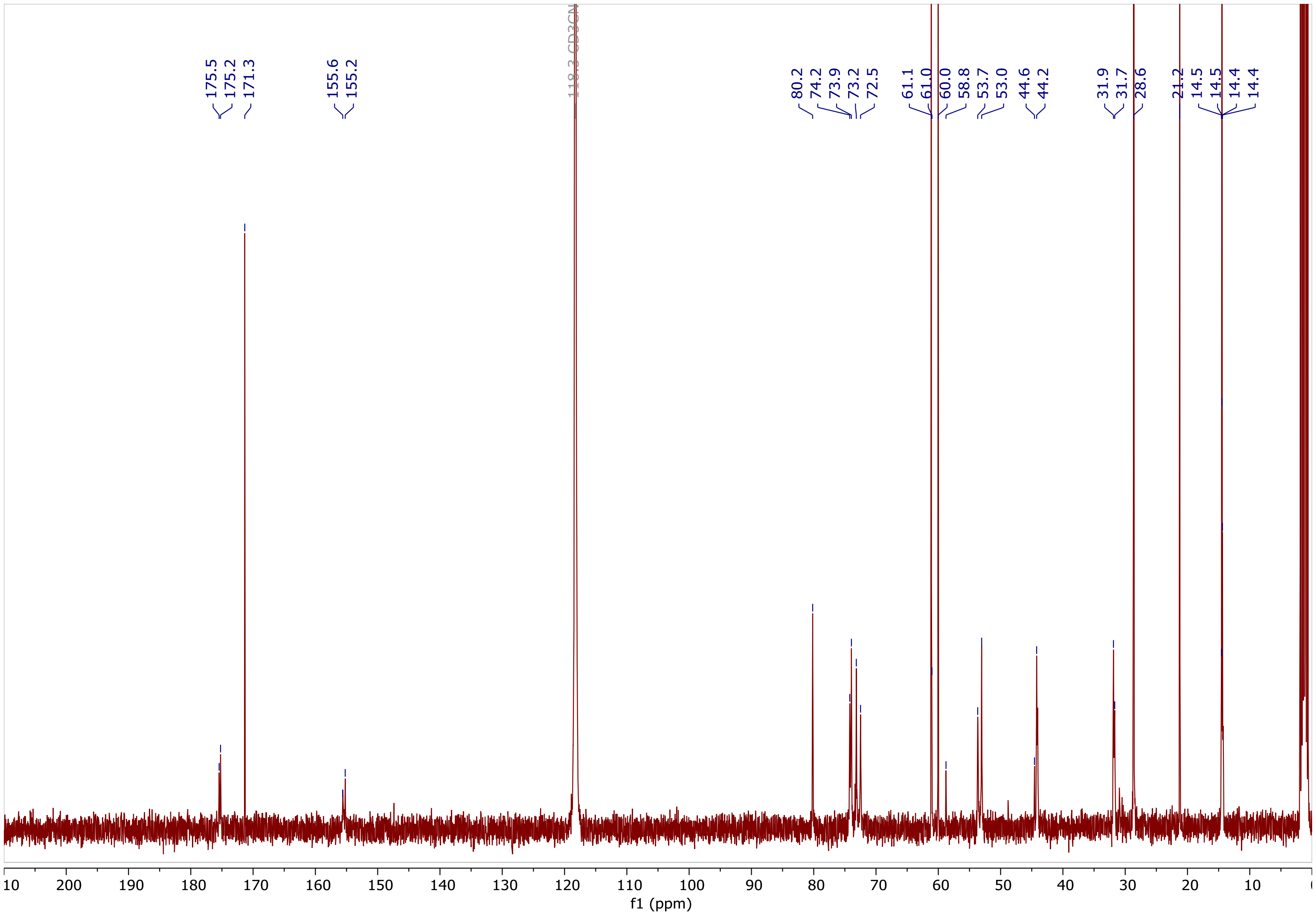
175.5
175.2
171.3
155.6
155.2
118.3 CD3CN
80.2
74.2
73.9
73.2
72.5
61.1
61.0
60.0
58.8
53.7
53.0
44.6
44.2
31.9
31.7
28.6
21.2
14.5
14.5
14.4
14.4
10
200
190
180
170
160
150
140
130
120
110
100
90
80
70
60
50
40
30
20
10
f1 (ppm)

Bicyclic lactam **(*R*,*R*)-23**[1]

^{1}H, DMSO-d_6

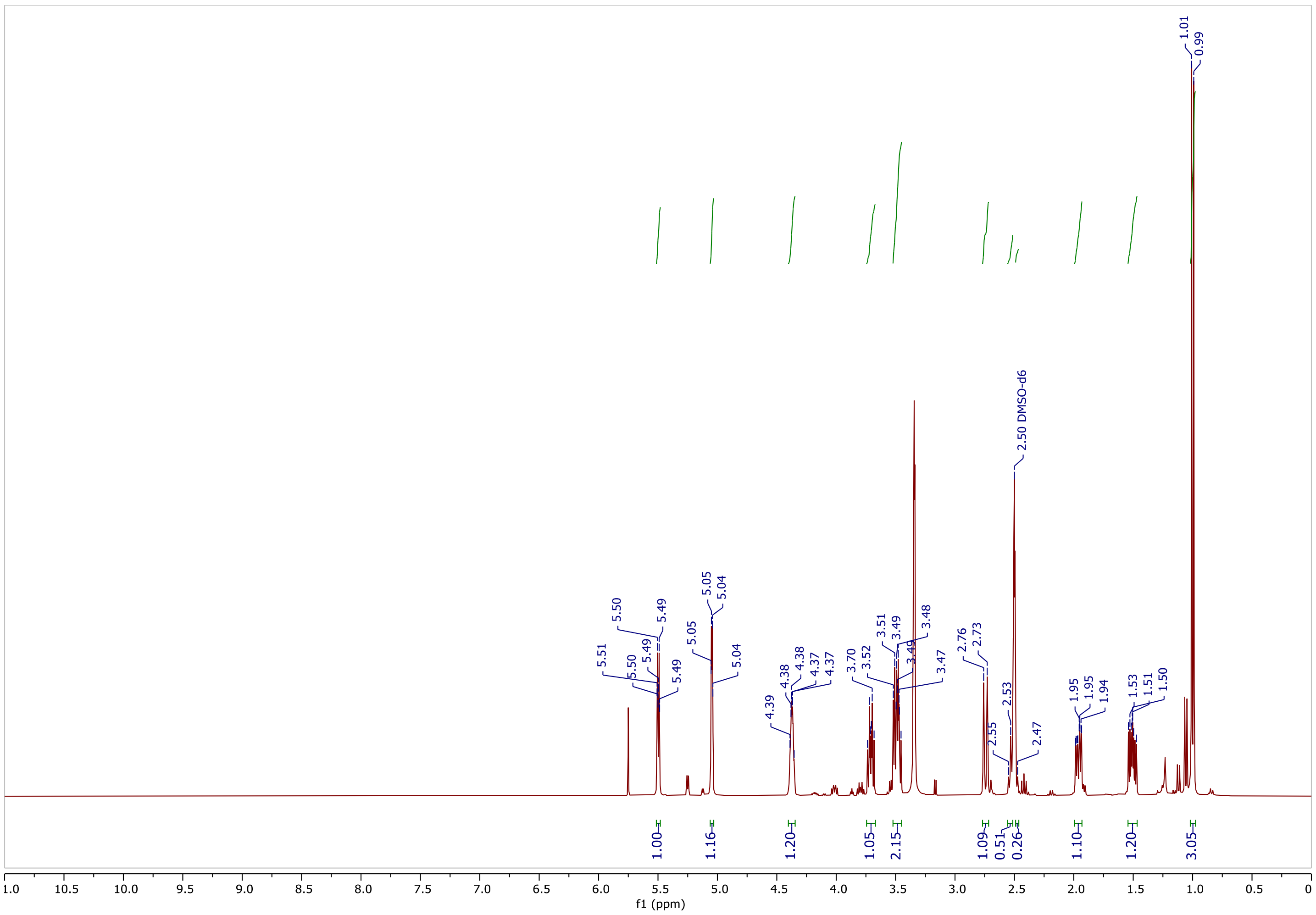

[1] Contaminated with traces of another diastereomer (see Scheme 2A in core document)

^{1}H, DMSO-d_6 (zoomed in)

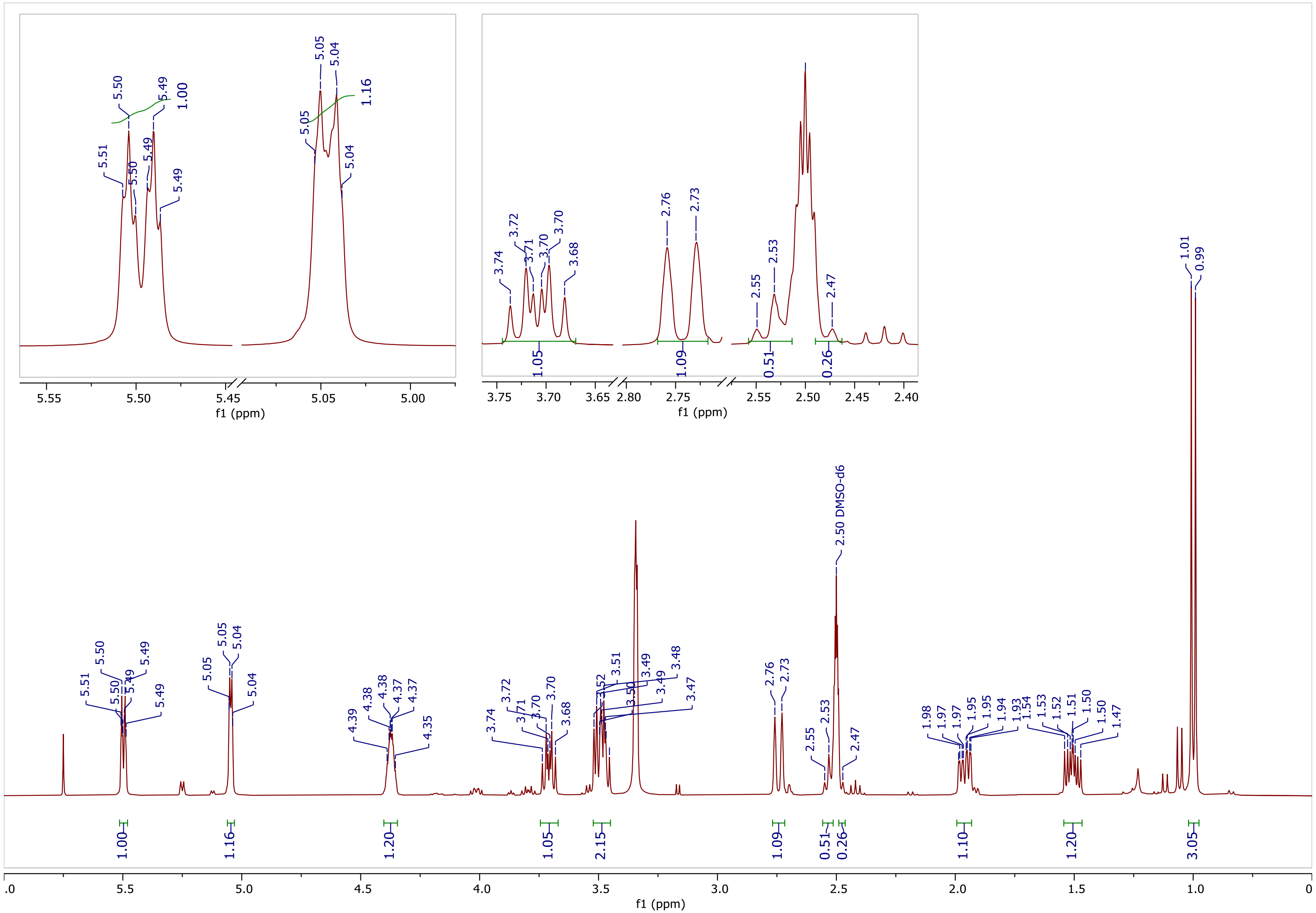

^{13}C, DMSO-d_6

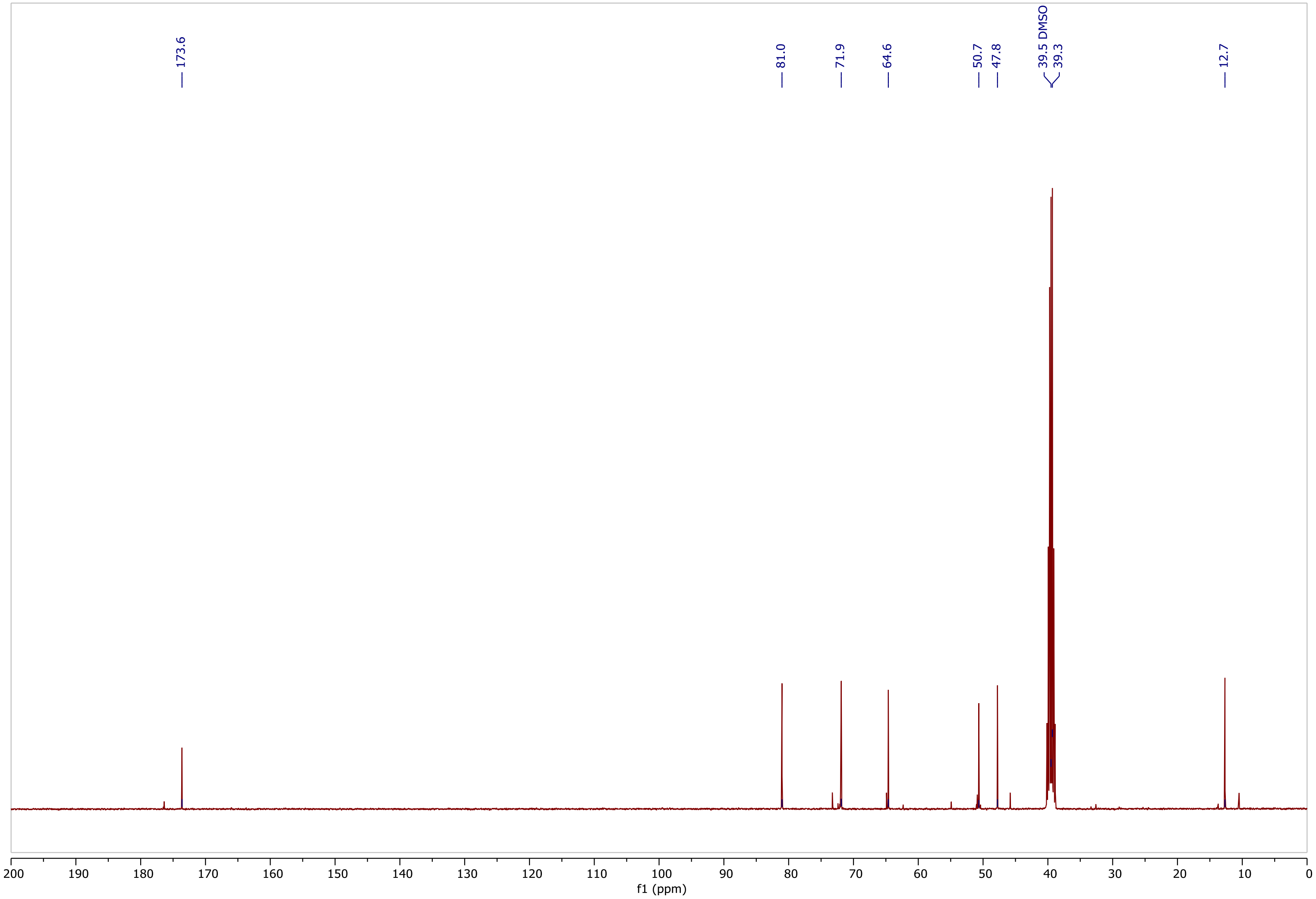
173.6
81.0
71.9
64.6
50.7
47.8
39.5 DMSO
39.3
12.7
200 190 180 170 160 150 140 130 120 110 100 90 80 70 60 50 40 30 20 10 0
f1 (ppm)

(¹H-¹H)-COSY, DMSO-d6

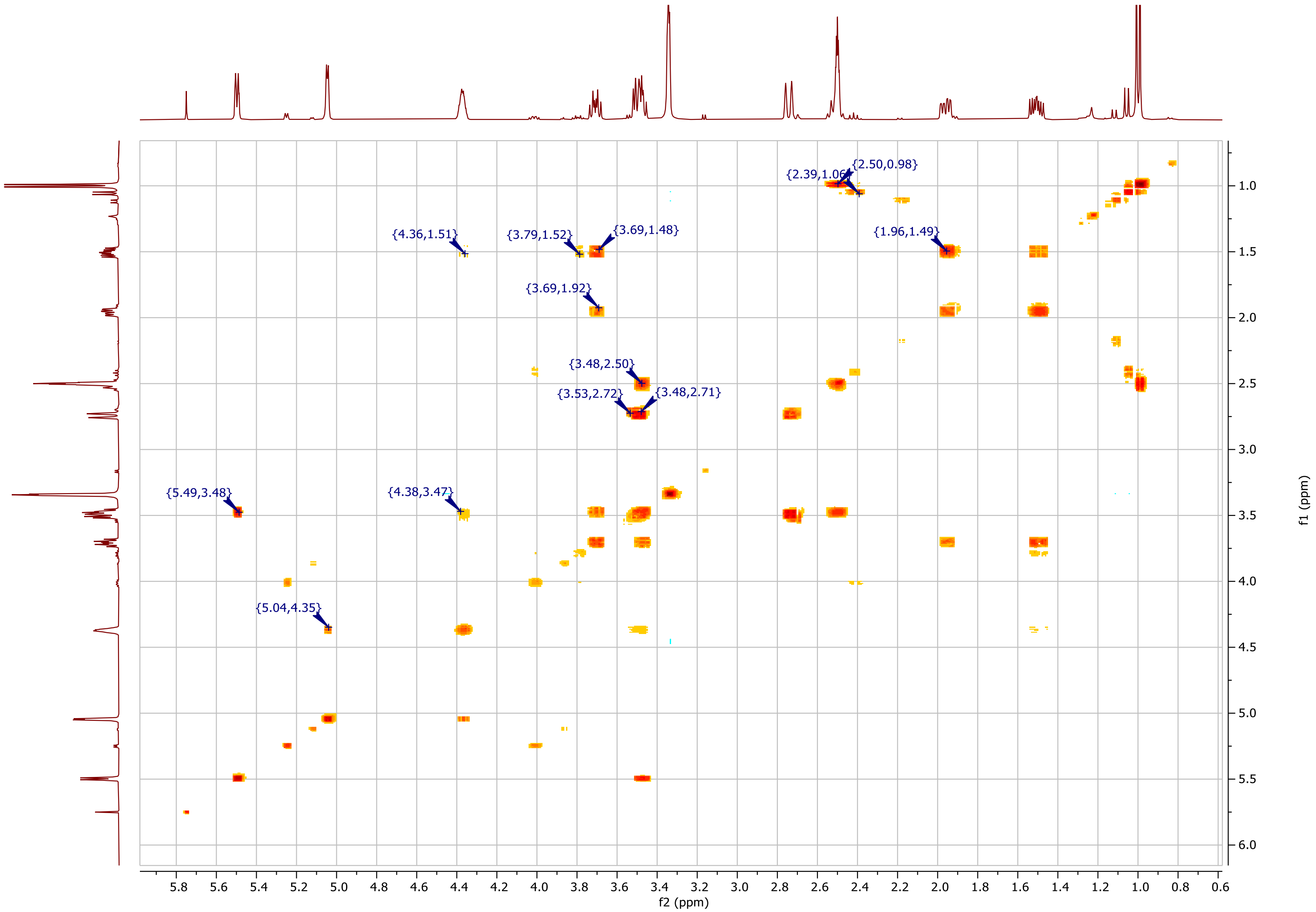

(^{1}H-^{13}C)-HSQC, DMSO-d6

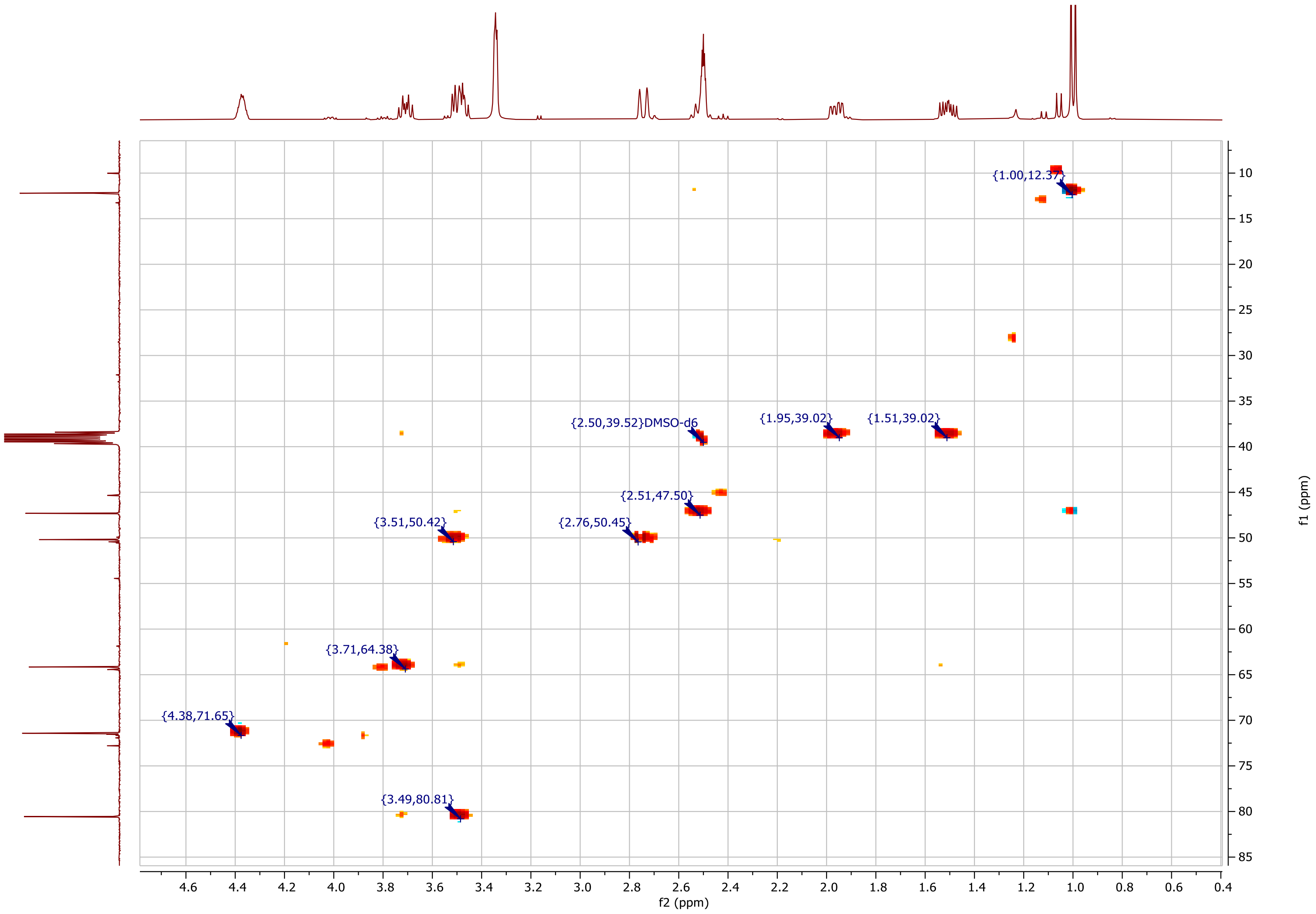

(^{1}H-^{1}H)-NOESY, DMSO-d$_6$

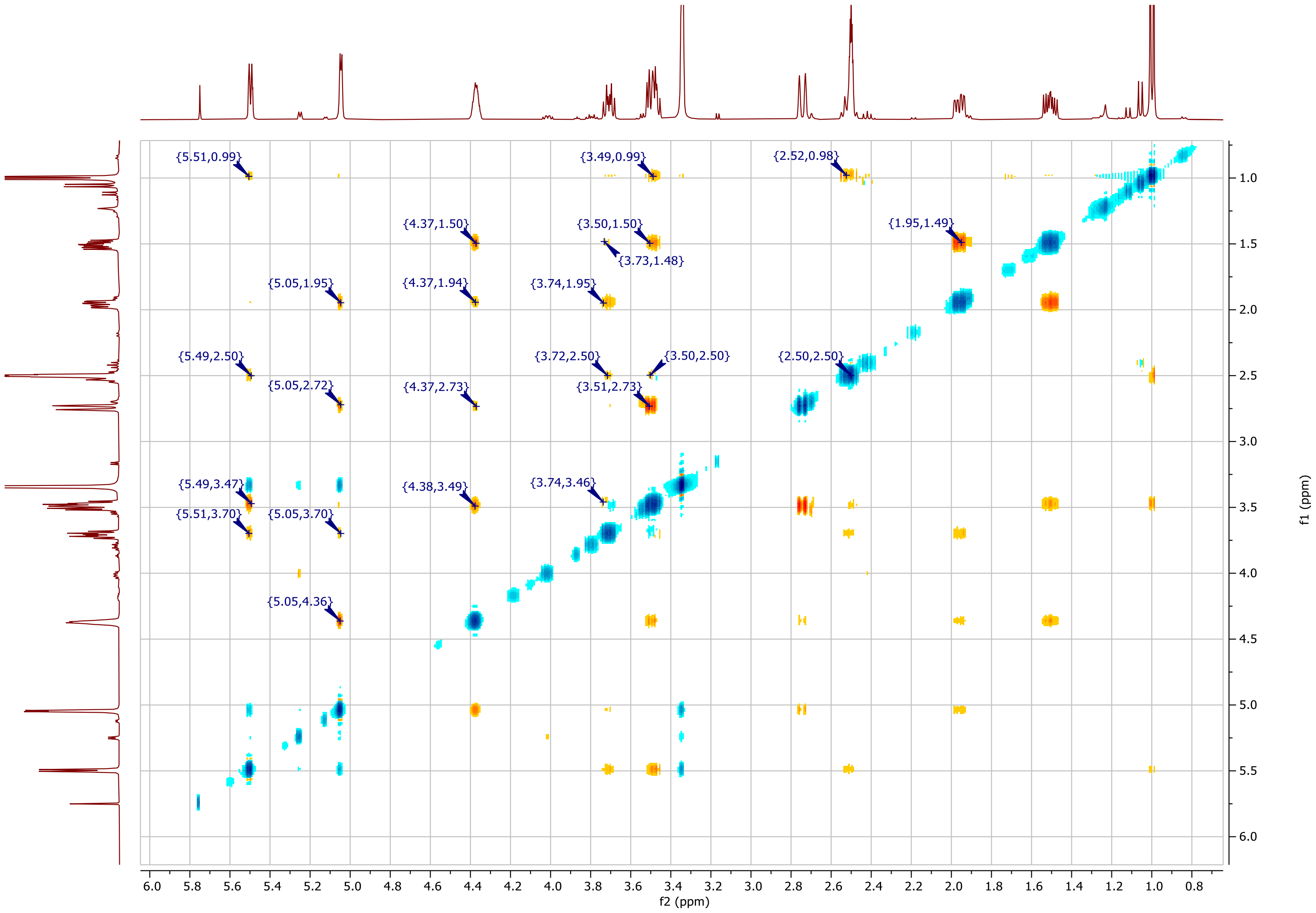

Bicyclic lactam **(*S*,*S*)-23**[2]

^{1}H, DMSO-d6

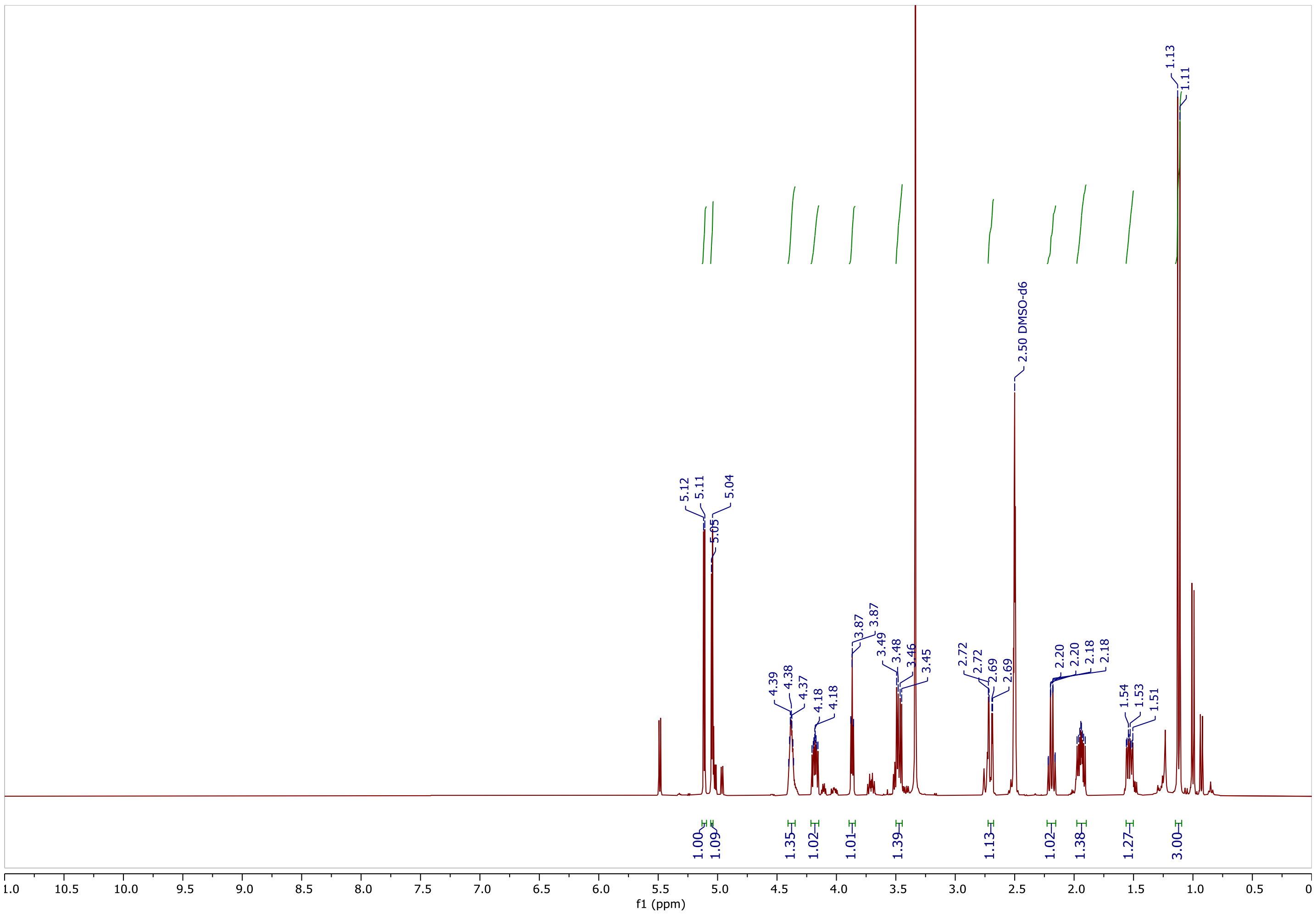

[2] Contaminated with traces of another diastereomer (see Scheme 2A in core document)

[1]H, DMSO-d6 (zoomed in)

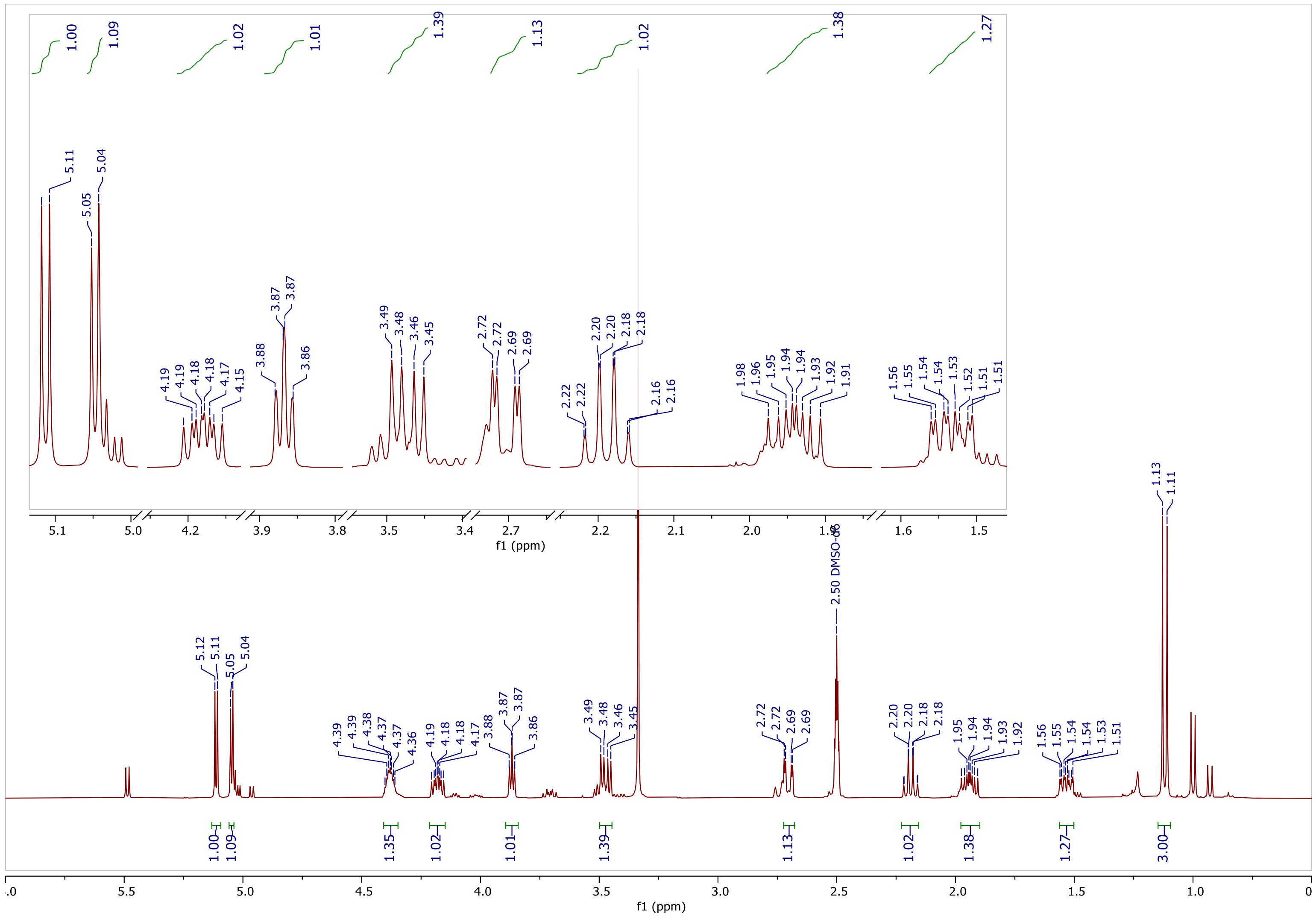

^{13}C, DMSO-d_6

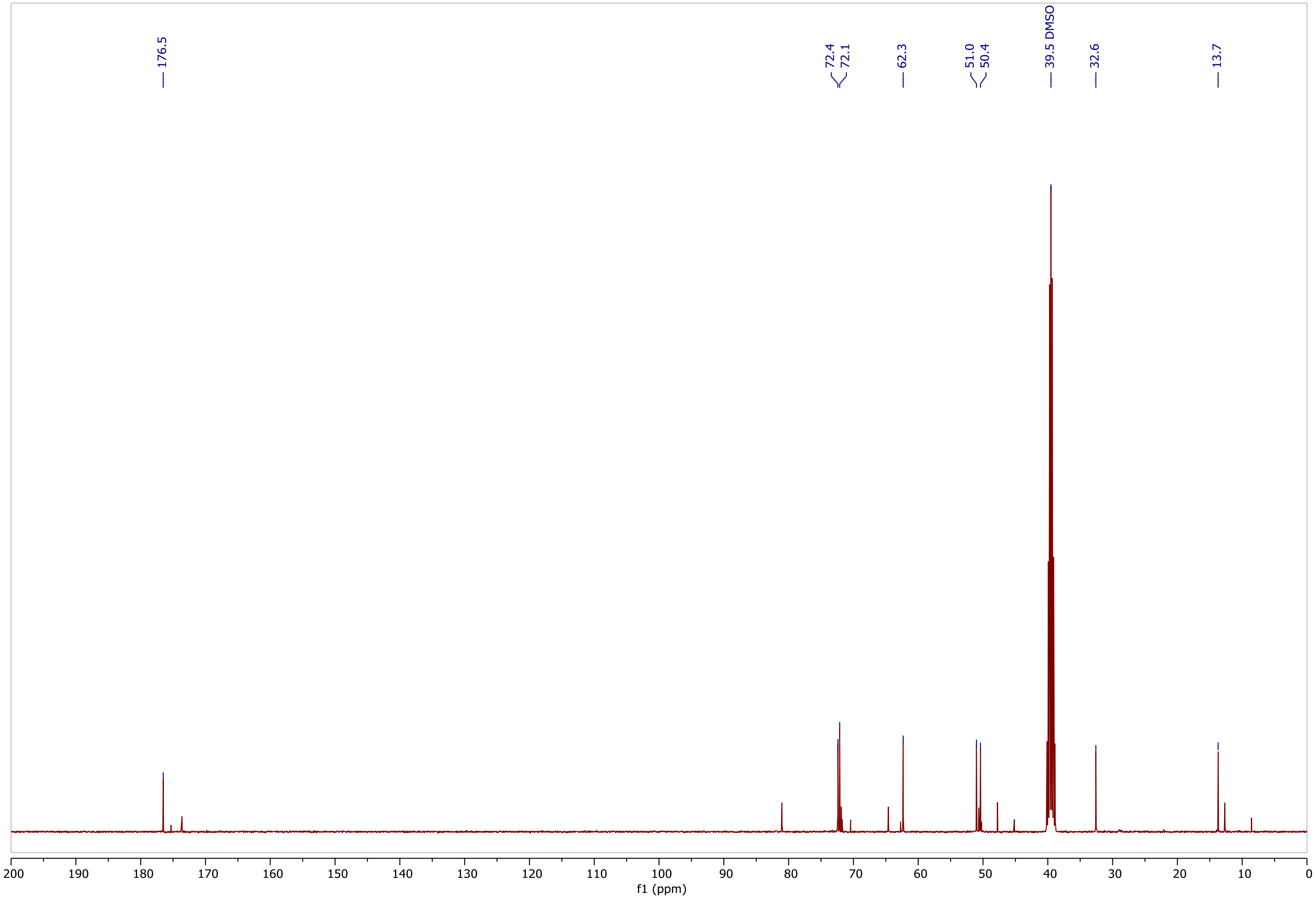

176.5
72.4
72.1
62.3
51.0
50.4
39.5 DMSO
32.6
13.7
200
190
180
170
160
150
140
130
120
110
100
90
80
70
60
50
40
30
20
10
0
f1 (ppm)

(^{1}H-^{1}H)-COSY, DMSO-d$_6$

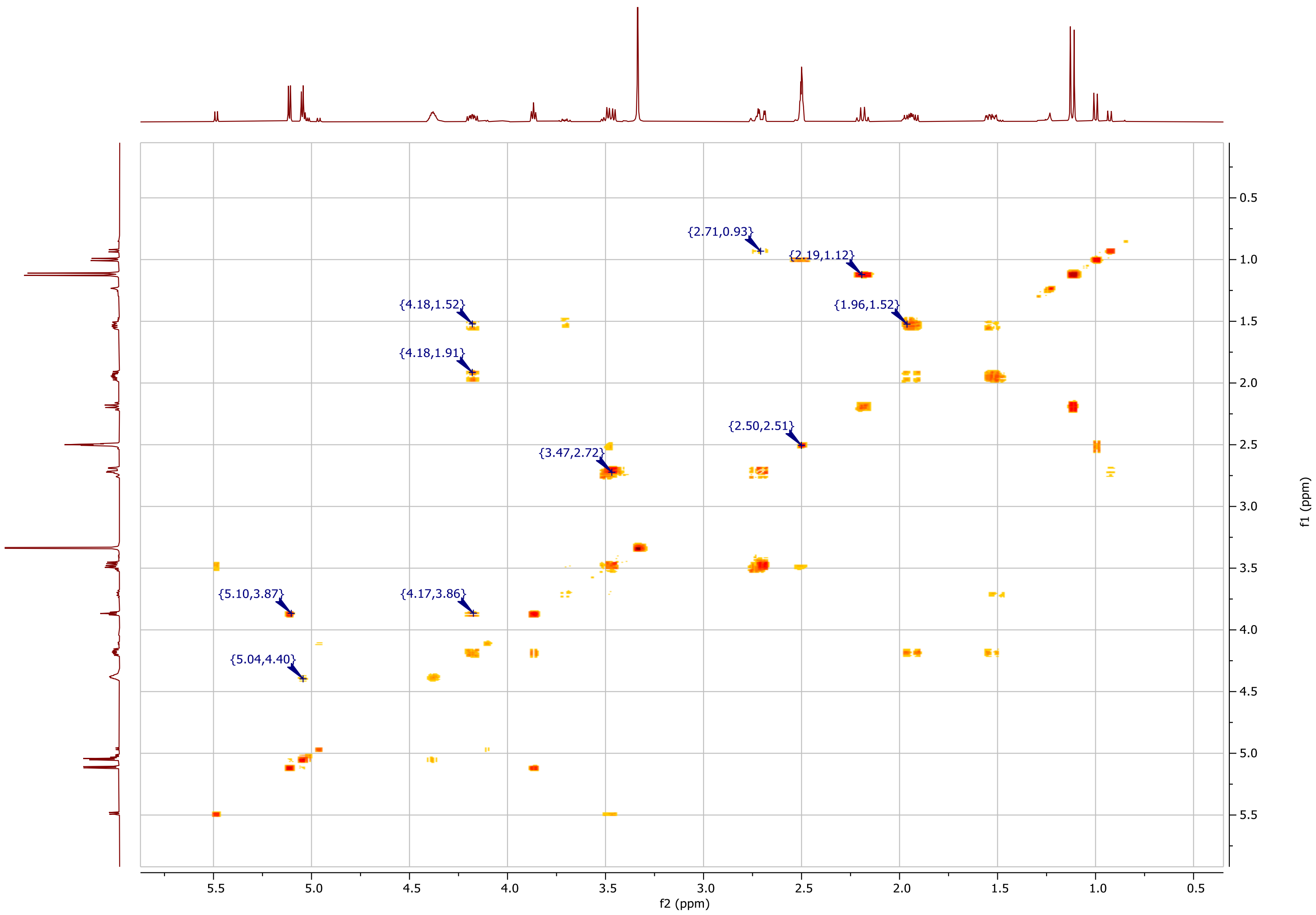

(${}^{1}H-{}^{13}C$)-HSQC, DMSO-d6

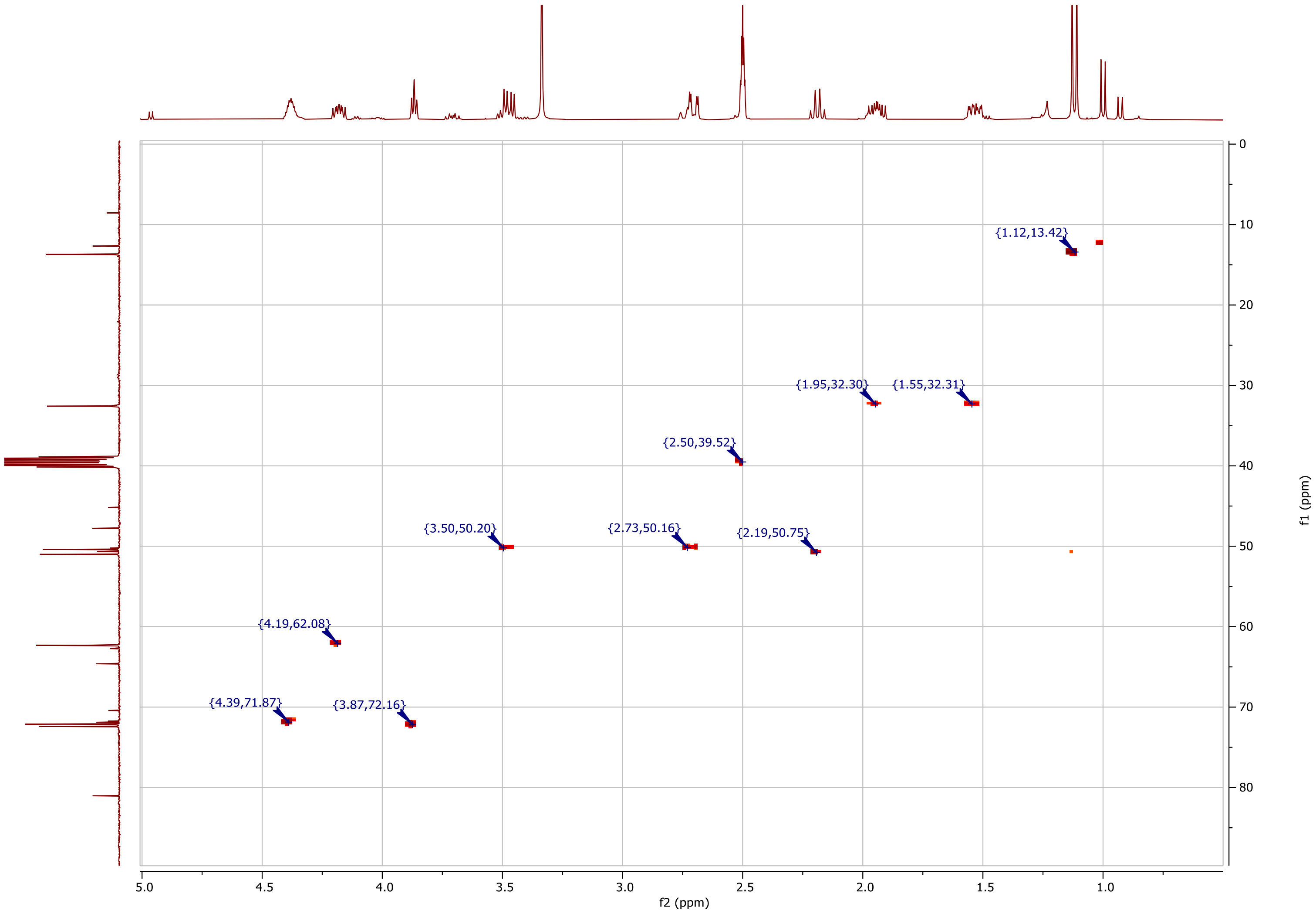

(^{1}H-^{1}H)-NOESY, DMSO-d_6

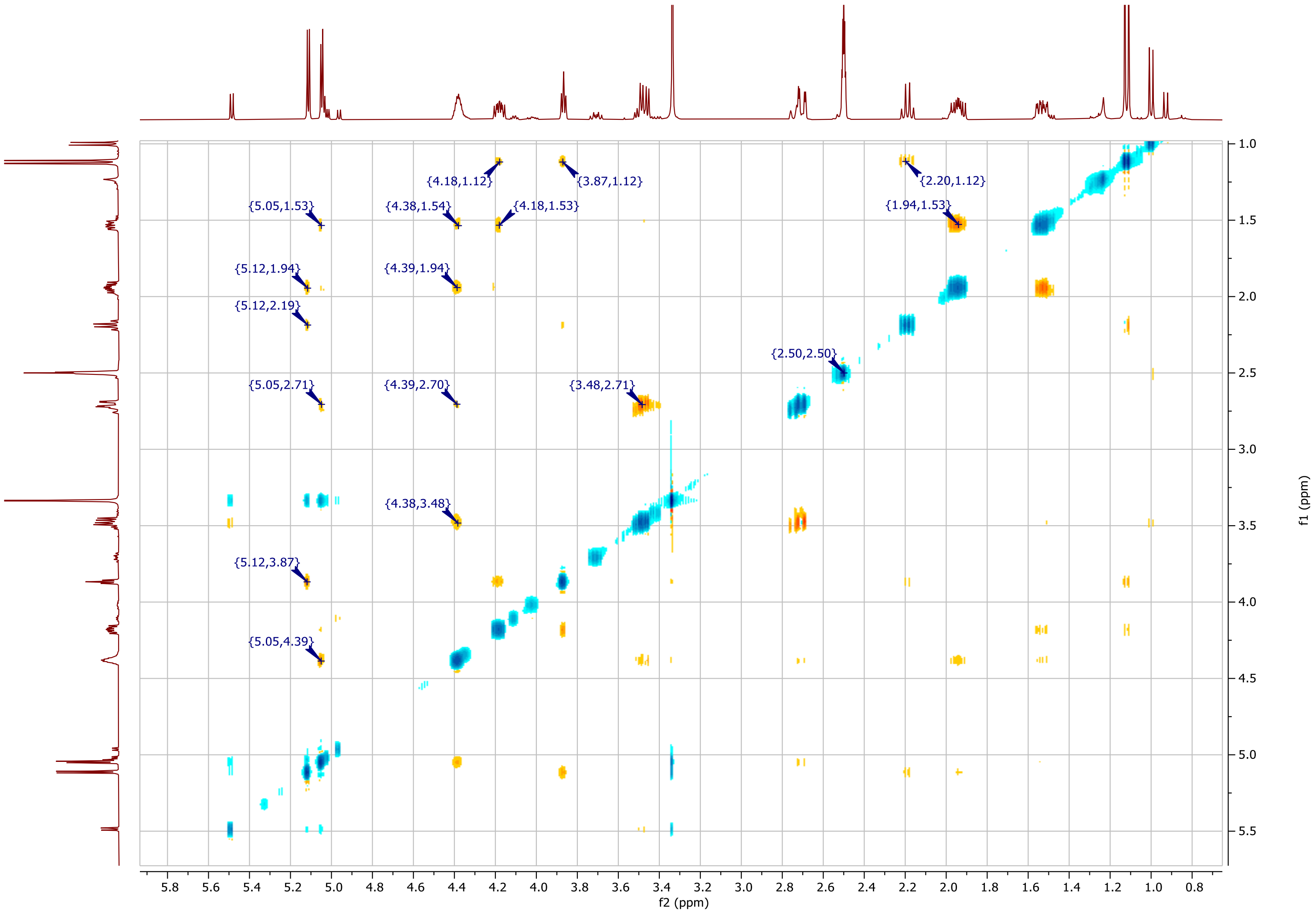

*N*Boc-*O*Ac-β-methoxy-γ-amino acid ethyl ester **8b**

^{1}H, $CDCl_3$

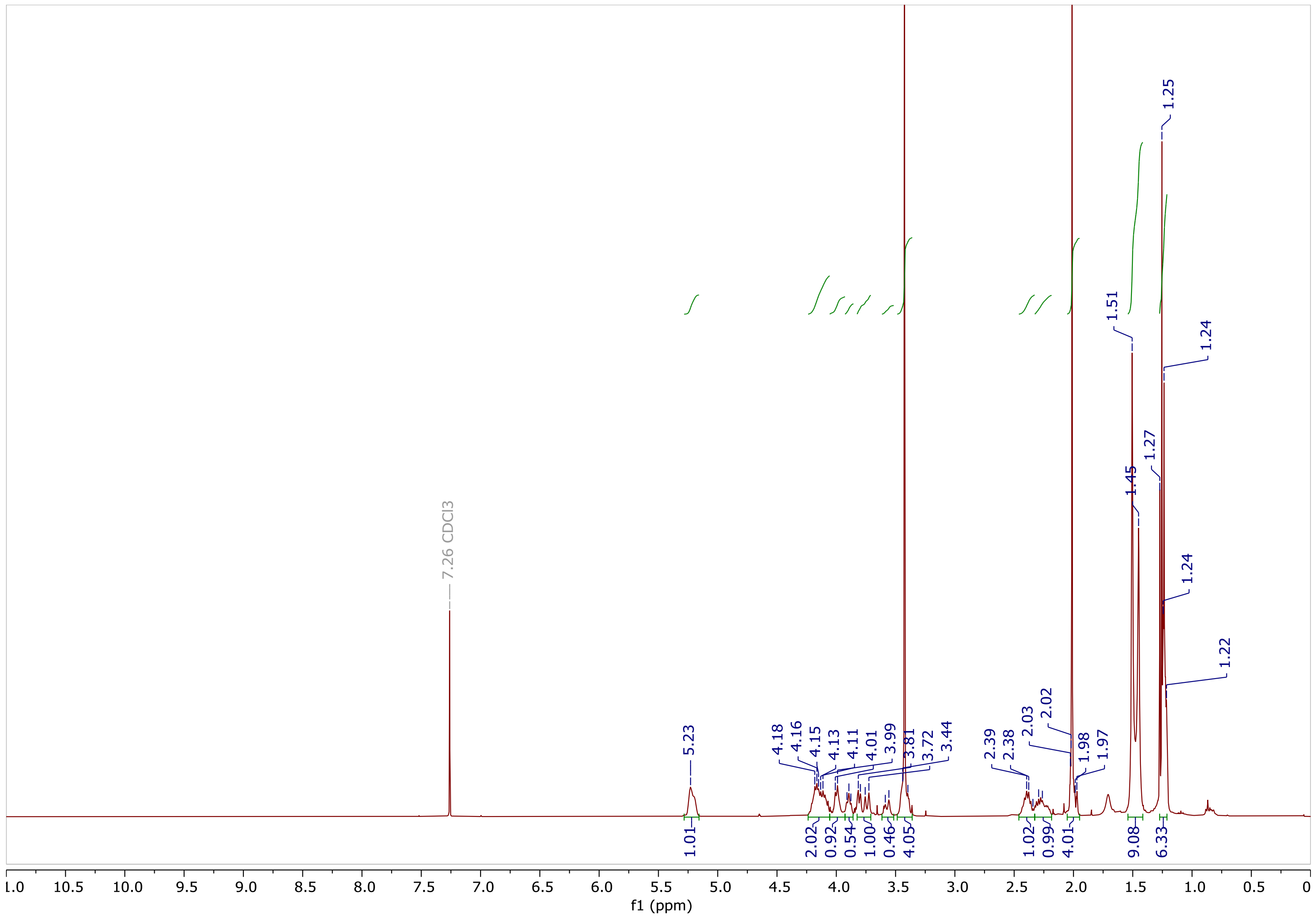

^{13}C, $CDCl_3$

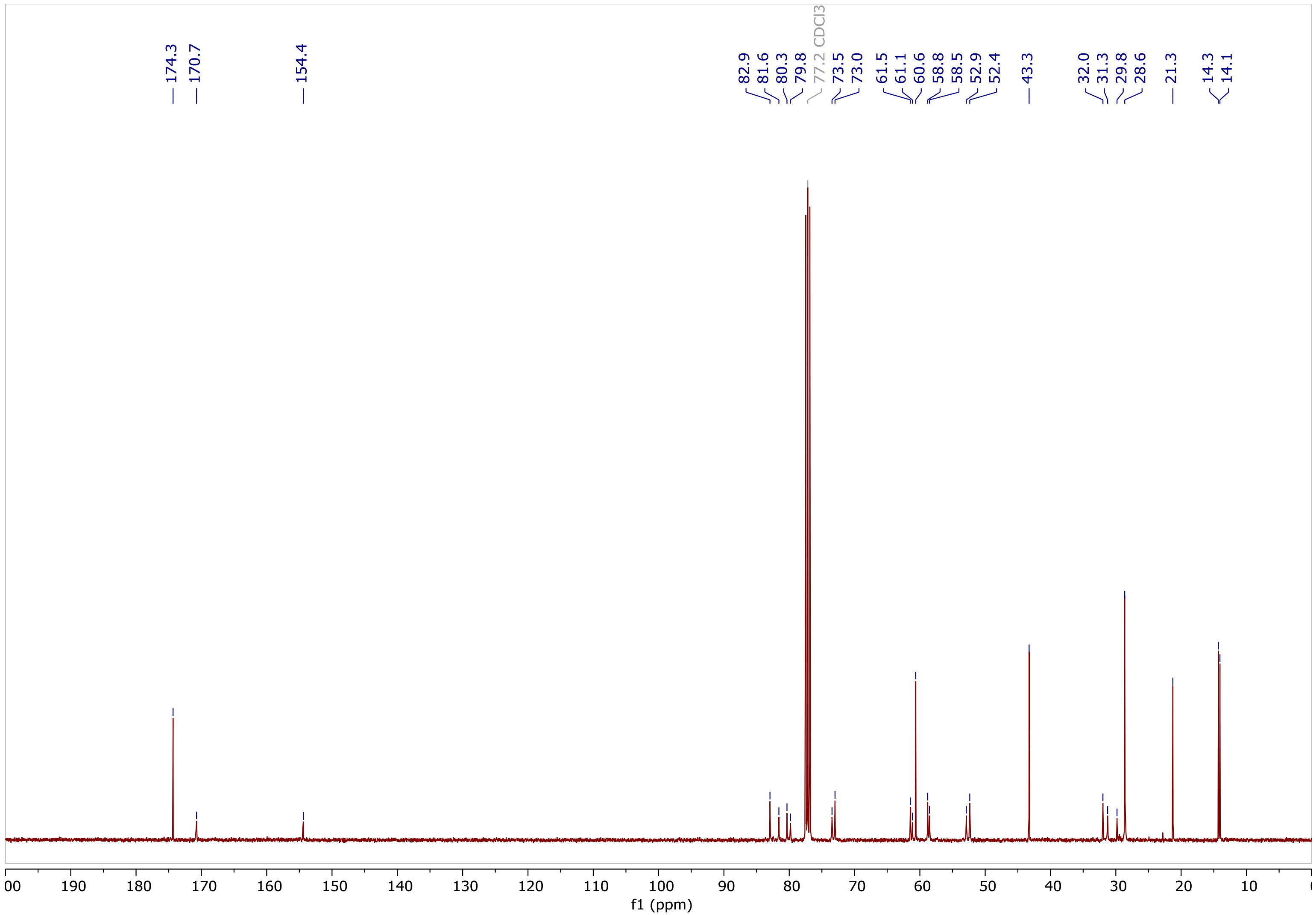

174.3
170.7
154.4
82.9
81.6
80.3
79.8
77.2 CDCl3
73.5
73.0
61.5
61.1
60.6
58.8
58.5
52.9
52.4
43.3
32.0
31.3
29.8
28.6
21.3
14.3
14.1
00
190
180
170
160
150
140
130
120
110
100
90
80
70
60
50
40
30
20
10
f1 (ppm)

*N*Boc-hydroxy-β-methoxy-γ-amino acid ethyl ester

^1H, $CDCl_3$

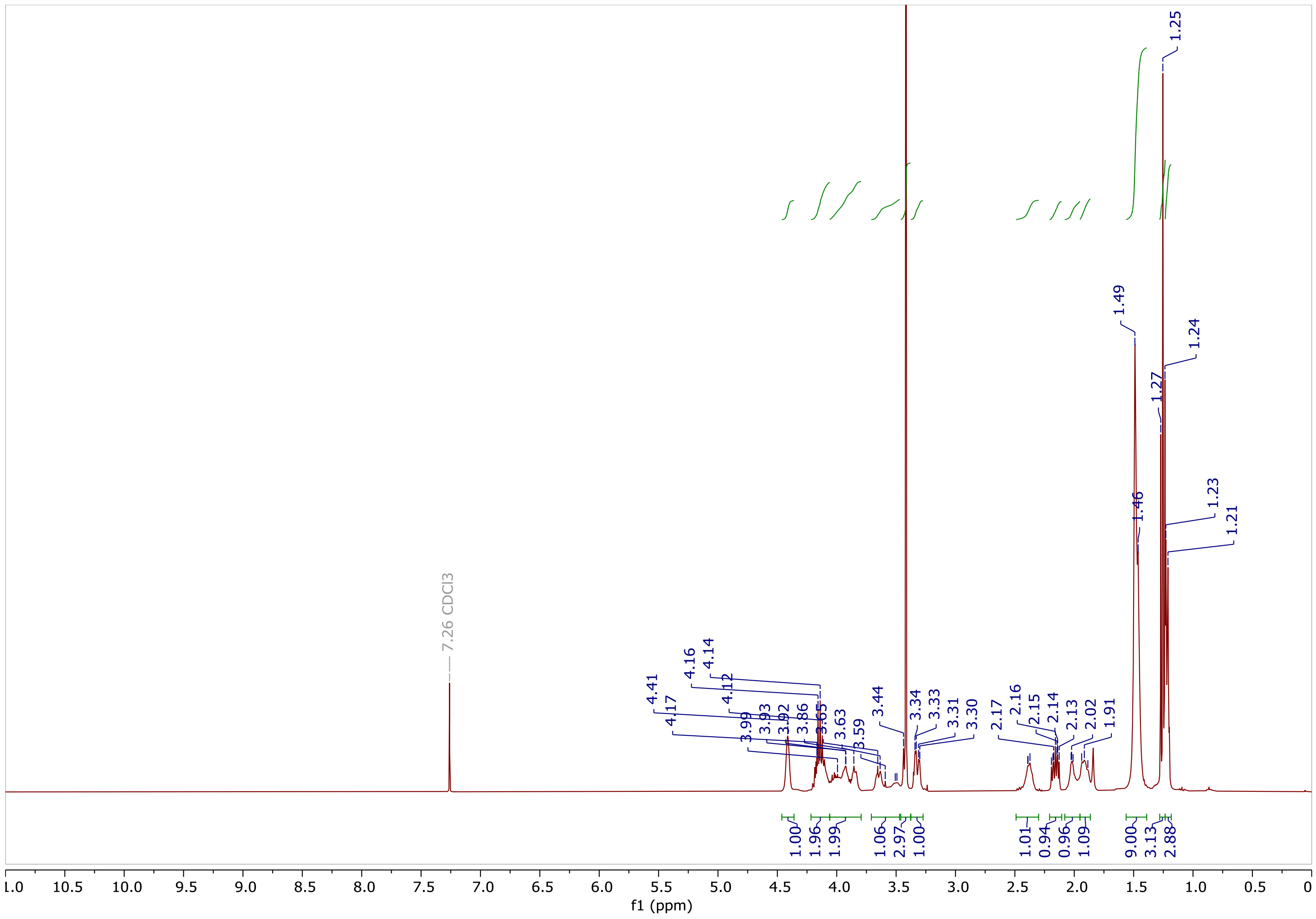

^{13}C, $CDCl_3$

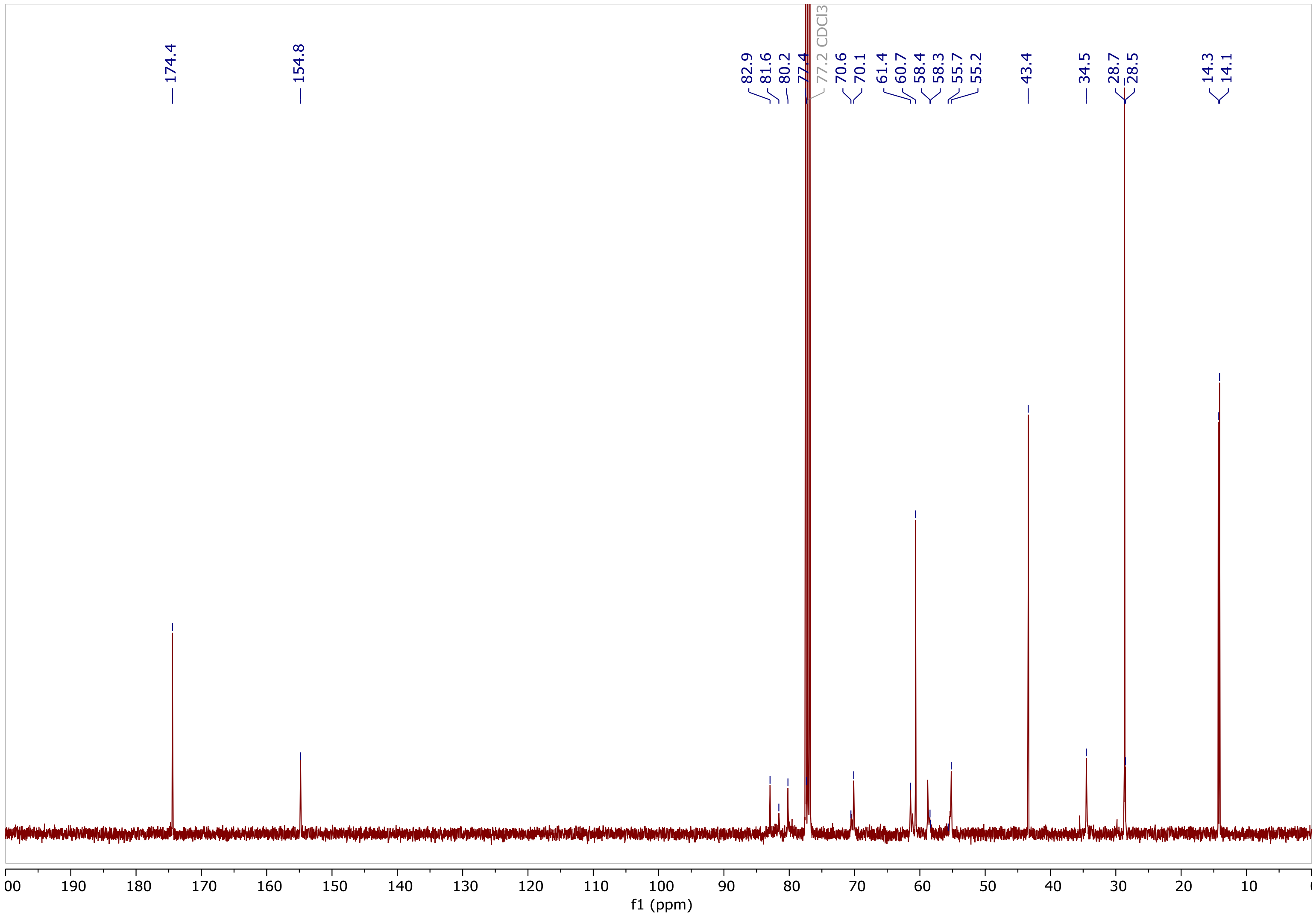
174.4
154.8
82.9
81.6
80.2
77.4
77.2 CDCl3
70.6
70.1
61.4
60.7
58.4
58.3
55.7
55.2
43.4
34.5
28.7
28.5
14.3
14.1
f1 (ppm)

*N*Boc-azido-β-methoxy-γ-amino acid ethyl ester **24**

^{1}H, CD_3CN

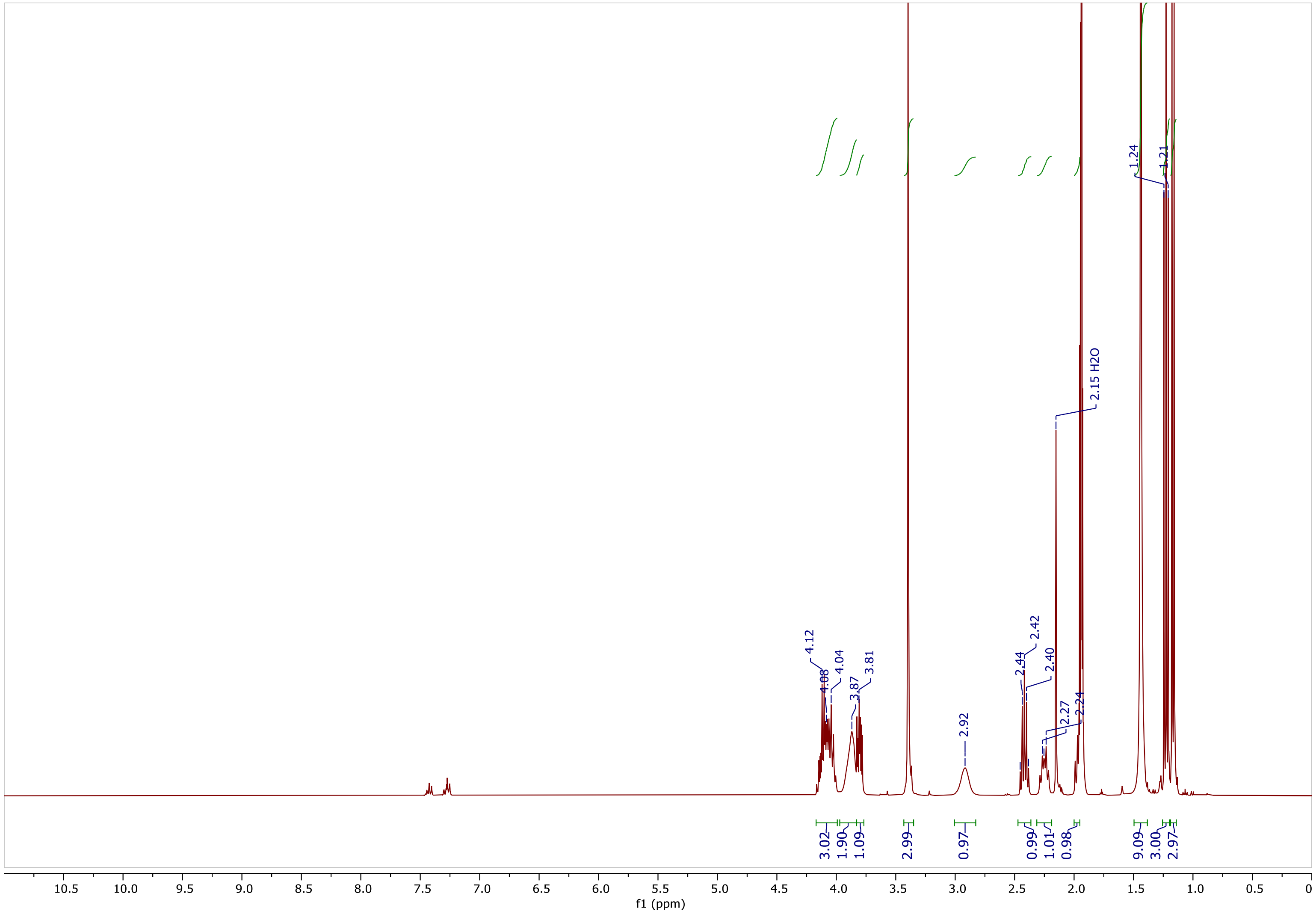

^{13}C, CD_3CN[3]

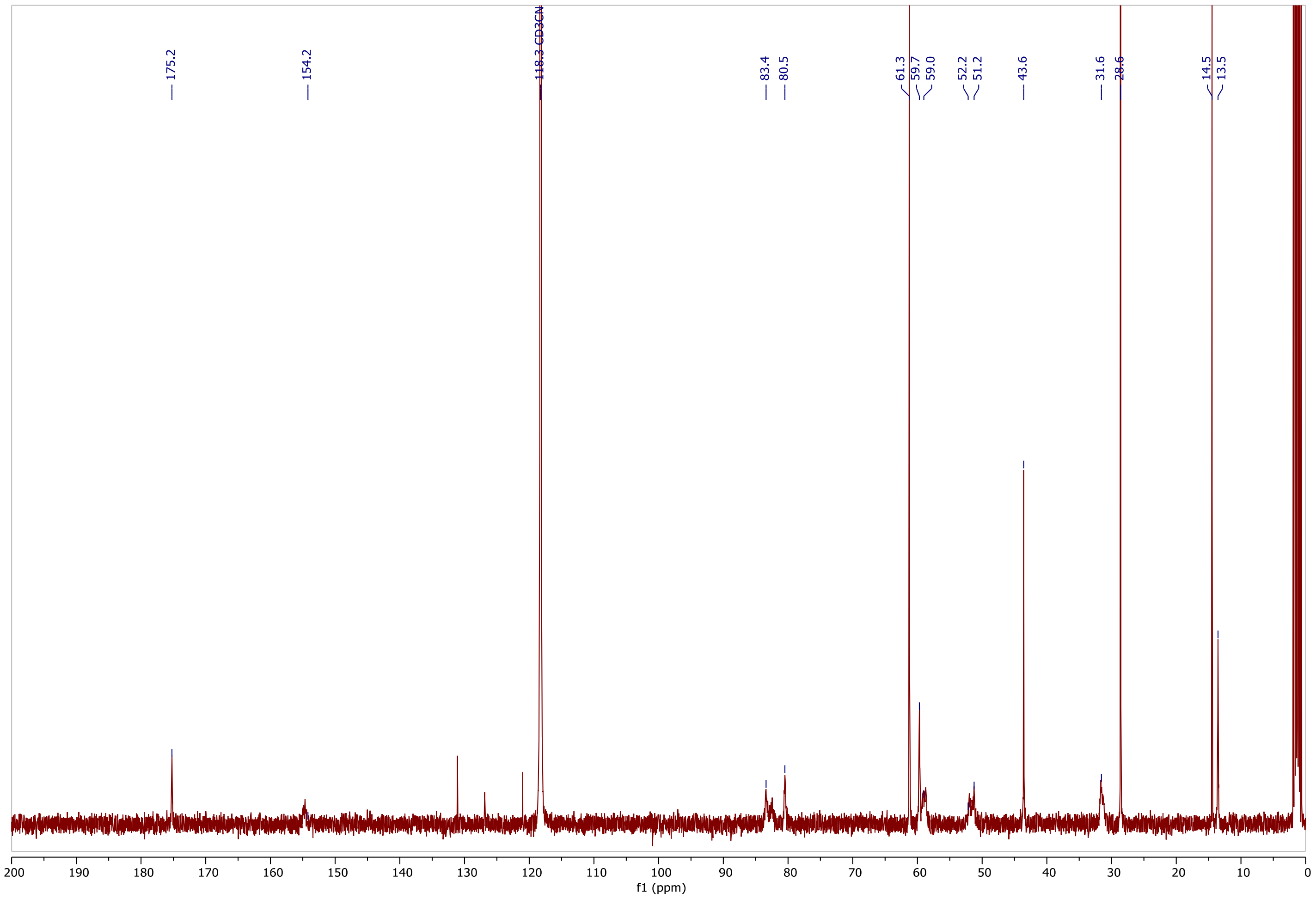

[3] Contaminated with triphenylphosphine oxide (three peaks in the aromatic range visible, see experimental section in core document), but as evident from ^{1}H-NMR spectrum (previous page), the amount was negligible.

*N*Boc-4-(Cbz-amino)Dap-Phe-OMe **26**

^1H, $CDCl_3$

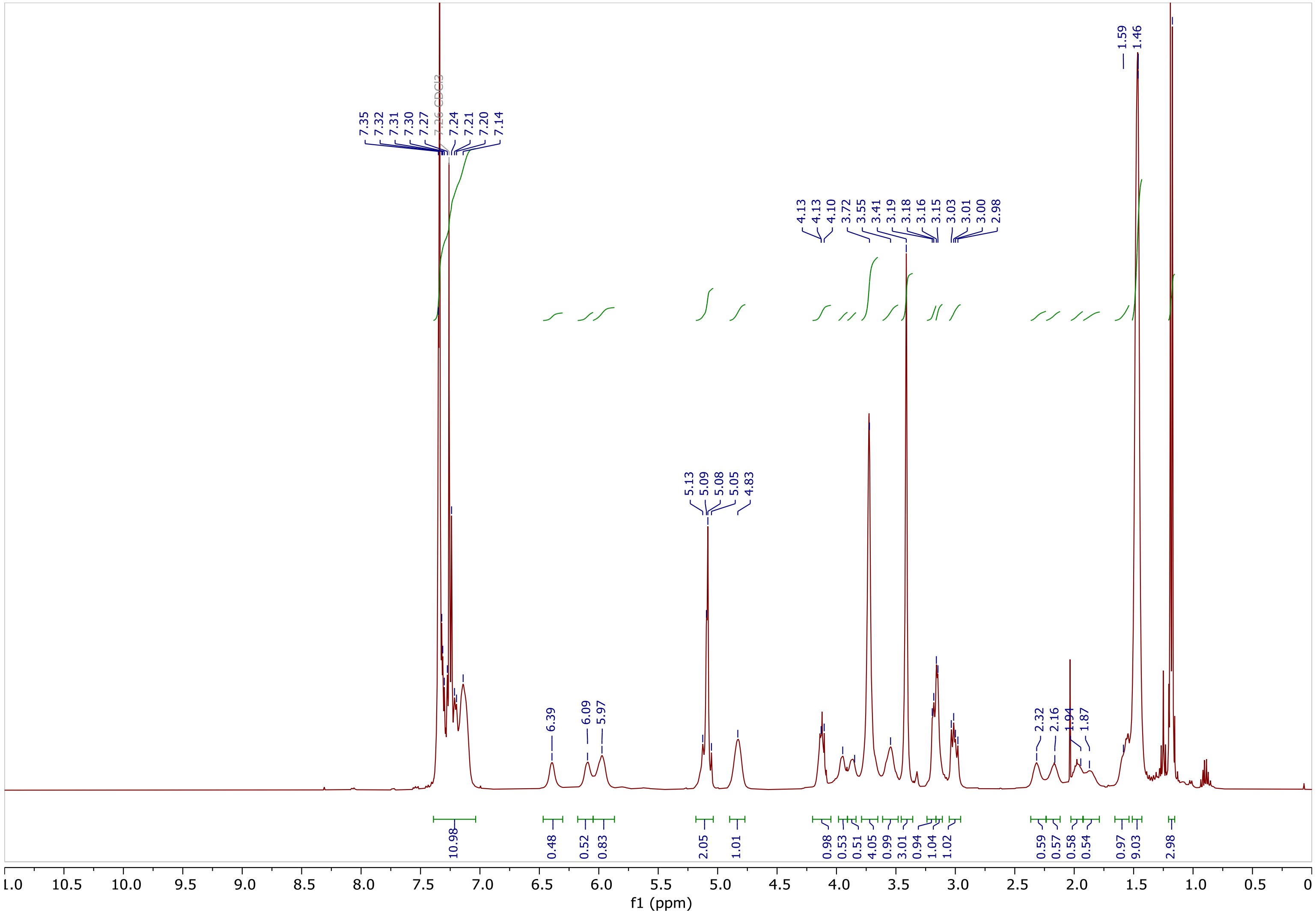

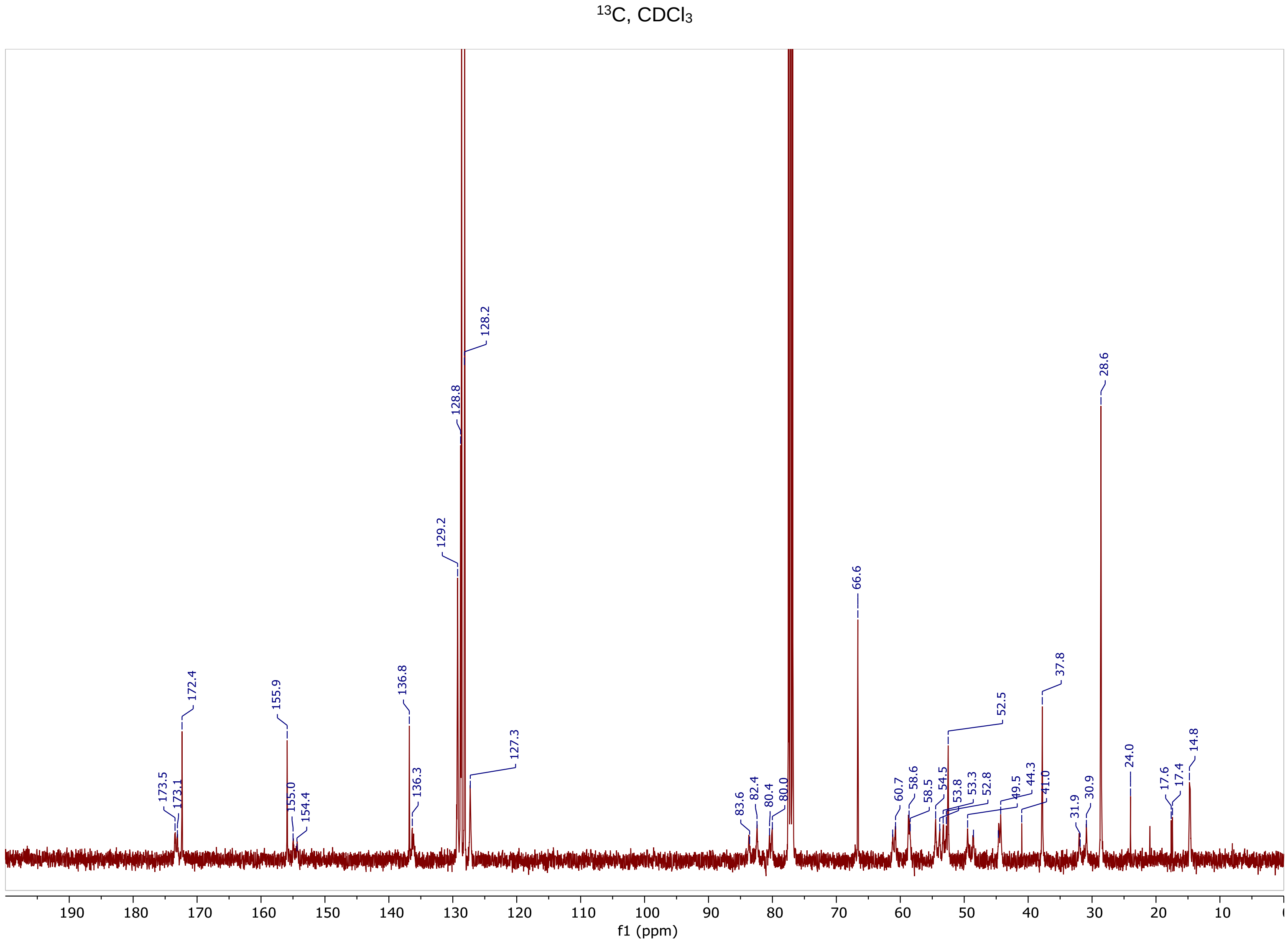

¹³C, CDCl₃
173.5
173.1
172.4
155.9
155.0
154.4
136.8
136.3
129.2
128.8
128.2
127.3
83.6
82.4
80.4
80.0
66.6
60.7
58.6
58.5
54.5
53.8
53.3
52.8
52.5
49.5
44.3
41.0
37.8
31.9
30.9
28.6
24.0
17.6
17.4
14.8
190 180 170 160 150 140 130 120 110 100 90 80 70 60 50 40 30 20 10
f1 (ppm)

IV. NMR and MS spectra: Endgame peptide assembly

Cbz-azastatin methyl ester **27**

^{1}H, CD_2Cl_2

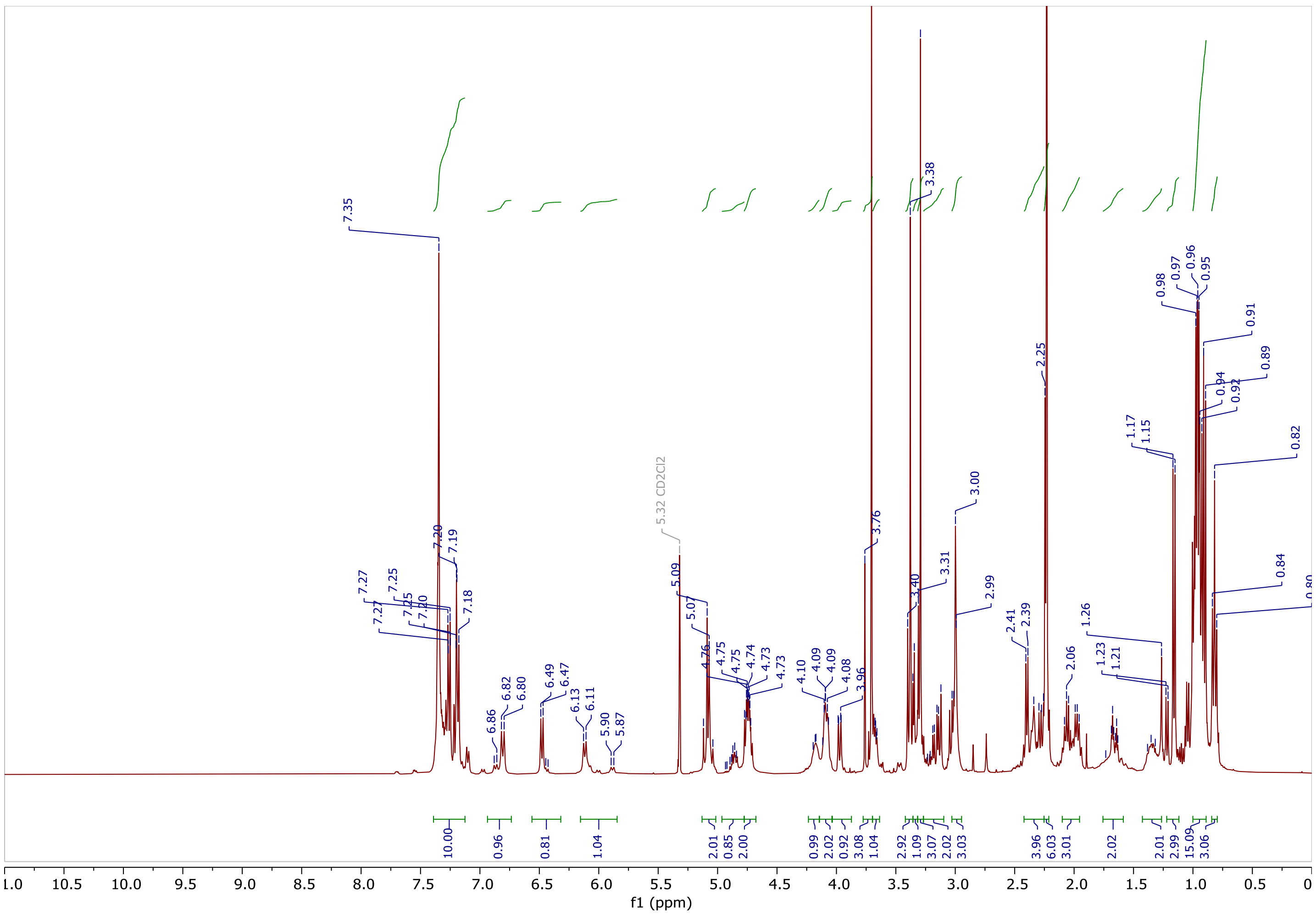

^{13}C, CD_2Cl_2

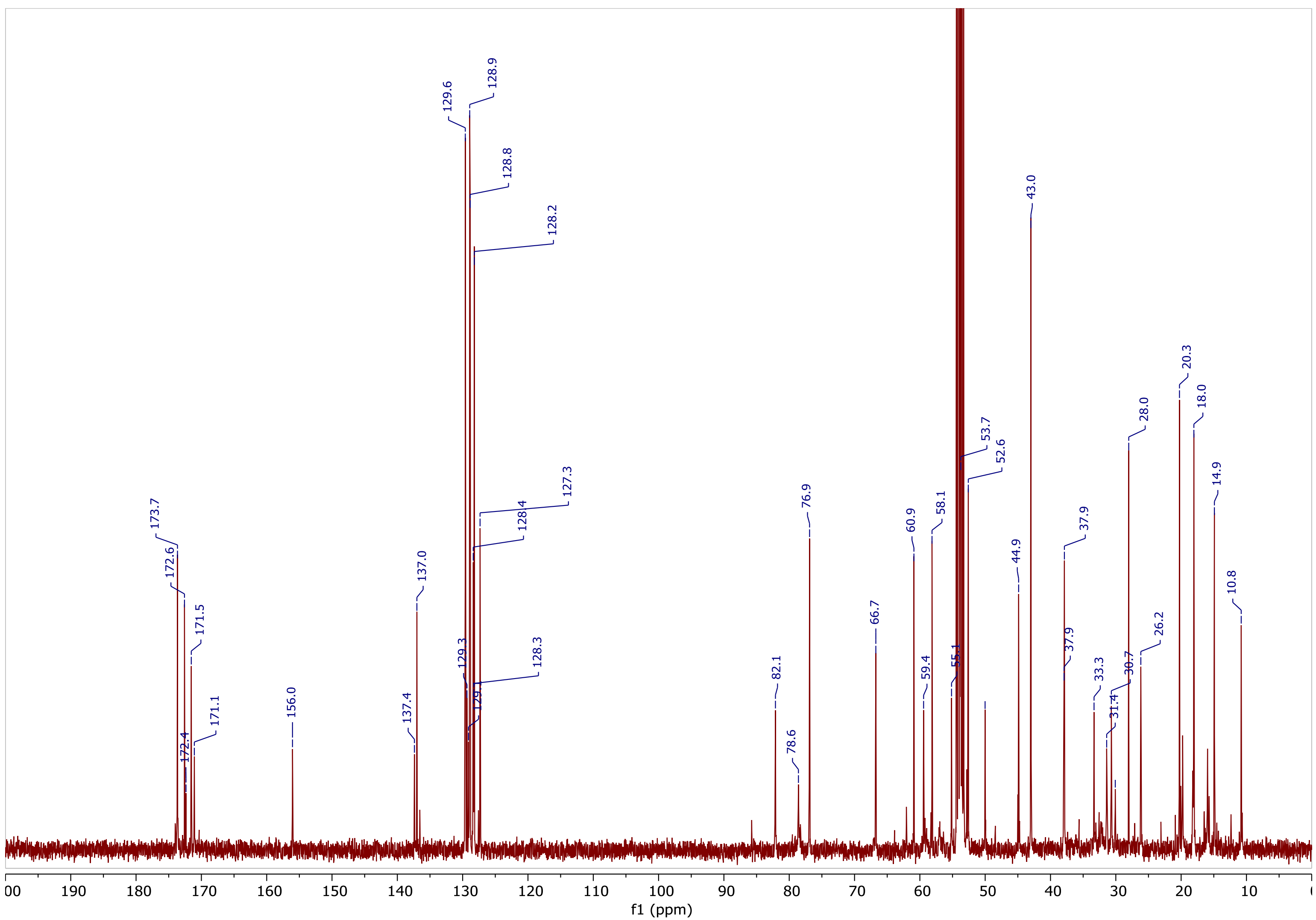
173.7
172.6
172.4
171.5
171.1
156.0
137.4
137.0
129.6
129.3
129
128.9
128.8
128.4
128.3
128.2
127.3
82.1
78.6
76.9
66.7
60.9
59.4
58.1
55.1
53.7
52.6
44.9
43.0
37.9
37.9
33.3
31.4
30.7
28.0
26.2
20.3
18.0
14.9
10.8
f1 (ppm)

Azastatin methyl ester **7**

^{1}H, CD_3OD

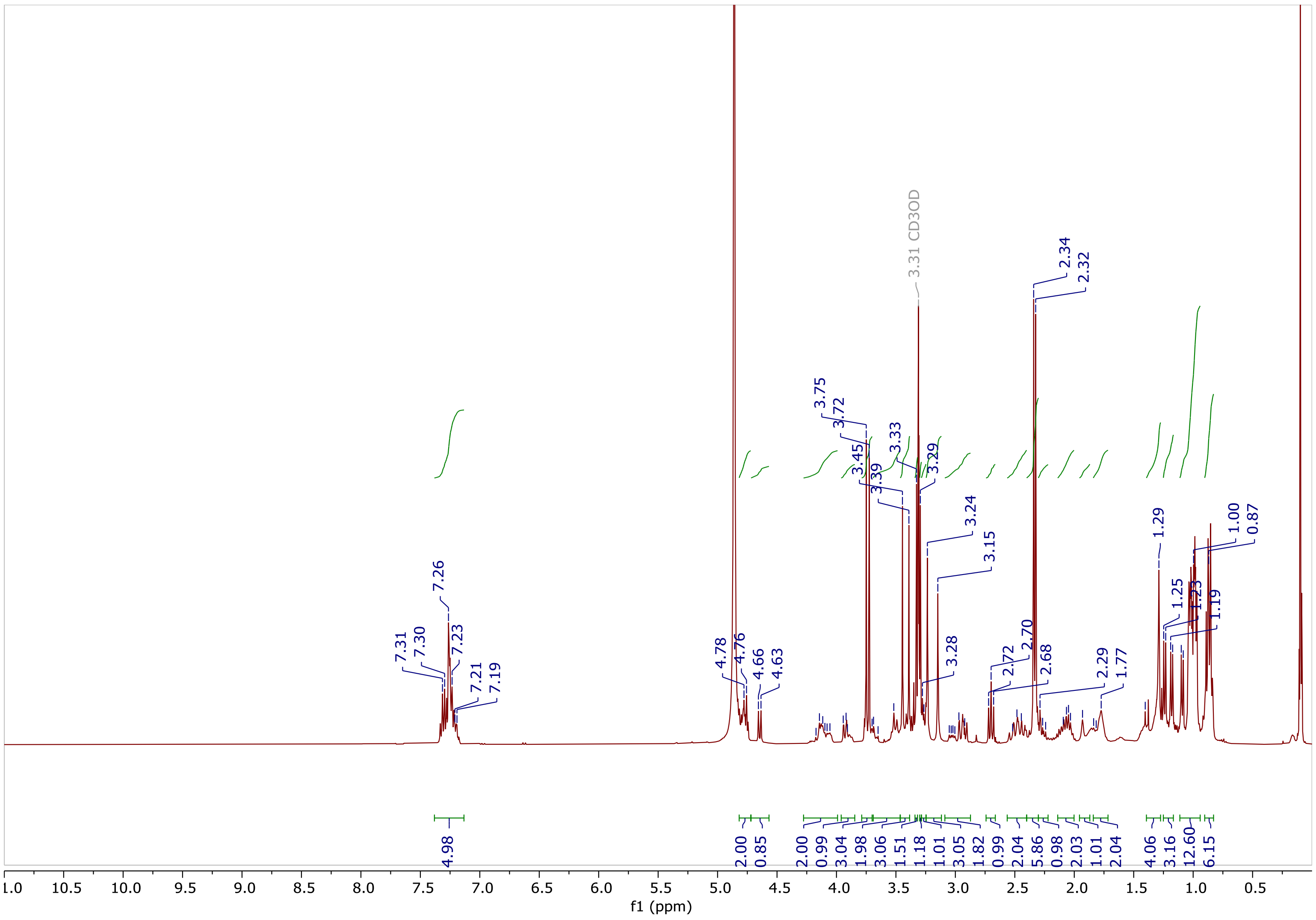

^{13}C, CD_3OD

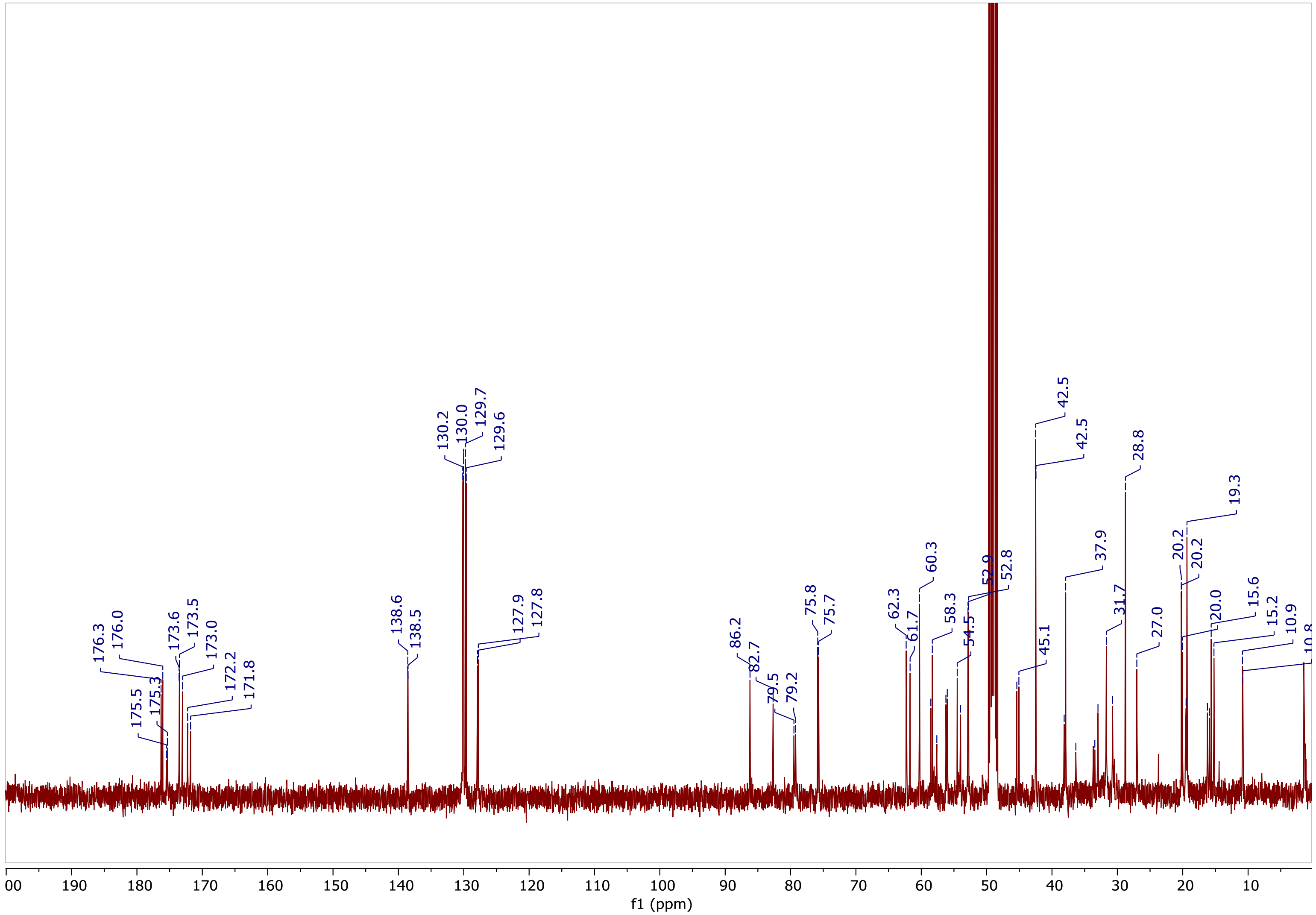
176.3
176.0
175.5
175.3
173.6
173.5
173.0
172.2
171.8
138.6
138.5
130.2
130.0
129.7
129.6
127.9
127.8
86.2
82.7
79.5
79.2
75.8
75.7
62.3
61.7
60.3
58.3
54.5
52.9
52.8
45.1
42.5
42.5
37.9
31.7
28.8
27.0
20.2
20.2
19.3
20.0
15.6
15.2
10.9
10.8
00
190
180
170
160
150
140
130
120
110
100
90
80
70
60
50
40
30
20
10
f1 (ppm)

Mass-spectrometric analysis

Structure	Sum formula	m/z		
		Theoretical	Found	Δ (relative)
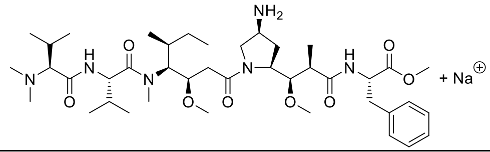	$C_{41}H_{70}N_6NaO_8^+$	797.5147	797.5112	4.4ppm
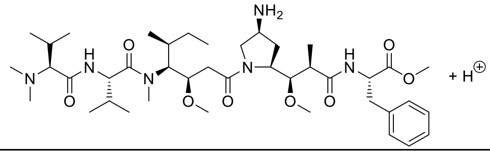	$C_{41}H_{71}N_6O_8^+$	775.5328	775.5266	8.0ppm
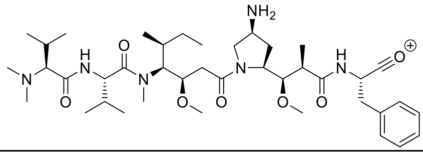	$C_{40}H_{67}N_6O_7^+$	743.5066	743.4967	13.3ppm
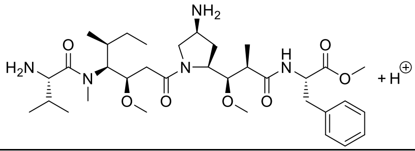	$C_{34}H_{58}N_5O_7^+$	648.4331	648.4424	14.3ppm
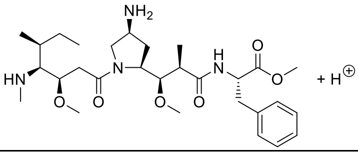	$C_{29}H_{49}N_4O_6^+$	549.3647	549.3580	12.2ppm
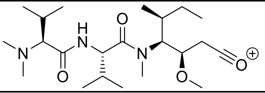	$C_{22}H_{42}N_3O_4^+$	412.3170	412.3120	12.1ppm
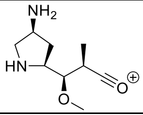	$C_9H_{17}N_2O_2^+$	185.1285	185.1279	3.2ppm

Low collision energy HRMS

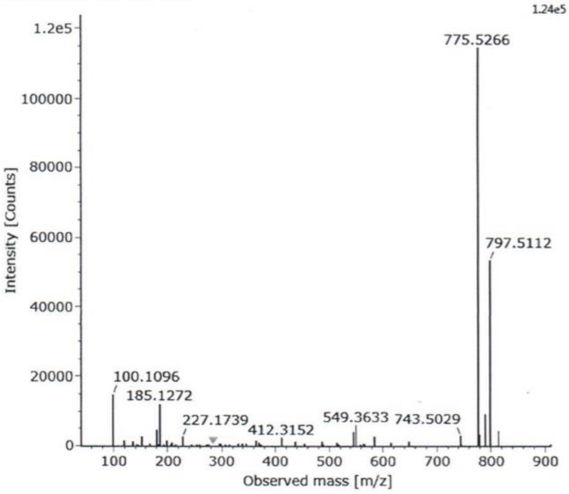

High collision energy HRMS

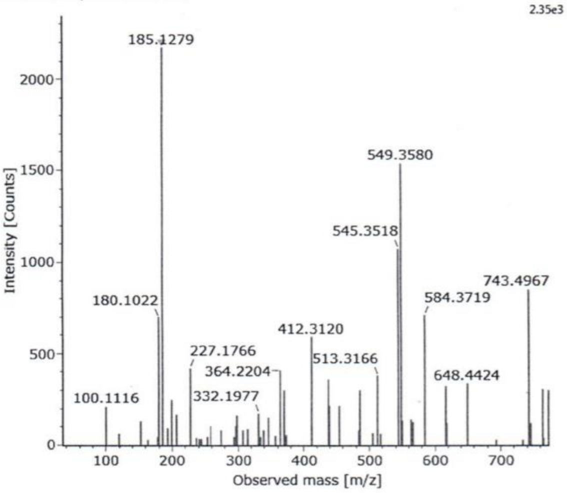

N-ethyl azastatin methyl ester **28**

^{1}H, CD_3OD

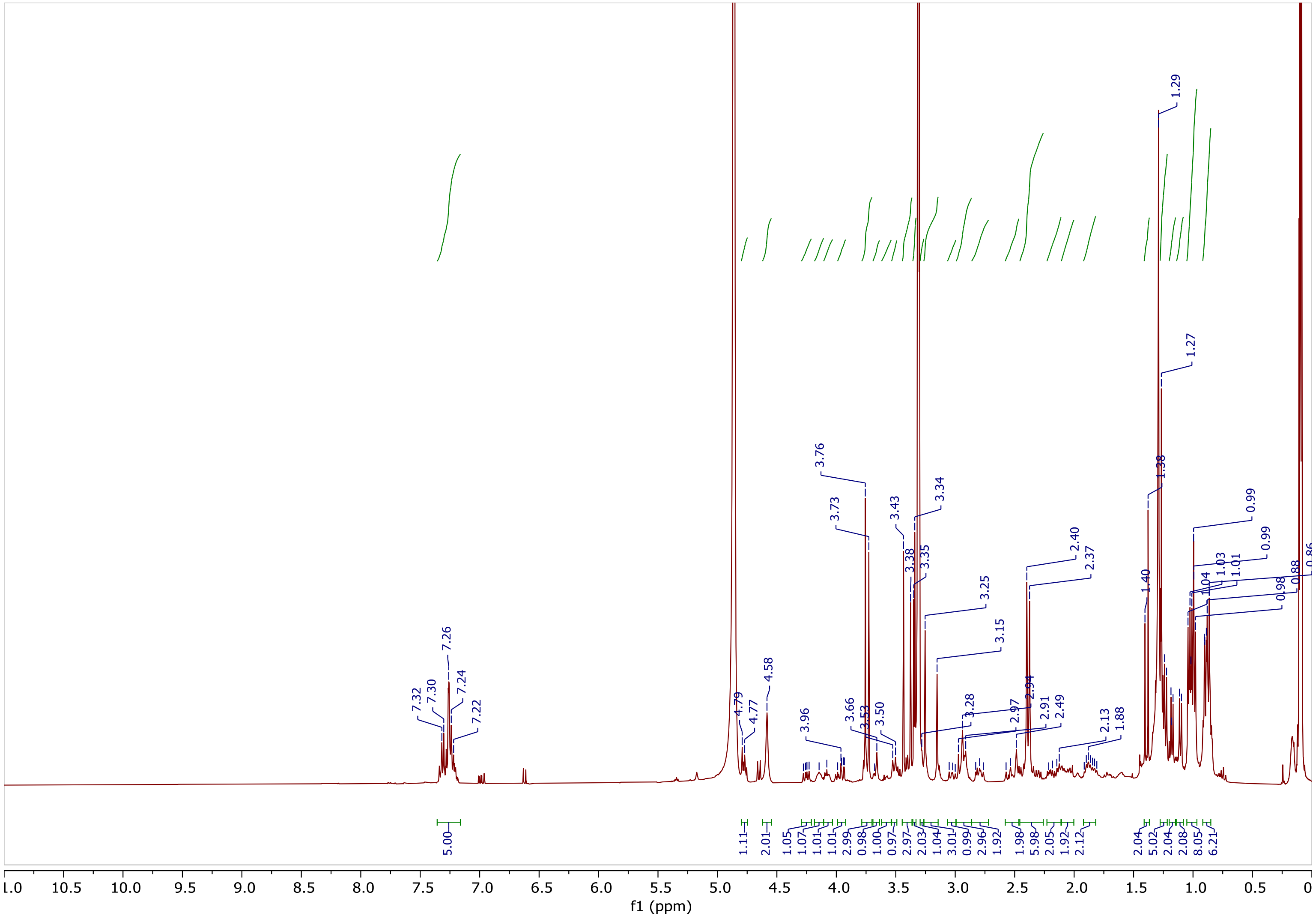

Mass-spectrometric analysis

Structure	Sum formula	m/z		
		Theoretical	Found	Δ (relative)
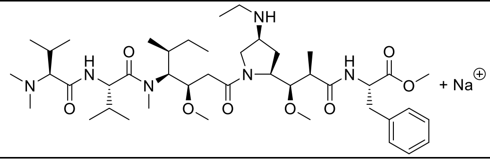	$C_{43}H_{74}N_6NaO_8^+$	825.5460	825.5413	5.7ppm
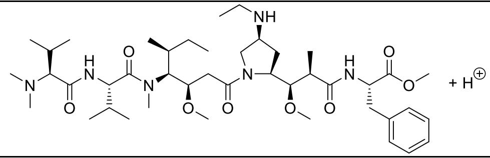	$C_{43}H_{75}N_6O_8^+$	803.5641	803.5579	7.7ppm
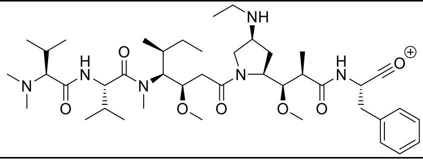	$C_{42}H_{71}N_6O_7^+$	771.5379	771.5225	20.0ppm
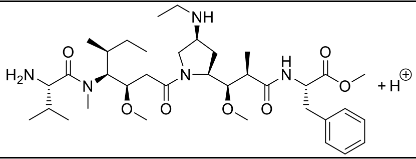	$C_{36}H_{62}N_5O_7^+$	676.4644	676.4607	5.5ppm
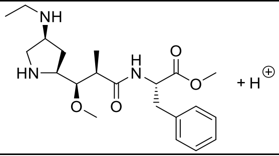	$C_{21}H_{34}N_3O_4^+$	392.2544	392.2490	13.7ppm
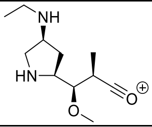	$C_{11}H_{21}N_2O_2^+$	213.1598	213.1593	2.3ppm

Low collision energy HRMS

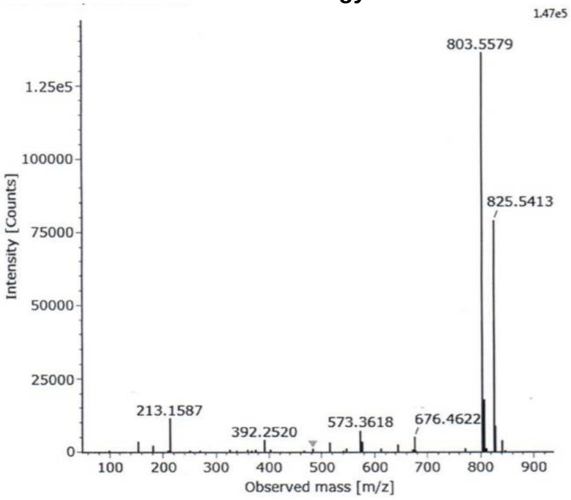

High collision energy HRMS

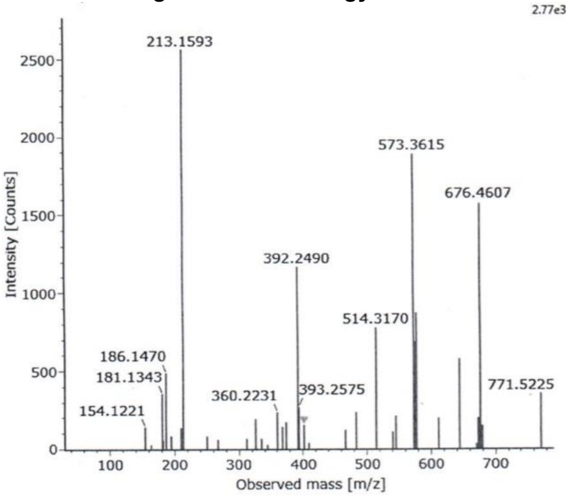

Supplement: Supplementary file 1 — Supplementary [file CMDC-15-2500-s001.pdf]
